# Consumers’ Attitudes towards Animal Suffering: A Systematic Review on Awareness, Willingness and Dietary Change

**DOI:** 10.3390/ijerph192316372

**Published:** 2022-12-06

**Authors:** Rui Pedro Fonseca, Ruben Sanchez-Sabate

**Affiliations:** 1 Centro de Investigação e Estudos de Sociologia Iscte, Instituto Universitário de Lisboa, 1649-026 Lisbon, Portugal; 2 Centro de Excelencia en Psicología Económica y del Consumo (CEPEC), Núcleo Científico y Tecnológico en Ciencias Sociales y Humanidades, Universidad de La Frontera, Temuco 4780000, Chile; 3 Núcleo de Investigación en Educación, Ciencias Sociales y Patrimonio, Universidad Adventista de Chile, Chillán 3820572, Chile

**Keywords:** systematic review, consumer attitudes, animal welfare, animal suffering, dietary change, meat consumption, behavior interventions, planetary health, human health, concerns, animals, contentious farming, husbandry

## Abstract

Planetary and human health depend on Westerners’ ability to reduce meat consumption. Meat production degrades the environment while excessive meat intake is associated with cancer and cardiovascular disease, among others. Effective reasons and motivations are needed for consumers to change their diet. The fact that modern animal agriculture inflicts a great deal of pain on animals from their birth to their slaughter, animal welfare/suffering may drive consumers to curtail their meat consumption. This systematic review examined a total of 90 papers to ascertain consumers’ awareness of the pain animals experience in animal agriculture, as well as consumer attitudes towards meat reduction due to animal welfare. Results show that consumers have low awareness of animal agriculture. Awareness of animal agricultural practices and animal sentience is associated with increased negative attitudes towards animal suffering. Animal suffering due to farming practices, transportation, slaughter, and animal sentience are factors that may encourage a reduction in meat consumption, and even dietary change in the short term. There is also evidence that animal suffering may be a more compelling motivation for consumers’ willingness to change their diet than for health or environmental reasons. Therefore, increasing consumers’ awareness of animal suffering in meat production is paramount to contributing to reduced pressure on the environment and improved human health.

## 1. Introduction

The increasing world levels of meat consumption [1,2] poses a great threat to achieving food systems compatible with planetary and human health. Animal agriculture is among the main factors causing global warming and environmental degradation [1,3,4,5,6]. The livestock industry is a major source of anthropogenic greenhouse gas emissions, pollutes freshwater with antibiotics, hormones, and chemical substances, among others, and depletes freshwater availability [1]. Excessive red and/or processed meat intake is associated with cancer [7,8,9], chronic diseases [10,11], cardiovascular disease [12], and all-cause mortality, among others [12,13,14,15]. Therefore, reducing meat consumption is key to significantly mitigating climate change [16] and securing healthy and sustainable diets for an estimated global population of 10 billion people by 2050 [17].

Although plant-based foods are more sustainable, and less harmful for animals and the environment [18,19,20], meat-based diets are still the norm in the West. The percentage of vegetarians ranges from about 2% to around 10% in North America and Western Europe [21,22]. Consumers adopting a vegan or vegetarian diet are usually motivated by non-static reasons related to human and planetary health, economy, and sociocultural and religious values [23]. The most prevalent motivations among vegetarians are health and animal welfare [24,25,26,27,28]. These motivations may also be the most common among meat-reducers [29], a.k.a. flexitarians or semi-vegetarians, a group of consumers rarely studied [30] until very recently [31]. The percentage of Westerners with positive attitudes to reducing meat consumption and/or who are already flexitarian might be higher than believed. According to around 20 studies conducted in 2019 or later in Australia, the US, Canada and Western Europe, the percentage of consumers considering or already practicing any of the many forms a meat-reduced diet can take, ranges from as low as 11% to as high as 66% [31]. This might be a signal of a changing tide in Western dietary patterns but, still, changing eating practices is always challenging due to, for example, social norms, culinary traditions and taste preferences [32].

The Transtheoretical Model (TTM) of health behavior change [33] describes a three-stage progression that comprises awareness (precontemplation), willingness (contemplation and preparation), and change (action, maintenance, and termination). This process of change can only occur with the adoption of a positive attitude based on reasons and motivations [33]. Recent psychological research on meat consumption argues that animal welfare motives and reasons may be powerful stimuli to prompt consumers to reduce meat intake or at least develop a more favorable view towards it [34,35]. A recent meta-analysis on interventions to reduce meat intake due to animal welfare showed that they appear to be effective in curbing meat consumption. Even though the analyzed interventions were short-term studies of self-reported outcomes with a high risk of social desirability bias [36], this, and other evidence already cited, indicate that animal welfare must be considered when searching for reasons and motives to promote meatless or meat-reduced diets [37].

Animal welfare is a complex issue that comprises animals’ physiology, psychology, and environment [38]. Depending on the competing interests of different animal production system stakeholders, animal welfare definitions will emphasize one aspect more over the others [38]. Two recent papers present a brief history and problematization of this concept [38,39]. In this paper, we understand animal welfare as the globally recognized welfarist Five Freedoms from the Brambell Committee: 1. Freedom from hunger and thirst; 2. Freedom from discomfort; 3. Freedom from pain; 4. Freedom to express normal behavior; 5. Freedom from fear and distress [40].

Current animal agriculture is far from this ideal, as evidenced by a considerable body of research [41]. Most animals are bred in overcrowded and confined systems, unable to breathe fresh air, walk, and access land [42], causing a high incidence of several diseases [43,44,45,46,47,48,49,50,51]. Animals can be regularly tethered [52] in gestation crates [53] and battery cages [54,55], are subject to several types of mutilations without anesthetics [56,57,58], and separated early from their offspring [59,60]. Transportation leads to severe stress, can cause several injuries, and even the death of animals [61]. Before slaughter, animals might experience prolonged thirst and hunger, pain, fear, and respiratory distress [62]. Poorly designed equipment, the use of electric prods, untrained personnel [63], and rough handling [62] in slaughter plants have negative consequences for animals. Although there is a scientific consensus in recognizing non-human animals used for food as sentient beings [40,64,65,66,67], these summarized contentious [68,69,70,71,72] practices refute the assumptions that animals experience a “good life”, or are subject to “good treatment” [73] in most production systems, during transport and slaughter.

Previous research shows evidence that consumers agree with improved/welfarist animal farming systems [43,74,75]. Although their level of knowledge about farming and slaughter is relatively low [38,76], they tend to share the impression that the living conditions of farmed animals are far from optimal [77], especially in conventional production systems [78]. The most expected attitudes are indifference [79], the dissociation of meat from the animal, or even the avoidance of thinking about the upsetting production processes involved [43,68,80]. Women and younger people usually have higher levels of concern about animal welfare [81].

This systematic review aims to increase our understanding about consumers’ attitudes towards animal welfare/animal farming practices. Based on the TTM health behavior change, we aimed to answer the following questions: *(A1)* Are consumers aware of the pain animals endure in animal agriculture? *(A2)* What are their attitudes towards modern animal agriculture? *(B)* Are they willing to reduce or replace animal-based products because of animal welfare? *(C)* Have they reduced or stopped the intake of animal products due to animal welfare?

Finally, we present the evidence about strengths and limitations of current published work, and we discuss potential directions for future research.

## 2. Materials and Methods

This research project adopted the Open Science principles established to promote openness, integrity, and reproducibility in research [82]. Therefore, its study design was made public through the Open Science Framework (OSF) in July 2022 [83]. OSF is a free and open source web platform that helps scholars with their research process and allows for the management of data that is open, transparent, and citable [82,84].

**Figure 1:**
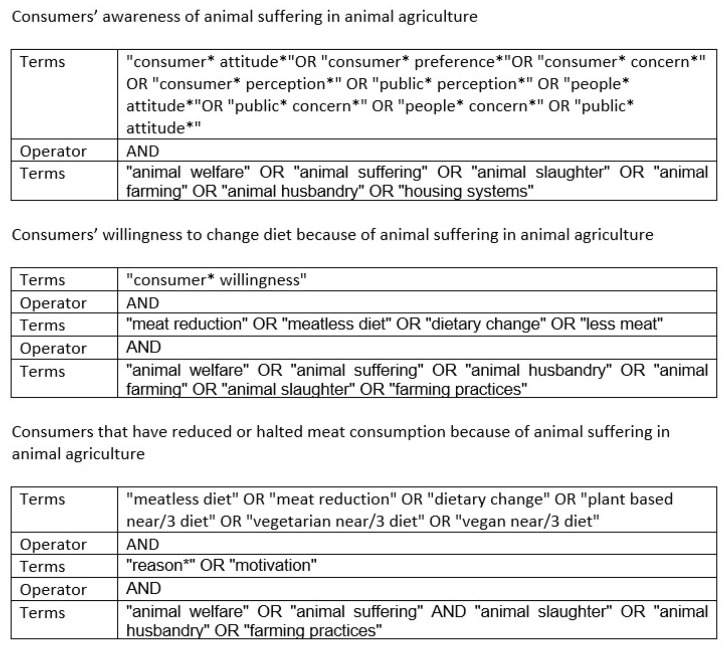
Search strings.

This systematic review followed the PRISMA 2009 (Preferred Reporting Items for Systematic Reviews and Meta-Analyses) protocol [85]. Based on previous scientific knowledge on the topic of animal welfare and after conducting preliminary searches on Google Scholar, we identified a series of keywords to search for literature on consumers’ attitudes towards animal welfare or suffering in the context of animal agriculture. We conducted three queries in the Web of Science (WOS) Core Collection in May 2022. The first aimed to find studies on people’s awareness and other attitudes regarding animal farming practices, animal characteristics, animal transportation, and slaughter; the second looked for papers reporting consumers’ willingness to reduce meat consumption because of animal suffering; and the third searched for literature on actual dietary change motivated by animal welfare. Search strings for each query can be found in Figure 1.

The screening process was completed by both authors independently to reduce bias. It comprised three stages for each of the three stages considered. First, titles, abstracts, and keywords were screened. Relevant articles on either awareness, willingness, and/or change were imported to Mendeley reference manager. Second, selected citations were read in full to make a final decision on their relevance for any of the three topics considered, and to locate new relevant articles that had not been found by the WOS search. Third, these first two steps were repeated until citation redundancy was achieved. When a reference was declared eligible by one reviewer only, the two reviewers reached an agreement on its inclusion or exclusion. Flow charts of this process can be found in Appendix A. Pertinent data from eligible papers were abstracted in tables (numbers of tables) with categories including study design, sample characteristics, question or dependent variable and covariates effects, among other relevant information.

**Figure 2:**
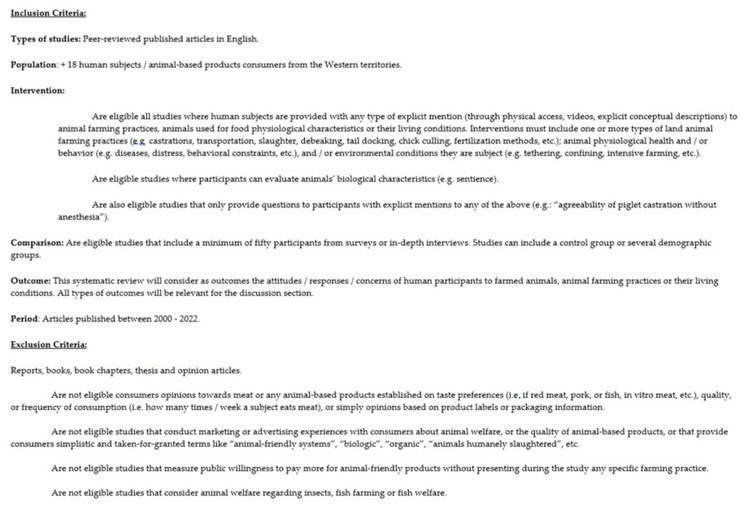
Eligibility criteria.

The eligibility criteria for this systematic review appears in detail in Figure 2. The principles that support our eligibility criteria are the following: (1) Studies must either provide participants, prior to experiments, with explicit information (pictures, videos or written information) about animals’ living conditions, the practices to which they are subject, their characteristics and their suffering; or (2) Studies pose questions to find out consumers’ awareness of, or agreeability to, animals’ sentience (animals’ biological and psychological characteristics), their living conditions, or farming practices. The reason for (1) is that only by providing reliable information about animals’ characteristics, their living conditions, and the practices to which they are subject can consumers truly understand the object of inquiry, i.e., animal suffering/welfare. The reason for (2) is that only by knowing consumers’ awareness of animal suffering and/or their understanding of animal biological and psychological characteristics is it possible to evaluate consumers’ attitudes and behavior towards reducing meat intake. In other words, attitudes in general, and willingness to limit meat consumption and actual dietary change, in particular, may depend on the type of information (vague and general vs. concrete and explicit) to which consumers are exposed.

Given that standardized patterns of animal production (e.g., similar techniques in animal husbandry, animal breeding, nutrition), productivity criteria (e.g., number of animals raised per square meter, animal weight at slaughter) [86], and cultural conventions in human–animal relations (e.g., eating cows and caring for dogs) are uniform in the Western globalized world, this systematic review is limited to these respective territories.

## 3. Results

Consumers’ awareness, willingness and dietary change regarding animals and animal agriculture have been studied with participants from several countries in North and South America (Canada [86,87,88], U.S. [86,87,89,90,91,92,93,94,95,96,97,98,99,100,101,102,103,104,105], Mexico [106,107], Argentina [108], Bolivia [108], Brazil [71,72,109,110,111,112,113,114,115], Chile [108,116], Colombia [108], Ecuador [108], Peru [108]), Western and Eastern Europe (Belgium [109,117,118,119,120], Denmark [117], Finland [121,122,123], France [115,119,124], Germany [117,119,123,125,126,127,128,129,130,131,132,133,134,135,136], Italy [119], Ireland [137,138], Netherlands [75,119,133,139,140,141,142,143], Norway [119,138,144,145], Portugal [119], Spain [106,123], Sweden [119], Switzerland [119], U.K. [98,146,147,148,149,150], Scotland [137], Bosnia [79,151], Bulgaria [79,119], Croatia [79,119,151], Czech Republic [79,119,138,151], Hungary [151], Macedonia [79,138,151], Poland [79,117,119,123,151], Slovakia [79,151], Slovenia [119,151] and Ukraine [79,119,151]), and Australia [72,152,153,154,155,156,157,158,159,160,161,162].

The following Sections summarize the results from 79 articles that examined consumers’ awareness of animal suffering (Section 3.1, Section 3.2, Section 3.3, Section 3.4, Section 3.5, Section 3.6 and Section 3.7, Table S1 in Supplementary Materials); 13 articles that present results about consumers’ willingness to change their diets due to animals and animal agriculture (Section 3.8, Section 3.9 and Section 3.10, Table S2 in Supplementary Materials); and 3 articles about consumers’ dietary changes due to animal suffering (Section 3.11, Table S3 in Supplementary Materials).

### 3.1. Consumers’ Awareness of Animal Suffering in Animal Agriculture

Different studies directly address participants’ different degrees of awareness about animal production systems, practices, and animal suffering. Brazilian consumers considered themselves somewhat informed (34%) or intermediately informed (20%) about animal agriculture [111]. Concerning the beef and dairy sector, only 21% are aware of culling of new-born male calves [72]. Still from Brazil, (33%) consumers reported awareness about cow–calf separation, and 15% about dehorning without pain control [72]. In Australia, participants reported high levels of awareness of caged-egg production [152], which seems to contrast with two other studies where 55% of Dutch participants seemed to be aware about the culling of one-day-old chicks [56,57]. Concerning confined systems, 83% of Chilean participants reported having low information about animals’ restriction of movement [163]. Most respondents (51.6–88.5%) from Finland, Germany, Poland, Spain, and the U.K. were unaware of production diseases in factory farming [123]. In two studies from Brazil, awareness of zero grazing ranged between 31–32% [71,72]. In the US, participants (53.6%) stated they were unaware of tie stalls for dairy cows [89]. In another study, 51.32% of Brazilian participants reported awareness of the farrowing housing system [114]. Concerning piglet castrations, there is a significant amount of discrepancy in the awareness among consumers: Brazilians (30–33%) [164] and (58.8%) [163]; Belgians (45.9%) and (50%) [165,166]; French, Germans and Dutch (48.5%) [133]; Germans (60%) [145] and between 17–73% [125]; Norwegians (60%) [119,144,145]; and Chileans (79%) [163].

### 3.2. Consumers’ Attitudes towards Animal Farming Practices in General

With one single exception of a study where 70% of Australian consumers remained unconcerned about farm animal welfare [155], according to the published literature most consumers do have negative attitudes, concerns, and trust problems towards animal farming practices. Among Finnish participants, older people expressed more trust in prevailing animal production, whereas women, urban residents, and people with companion animals expressed less trust in prevalent farming [122]. Brazilian participants who reported a higher level of knowledge about poultry and dairy supply chains were more likely to perceive the general conditions of animal welfare as being bad compared to those participants who reported a lower level of knowledge [110]. In a cross-cultural survey about how meat consumers perceive farm animal welfare, Spanish and Mexican women scored higher than men; urban consumers scored higher than rural ones; more educated Mexicans were more concerned about animal welfare; the oldest consumers in both countries assigned a lower grade to the importance of animal welfare [106].

Brazilian and French participants (31.3%) cited freedom from discomfort as the most important issue contributing to good animal welfare; 61.3% strongly agreed that sheep raised under intensive management systems experience low levels of welfare; older participants and those with a lower education seemed to view animal welfare mainly in terms of physical health [115]. Without accessing any information during the survey, high percentages of Brazilian lay participants had no hesitation to qualify the following practices as “animal maltreatment”: “an animal socially isolated and with no contact with other animals” (93.2%), “to dock the tail without anesthetic” (80.9%), and “an animal in a location with movement restriction” (68.5%) [113]. Results from a study about views on pig farms in the U.S. showed 74% of the respondents addressed concerns relating to animal welfare, particularly space to move, feeding, contact with outdoors or nature and absence of pain, suffering and mistreatment [104]. In an exploratory study, the type of information provided affected the percentage of Brazilian lay participants who rejected zero-grazing systems for dairy cows or early cow–calf separation: (information capsule) zero-grazing (86.1%); cow–calf separation (69.2%) vs. (Short statement) zero-grazing (75.7%); cow–calf separation (61.7%) [112]. Canadian and U.S. participants (44%) (mostly males) supported early cow–calf separation, and 48% were opposed to it [87]. The most concerned Dutch participants were worried about the following parameters in sow husbandry: fear/anxiety and pain, number of animals per m^2^, floor cover, and tail docking [143]. The results of an online survey suggest that German participants (especially women) negatively assessed the most conventional production methods (e.g., foie gras; lobster boiled alive; bull beef from intensive production; veal from conventional production; beef from cattle in tie stalls; meat from broilers from intensive production systems; pork meat from pigs from intensive commercial units) [134].

### 3.3. Consumer Attitudes towards Intensive Housing Systems

Consumers tend to associate confinement and intensive farming with animal suffering [88,100,132]. For instance, 79% of Brazilian participants considered farmed animals in production systems as not being well treated due to restriction of movements, and for 39% farm animal welfare was a major concern [111]. The more intensive the housing, and limited the space for animals to move, the more respondents disapproved of indoor pens for lambs, particularly women (78%), and urban and suburban (49%) residents [159]. A qualitative study with a focus group showed that in all discussions with German participants the topic “factory farming” was discussed critically, in particular the lack of free movement, too little space per animal, and non-transparent, locked systems, especially in pig and poultry production [136]. When asked about their opinions about animal production for laying hens, broilers, and pigs, respondents in Germany rated these intensive systems more unfavorably than respondents from other countries (Finland, Poland, Spain and the U.K.) [123]. Overall, Dutch consumers perceived conventional broiler systems to be less animal friendly than organic broiler production systems [142], and Germans considered it unjust to keep egg-laying hens in battery cages [167]. Australian participants saw confinement in caged-egg production as an unnatural form that restricts animals’ natural behaviors [152]. Most Belgian consumers considered that broiler chickens suffer [109].

In pig production, 69% of U.S. consumers surveyed would support a ban on gestation crates [168]. A study with U.K. participants revealed that consumers who show the highest levels of concern are more likely to be aware of modern pork production methods [147]. Europeans scored the lowest evaluations of “slatted floor” pig production systems and stocking density [117]. In another study, German opponents to intensive housing systems (22%), particularly younger people and those with better knowledge, expressed a very low acceptance of intensive pig production systems [131].

Consumers’ attitudes to intensive farming systems tend to be more negative when they are provided with additional information. For instance, the number of Canadian and U.S. supportive participants (30.4%) of gestation stalls for pregnant sows dropped to 17.8% after being provided with additional information (younger participants with higher education and women were less supportive of gestation stalls) [86]. When Brazilian participants (N = 173) were shown a video with information about the suffering of sows, a vast majority rejected it (87.3%) [169]. After being provided with images, German consumers (25.9% slightly/25.9% very) considered fully indoor housing systems as cruel for cows [132]. Likewise, after accessing images of different housing systems (farrowing crates, loose pens, and outdoors) during the experiment, Brazilian participants expressed negative attitudes, especially towards farrowing crates [71]. In two different studies about dairy production systems, after observing four sets of pictures of each husbandry system type for dairy cows, the negative evaluation of indoor housing by German participants systems was more than 50% [132]. Even if participants were provided with information (i.e., pros and cons for animals and production systems) in regards to cattle fattening, the opponents (59.3%) were still in the majority compared to other groups (i.e., the indifferent 20.5%, and the supportive 20.2%) [163].

### 3.4. Consumers’ Beliefs about Farmed Animals’ Characteristics

The published literature indicates that consumers usually recognize animals (in general) as sentient. When provided with a definition of grief, 96% of Australians, especially those who had pets, were older, and those who lived in rural locations, agreed that animals can experience grief, fear (99%), happiness (96%), distress (95%), and sadness (92%) [158]. Consumers also tend to acknowledge farmed animals (e.g., pigs) experience emotions, pain, boredom, fear and stress [75,130,146]. However, evidence also indicates that consumers tend to hierarchize animals’ sentience by species. In a study that involved European students, the attributed sentience for different species ranked from highest to lowest: chimpanzee > dog > dolphin > cat > horse > cattle > pig [138]. Similarly, in another study, participants from four countries in the U.K. ranked pets and farmed animals’ capacity to experience sensations as follows: pain—dogs (91.4%) > cats (87.6%) > pigs (81.0%) > cows (85.5%); fear—dogs (87.1%) > cats (79.5%) > pigs (76.6%) > cows (75.6%) [137]. The results from another study with Australian participants ranked animals’ capacity to experience grief as follows: dogs (98%) > chimpanzees (97%) > dolphins (94%) > cats (88%) were better ranked compared to > pigs (73%) and > cows (71%) [154]. Finnish participants, especially those who were younger and the ones who have companion animals, believed that dogs (97.3%) possess more mental capacities than > cows (96%) > pigs (95%) or > chickens (91.4%) [121]. Participants from Argentina, Chile, Colombia, Ecuador, Peru, Bolivia, and Mexico believed that farmed animals feel pain, and are able to feel positive or negative emotions [107,108]. When asked to evaluate sheep sentience, (75%) French and Brazilian consumers (with higher scores for women, older people, and Brazilians) considered sheep as capable of feeling fear, happiness and suffering [115].

The conventions about the presentation of animal foods (i.e., food vocabulary, and removing of animals’ individual characteristics, i.e., the head of an animal) facilitates dissociation and reduces consumers’ ability to reflect upon the animal [170]. Associating meat with animals might lead to lowering beliefs about animals’ minds (i.e., animals’ capacity to feel pain, hunger, pleasure, fear, happiness, etc.), consequently enabling meat eating [101,102,171]. In a study (1) where U.K. participants were provided with descriptions of pigs, cows, and chickens as sentient beings, those who were more committed to eating meat were more likely to want to avoid information about food animals’ sentience [98]. Consumers attributed less sentience to cows in an experiment that showed a graphic link between meat and its animal origin in the pasture. [124]. Overall, U.S. consumers expressed more disgust towards meat sourced from a baby animal than the same meat sourced from an adult animal [99]. In another study, although Australian women meat eaters experienced greater concern for animals, when exposed to audio-visual footage demonstrating the intelligence of a lamb, most participants expressed defensive justifications for eating meat [156]. Nonetheless, results from a study that contradicts the previous ones showed that participants exposed to beef attributed significantly less capacity for sensation to cows than participants exposed to a living cow [172].

Evidence also shows that participants with the most prominent ethical values towards farmed animals tend to level humans and other animals. When asked to measure mind attribution to animals while viewing a picture of a cow, Canadian participants who wrote “animals are human-like” indicated more inclusive attitudes to other animals [157]. Finally, in an online experiment that provided videos on housing systems for pigs and asked about their ability to feel emotions, more ethically minded German participants (Cluster 3) were more agreeable [128].

### 3.5. Consumers’ Attitudes towards Mutilations in Animal Husbandry

The information (or its absence) provided during experiments about different types of mutilations (with or without anesthesia) in animal husbandry seems to be a fundamental factor of influence in consumers’ attitudes, especially if the information mentions animal suffering. For Eastern European consumers that did not access any information during the experiment, the overall opinion about surgical castration tended to be positive [79]. When asked about surgical interventions in pig husbandry, without being provided with information about animal suffering, (German) participants tended to be more agreeable about tail docking, teeth grinding, and castration [126]. Similarly, castration and tail docking of piglets are issues that need additional care only for a few Dutch respondents (27.4%) [141]. After receiving basic information, at least 61% of European consumers (Belgians 87%, Swedish 80%, Germans 76%, Italians 74%, and French 74%), and especially men, considered piglet castration without anesthesia unacceptable. [119]. In a focus group study, statements used to inform consumers about current piglet castration practice resulted in 77% of participants finding surgical castration without anesthesia unacceptable [145]. After being informed about the procedures, most Brazilian participants (especially in Survey 1–73%) were opposed to piglets’ surgical castration without pain control [164]. During a focus group, several (German) pork organic consumers expressed surprise and disappointment with reference to organic farming, when they were informed about castration without anesthesia [125]. After being given information about the surgical castration without anesthesia of male cattle, pointing to pros and cons for animals and production systems, 62.5% of Chilean participants were opposed to this practice [163]. In an online survey after Norwegian participants were confronted with information concerning piglets castration methods, surgical castration without anesthesia was considered unacceptable to 70% (especially by women); with additional information, the unacceptability rate for castration without anesthesia rose to 77% [144]. Likewise, after being provided with the explanation, the banning of unanesthetized castration was voted for more by women (75.4%) than men (68.7%), those with higher education (high school 67.4%; 75.3%; university 73.6%), and consumers who ate less pork (*p* = 0.0208) [165]. Results from an online survey with Brazilian and French consumers showed that women attributed higher scores of suffering to sheep during tail docking and reproductive techniques [115]. When provided with information, U.K. consumers largely considered mutilations (tail docking, teeth-clipping, nose-ringing of pigs; debeaking hens, debeaking, de-toeing and desnooding turkeys; different methods of lamb castration and tail-docking; different methods of cattle castration and nose ringing, as well as the dehorning and disbudding of cattle and goats) as unnecessary for animals [148].

Regarding animal suffering, consumers seem to perceive different methods of mutilation on distinct species contrastingly. Results from an online survey suggest that U.S. residents consider tail docking and dehorning more detrimental to dairy cattle than other practices [103]. When exposed to a detailed explanation of different mutilation procedures, U.K. participants perceived surgical castration in calves and kids up to 2 months without anesthetic as the most painful procedure, followed by surgical castration in lambs, and crushing of the spermatic cord in calves and kids without anesthetic [148].

If presented with other less painful procedures on animals, consumers tend to choose alternatives to mutilations: Belgian, French, Dutch, and German participants have a preference for the piglet vaccine method in place of physical castration [133]. In another study with Belgian respondents, 60% evaluated immunocastration slightly better than surgical castration [166].

### 3.6. Consumers’ Attitudes towards Livestock Transportation

A study that sought to ascertain the responses of the public who had encountered the media coverage of cruelty towards Australian cattle being exported found the most common emotional reactions were pity for the cattle 85%; sadness 72%; anger 68%; and from other responses (27%) 33% felt disgust and 12% felt sick [160]. The study found that women were more likely to feel sad and angry, to look away or stop listening to the media coverage, to perform any action or to discuss the media coverage with others, and those with a higher level of education were more likely to sign a petition to ban live exports [160]. In another study about sheep and cattle transportation, Australian consumers found that animals are “crammed” or “shoved” into trucks with limited space and that this was “bad treatment”, but participants expressed more “disgust” and “sadness for animal cruelty” during long distances at sea [153]. Observing transportation of animals to the slaughterhouse might trigger the animal-meat connection [170]. However, in another study (focus group), when Australian meat consumers were provided with content from animal welfare activism, most ignored it or considered it extreme or unreliable (dissonance) [153]. Furthermore, in Australia, a study revealed that despite a wide media coverage exposing animal cruelty in live export of sheep by sea, respondents’ underlying attitudes and beliefs were not affected [162]. An exploratory survey shows that Eastern European consumers are not clear about whether the animals consumed are transported incorrectly (women, older people, and Bulgarians agree more with the statement that the transportation of animals is inadequate) [79].

### 3.7. Consumers’ Attitudes towards Animal Slaughtering

There is still a paucity of research in the literature about consumers’ attitudes towards animal slaughter. In a study with U.K. halal meat consumers, 69% disagree that stunning meat animals prior to slaughter reduces the pain associated with slaughter, and 69.9% (men 70.7%/women 67.9%) prefer slaughter without stunning [149]. In another similar study with halal scholars and consumers from the U.K., 78% believe that if an animal is slaughtered without any form of stunning, the animal will feel reduced pain because the knife acts as a stun [149,150]. Religion seems to be a predominant factor for (not) purchasing halal meat [161]. In a study that did not provide any prior information about negative impacts of culling chicks, 30% of (Dutch) participants consider it to be a good practice or do not have problems with it [140]. In another study with Dutch participants provided with information about culling day-old chicks at different stages of the questionnaire, 78.8% (especially highly educated women) expressed disagreement [139]. Disagreement is even higher in Brazilian consumers (90%) when asked about culling newborn male calves [72].

Without being provided with any information during an exploratory survey, most Eastern European consumers (mainly women, consumers from urban areas, Slovenians followed by Bulgarians, North Macedonians, Romanians, and Ukrainians) agreed that slaughter systems should be improved [79]. An experimental study, where Belgian participants were shown a short 360° documentary depicting what pigs experience inside a slaughterhouse, provided mixed evidence: on the one hand, the footage had a positive effect on empathic concern, on the other hand, it might negatively affect empathic concern by evoking more speciesist (discriminatory) attitudes [120].

### 3.8. Consumers’ Willingness to Change due to Animal Farming Practices in General

Providing information about animal suffering during the experiments can influence consumers’ willingness to reduce meat intake. For example, after German participants (N = 590) were provided with “newspaper articles” describing the negative effects of meat consumption on animals, 150 participants (82 women) believed they would reduce meat consumption in the future [129]. In another study, U.S. participants expressed greater intentions to reduce meat consumption based on animal welfare compared to others based on the environment and health issues, and control group [94]. During an experiment, consumers that read a text campaign about animal farming conditions expressed more meat aversion and willingness to eat vegetables comparing to other participants that accessed messages related to health and environment [97]. Inversely, presenting to participants animal production practices that cause animal suffering might also trigger meat-eating justification strategies to the detriment of the willingness to eat less meat. In another study, even though German participants provided the most negative attitudes towards intensive meat production systems, major findings indicate a very limited effect on meat consumption [134].

### 3.9. Consumers’ Willingness to Change: Meat–Animal Association

Dissociating meat from animals reduces empathy, disgust, and thereby increases the willingness to eat meat. The opposite phenomenon also occurs, where meat–animal association might result in consumers’ willingness to eat less meat. In an experiment where Norwegian participants were divided into five different studies with animals presented at different processing stages, the results suggested that processed meat made participants less empathetic towards the slaughtered animal than unprocessed meat; conversely, presenting the living animal, and changing the vocabulary (e.g., replacing “beef/pork” with “cow/pig”) increased empathy and disgust, and reduced willingness to eat meat and increased willingness to choose vegetarian options [96]. When U.S. participants were provided with photographs of meat and animals (see experiment in Table S2), meat was least appetizing when it was presented along with an image of a baby animal, and most appetizing when it was presented without any image of the animal source [99]. In another similar experiment, participants that access meat dishes paired with live animals (animal–meat association) presented greater disgust for meat, and reduced antiveg*n attitudes via increased empathy for animals, compared to other participants that accessed images of meat (meat-alone condition) [90]. Another study revealed that local cultural conventions (i.e., the way animals are cooked) play a major role in meat–animal dissociation/association and might trigger meat disgust. After being shown a roasted pig (a) with a head and (b) without a head, for both Ecuadorian and especially U.S. participants, presenting the head decreased dissociation, led to more empathy and disgust and a higher willingness to choose a vegetarian option [91].

In an experiment with U.S. and Norwegian participants that manipulated the cuteness of a presented animal, evidence supports increased cuteness resulting in less willingness to eat meat, and increased empathy towards the animal [95]. Anthropomorphizing animals used for food can also increase consumers’ empathy and enhance willingness to eat less meat. U.S. participants (especially women), to whom it was proposed to anthropomorphize a pig and a cow, expressed more willingness to adopt a meat-free diet (study 2) [93].

### 3.10. Consumers’ Willingness to Change due to Animal Slaughter

With a focus on slaughtering an animal to produce meat (study 1), French participants reported lower willingness to eat beef in an experimental condition that emphasized the slaughter of a cow compared to a condition that presented a diagram of a cow as meat [124]. During an experiment, a 360° VR video format depicting the death of pigs in a slaughterhouse (see Table S2 of the article for footage details) increased Belgian participants’ empathetic concern for the animals, increased their intentions to reduce animal food intake, but, on the other hand, evoked speciesist attitudes [120].

### 3.11. Consumers’ Dietary Change

Overall results from an experiment that tried to induce effects on meat reduction with students (treatment group) through an animal-advocacy pamphlet that described the impact of factory farming on animals and the health benefits of eating a plant-based diet, suggested a decline in consumption of meat, poultry, and fish consumption for men, and substitution of red meat (beef) to poultry/fish for women [92]. With the same objective of lowering red and processed meat consumption, persuasive arguments about health impacts were more efficient at reducing red meat consumption than other arguments (environmental and animals) [173]. Data from another study support that higher levels of animal anthropomorphism predict greater empathetic concern for animals and their suffering: 24.5% of participants who reported they had reduced their meat consumption had done so for less than a year [174].

## 4. Discussion

Consumers reported low awareness about animals’ restriction of movement [71,72,89,163], dehorning without pain control, culling calf males, cow–calf separation [72], diseases in factory farming [123], expressed different degrees of awareness about piglet castrations [119,125,133,144,145,163,164,165,166], and caged-egg production [56,57,152]. There is strong evidence among scholarly research that consumers believe that animal welfare should be improved [38,75,114,139]. In particular, confinement, restriction of movements [71,111,112,123,132,134,135,136,143,152,159,167,169], tail docking without anesthetic use, and animal isolation from other animals [112,113,143] raise concerns and negative attitudes among consumers, mostly women, younger consumers, urban consumers, higher-educated consumers, consumers with more knowledge, and consumers who have pets [106,110,122,165]. Results show a high degree of disagreeability towards mutilations without anesthesia (e.g., surgical castration, tail docking, teeth grinding) [119,125,144,145,148,163,164]. On the other hand, mutilations with anesthesia [79,126,141] and other alternatives (e.g., immunocastration) [133,166] are methods gaining support among consumers. Consumers’ attitudes towards livestock transportation are also negative [79,160], and more negative towards sea transportation [160,170]. The type of information (or its absence) provided during experiments is an essential factor of influence on consumers’ attitudes towards animal suffering. The provision of information increased the percentage of participants’ criticism about current farming practices [86,112]. Concerning information medium, video footage [169] and pictures of indoor housing systems [71,132,135] seem to be more efficient than written information [163] at triggering more criticism from consumers. Negative attitudes and/or dissonant attitudes were prompted by video footage of animal transportation by sea [170]. It is worthwhile for future interventions to invest in triggering emotions (disgust), while having an educational component, providing footage about current animal farming practices (e.g., sows in gestation crates, laying hens in cages, separation of cows and calves, sows and piglets, mutilations without anesthetic, genetically manipulated animals, etc.) with audio or written educational information about the negative consequences (pain, physiological stress, abnormal behaviors, several health issues, and even premature death) for animals. Furthermore, animal–meat dissociation can be disrupted with the individuation of animals used for food (by providing names to specific non-human individuals) while displaying the practices to which they are subject [175]. Combining the previous methods of information with happy animals being cared for in sanctuaries, emphasizing human–animal positive bonding, framing their intrinsic value, can trigger empathetic emotions.

Few studies have reported most halal Islamic meat consumers believe slaughtering of animals should be performed without stunning, because the knife acts as a stun [149,150]. To our knowledge, little is known about what Western (non-Islamic) consumers think about halal slaughtering methods being carried out in the Western world, or even about the conventional methods (e.g., routing, stunning, hanging, bleeding) used in slaughterhouses in Western countries. Consumers, especially women, reported disagreement towards culling day-old chicks or male calves [72]. A study where participants watched footage of what pigs experience in a slaughterhouse triggered empathy and also discriminatory attitudes [120]. Future research could study potential avoidance, dissonance, and dissociation mechanisms with slaughtering footage and images of animal-based products (i.e., if consumers who strongly disagree with calves being killed consume veal meat, or drink cow’s milk).

Although consumers acknowledge animals’ sentience [75,107,108,115,128,130,146,158], our systematic review shows strong evidence that species used for food are ranked below pets and, in some cases, wild animals [121,137,138,154]. A major factor in attributing fewer sentience capacities to farmed animals can be the (physical and emotional) distance between the consumed species and consumers (especially in urban contexts), and vice-versa (the physical and emotional proximity between many consumers and pets). A second factor is the reproduced anthropocentric and utilitarian framings of other animals as food resources (meat and analogs) [176,177], which facilitates animal–meat dissociation [101,102,124] and reduces the ability to reflect upon the animal [170,172]. Devaluation of animal sentience is more evident in men with a lower level of education, the elderly, those from rural regions [178], and less ethically minded participants [128]. However, consumers from rural areas that raise and/or slaughter animals in small facilities do acknowledge their sentience (e.g., recognizing pigs as cognitively superior to dogs, or that animals cry before slaughter), and, in some cases, emotional bonds can be developed among these individuals and other species that might acquire temporary or, in rare situations, permanent pet status [175]. Therefore, future research to study the contradictory attitudes of this demographic group of consumers/breeders towards other species used for food would be interesting.

Few published studies that reported consumers’ willingness to change their diet cited animal suffering as a more compelling motive than health or environmental reasons [94,97,129]. However, animal suffering can also trigger meat-eating justification strategies, and prompt a very limited effect on meat consumption [120,134]. Mixed results (empathy towards animals, intentions to reduce meat vs. speciesist attitudes) were found in consumers that access footage about pigs suffering in factory farming and slaughterhouse [120]. A photo of a cow with a short statement that she will be sent to an abattoir found a reduced willingness to mind attribution [124]. Footage or descriptions demonstrating the intelligence and sentience of animals find defensive justifications for meat eating [156], or information avoidance (from participants more committed to eating meat) [98]. Most people eat meat but disapprove of animal suffering. The “meat paradox” [172] implies animal-based product intake is also linked to some strategies that make it possible to reduce cognitive dissonance, such as dissociating meat from its origin, or the denial of animal cognition [171] and their ability to feel pain [179]. Few studies gather opinions from consumers about the prevailing production processes combined with animal sentience and meat association [77,180,181]. Current research also supports that one of the most expected attitudes is indifference, the dissociation of the product from the animal, or even the avoidance of thinking about the upsetting production processes involved [68,80].

Demographic factors, such as gender, race, ethnicity, place of residence, social class [182], socioeconomic position [183], age, education [184], and political orientation [178], seem to affect dietary choices, particularly meat consumption. Other factors, such as taste preferences, culinary traditions, social norms [185], hedonism, affinity, and privilege [186], also play a major role in meat intake still occupying a central role in contemporary diets [187]. Western cultural and educational discourses play a structurally major role in reproducing the invisibility of animal suffering, in reinforcing anthropocentric dualisms (e.g., human/animal; animal/meat), and therefore legitimizing instrumentalized and utilitarian views of factory-farmed animals [176,177,188]. Furthermore, neoliberal policies continue to value economic development [189], framing animals as assets and food resources, to the detriment of sentient individuals.

Along with other scientific research published in the literature [190,191], this systematic review reveals that gender is the most consistent (and predictable) demographic factor that influences some discordant views about animals and meat. In regard to live animals, with one single exception [110], our systematic review reveals that female consumers express greater concerns for animal welfare [72,106,115,122,134,143]. They tend to perceive more negatively intensive housing systems [136,159], stocking density, pen size, and group size in farm animal production [118]. Women are also less supportive of cow–calf separation [87], gestation stalls for sows [86,114,169], are more supportive of the ban on tie stalls for cows [89], and present higher disagreeability rates about animal sea transportation [151,160]. Inversely, male respondents agreed more with the practice of castration without anesthesia [119,144,151], with other types of mutilations (e.g., debeaking, tail-docking, dehorning, etc.) [115,139,148], and are less supportive of the banning of castration without anesthesia [165]. Females are more likely to assert that animals could grieve [154] or experience pain and boredom [137]. However, when asked about halal slaughter, female halal consumers presented similar acceptability rates compared to males [149]. In regard to diets, and more than men, women are more motivated to reduce meat consumption due to animal welfare [129,156]. Women also indicate decreased meat attachment and more willingness to adopt meat-free diets [93]. However, although women are more willing to reduce beef consumption because of meat–animal association, they can also increase their consumption of poultry and fish [92].

Our systematic review reveals that appeals towards animal welfare and meatless diets are more effective on women than men. Several conventional masculinity traits (i.e.,: strength, athleticism) are traditionally connected to meat consumption [192,193]. In this sense, especially males that are more compromised with masculine traditional attributes use different strategies (e.g.,: denying animal suffering, low hierarchizing other species, believing animals are meant to be converted to food, etc.) as forms to justify meat eating [194]. Evidence also suggests that individuals who avoid meat, especially vegans, are stigmatized by veganphobes [195] for disrupting social conventions related to food [196]. Additionally, veg*n options can be deemed to be more “feminine diets”, as they are deficient in proteins, [187], and therefore not suitable for males. Because meat eating is linked to masculinity, a limitation of the current literature that future research can explore are appeals with consumer participants where plant-based meals are paired with veg*an healthy and strong (fe)male athletes, and/or paired with additional information that emphasizes the nutritional quality of plant-based food.

This systematic review shows that the more efficient strategies that trigger meat–animal associations, empathy that increases disgust, and thereby increases willingness to eat less meat and choose vegetarian options, seem to come from experiments where participants are presented with living animals, or living animals paired with meat, where their individual features are emphasized (e.g., cuteness, babyness), shifting speciesist vocabulary (e.g., replacing “beef/pork” with “cow/pig”). Furthermore, anthropomorphizing [90,91,92,93,96,99,174] seems to predict greater empathetic concern for animals and induce dietary change in consumers who choose to reduce meat consumption, and an increased willingness to choose vegetarian options. Positive results were found in experiments that tried to induce meat reduction through an animal-advocacy pamphlet appealing to animal suffering along with information about the health benefits of plant-based diets [92]. Persuasive arguments about health were more persuasive than others (environmental and animals) essentially for red meat reduction [173]. Interventions that contained text, photographs, infographics or videos seem to have meaningful effects of primarily self-reported outcomes [36]. Mass media coverage on animal suffering [129,160] seems to induce more favorable reactions among consumers, whereas animal activism campaigns lead more participants to ignore their content and find it unreliable [97,153]. Evidence also shows that the most concerned consumers and the ones with higher ethical values who tend to level humans with other animals are capable of displaying more inclusive attitudes [128,143,157]. For future research, we highly recommend the following combined interventions (highlighted by Kwasny et al., 2022): informing consumers about the negative impacts of meat combining health and environmental appeals; communicating emotionally framed messages (related to animal suffering, showing pictures of unprocessed meat, pictures of living animals or cute animals in restaurant contexts); providing competence training and supporting habit change (counseling with educational materials on healthy lifestyles, providing cooking courses to aid in the preparation of vegetarian food); increasing the visibility of vegetarian food (e.g., by labeling a vegetarian meal as “dish of the day”) [197].

An intrinsic limitation of most experiments cited above are their effects only in a short term. Cultural traditions, rooted habits, and convenience related to the intake of meat and other animal-based products play a major role in dietary change. Therefore, longitudinal interventions where subjects are continually provided with counterhegemonic information can enable dietary changes in the long term.

## 5. Conclusions

This systematic review reveals that consumers have low awareness of animal agriculture. The provision of information during experiments shows clear evidence that consumers increased levels of criticism towards animal suffering due to animal husbandry, especially confinement, restriction of movements, mutilations without anesthetic, and sea transportation. Criticism is higher among women, younger, urban, and more highly educated consumers. The reviewed evidence shows that interventions appealing to animal suffering due to farming practices, transportation, slaughter, and animals’ characteristics are sufficiently appealing to influence consumers’ willingness to reduce meat consumption, and even dietary change in the short term. It also seems that animal suffering is a more compelling motivation for consumers’ willingness to change their diet compared to for health or environmental reasons.

Hegemonic sociocultural factors related to the strong attachment to meat can complicate changes in diets. Furthermore, the reproduced anthropocentric and utilitarian framings of other animals as food resources facilitates animal–meat dissociation, meat-eating justification strategies, indifference, and reduces the ability to reflect upon the animals. It is up to all stakeholders (policymakers, educators, scientists, and the media) to foster more critically informed citizenship, and establish fewer instrumental views of non-human animals, alternatively addressing their intrinsic value, while linking current animal agriculture to unsustainable food systems, human health, environmental degradation, and animal suffering. In addition, it is important to encourage plant-based diets as more ethically responsible eating practices, with lower environmental impacts, and with evident human health benefits.

## Figures and Tables

**Figure A1:**
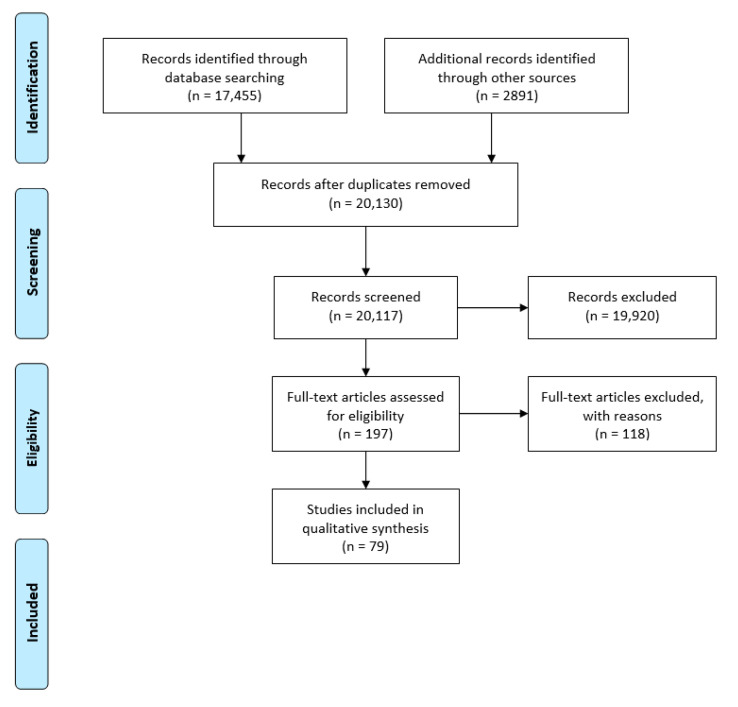
PRISMA 2009 Flow Diagram—Awareness [85]. For more information, visit www.prisma-statement.org.

**Figure A2:**
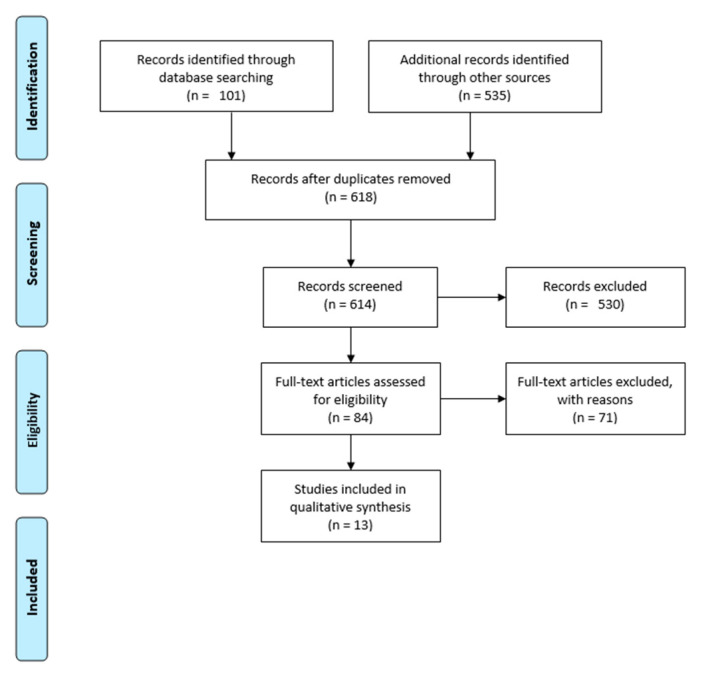
PRISMA 2009 Flow Diagram—Willingness [85]. For more information, visit www.prisma-statement.org.

**Figure A3:**
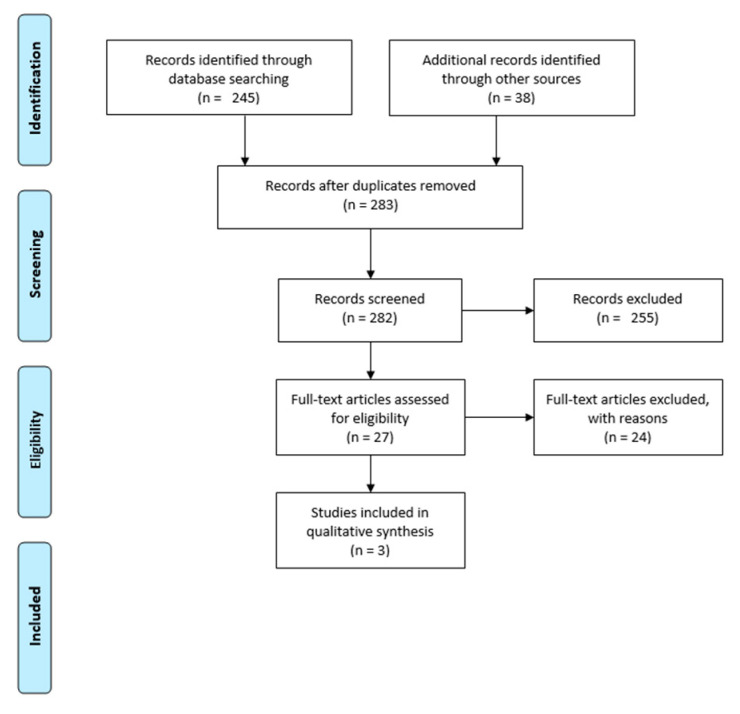
PRISMA 2009 Flow Diagram—Change [85]. For more information, visit www.prisma-statement.org.

## Supplementary Materials

The following supporting information can be downloaded at: https://www.mdpi.com/article/10.3390/ijerph192316372/s1, Table S1. Consumers’ awareness of animal suffering in animal agriculture. Table S2. Consumers’ willingness to change diet because of animal suffering in animal agriculture. Table S3. Consumers that have reduced or halted meat consumption because of animal suffering in animal agriculture.

## References

[1] Steinfeld H. (2006). Food and Agriculture Organization of the United Nations. Livestock’s Long Shadow: Environmental Issues and Options.

[2] Slingo J.M., Challinor A.J., Hoskins B.J., Wheeler T.R. (2005). Introduction: Food crops in a changing climate. Philos. Trans. R. Soc. B Biol. Sci..

[3] Bouwman L., Goldewijk K.K., Van Der Hoek K.W., Beusen A.H.W., Van Vuuren D.P., Willems J., Rufino M.C., Stehfest E. (2013). Exploring global changes in nitrogen and phosphorus cycles in agriculture induced by livestock production over the 1900-2050 period. Proc. Natl. Acad. Sci. USA.

[4] Dauvergne P. (2008). Shadows of Consumption: Consequences for the Global Environment.

[5] Godfray H.C.J., Beddington J.R., Crute I.R., Haddad L., Lawrence D., Muir J.F., Pretty J., Robinson S., Thomas S.M., Toulmin C. (2010). Food security: The challenge of feeding 9 billion people. Science.

[6] Thornton P.K. (2010). Livestock production: Recent trends, future prospects. Philos. Trans. R. Soc. B Biol. Sci..

[7] McGuire S. (2016). World cancer report 2014. Geneva, Switzerland: World Health Organization, international agency for research on cancer, WHO Press, 2015. Adv. Nutr..

[8] Bouvard V., Loomis D., Guyton K.Z., Grosse Y., El Ghissassi F., Benbrahim-Tallaa L., Guha N., Mattock H., Straif K., International Agency for Research on Cancer Monograph Working Group (2015). Carcinogenicity of consumption of red and processed meat. Lancet Oncol..

[9] Aykan N.F. (2015). Red meat and colorectal cancer. Oncol. Rev..

[10] Händel M.N., Cardoso I., Rasmussen K.M., Rohde J.F., Jacobsen R., Nielsen S.M., Christensen R., Heitmann B.L. (2019). Processed meat intake and chronic disease morbidity and mortality: An overview of systematic reviews and meta-analyses. PLoS ONE.

[11] Schulze M.B., Martínez-González M.A., Fung T.T., Lichtenstein A.H., Forouhi N.G. (2018). food based dietary patterns and chronic disease prevention. BMJ.

[12] Zhong V.W., Van Horn L., Greenland P., Carnethon M.R., Ning H., Wilkins J.T., Lloyd-Jones D.M., Allen N.B. (2020). Associations of processed meat, unprocessed red meat, poultry, or fish intake with incident cardiovascular disease and all-cause mortality. JAMA Intern. Med..

[13] Wang X., Lin X., Ouyang Y.Y., Liu J., Zhao G., Pan A., Hu F.B. (2016). Red and processed meat consumption and mortality: Dose–response meta-analysis of prospective cohort studies. Public Health Nutr..

[14] Kim K., Hyeon J., Lee S.A., Kwon S.O., Lee H., Keum N.N., Lee J.K., Park S.M. (2017). Role of total, red, processed, and white meat consumption in stroke incidence and mortality: A systematic review and meta-analysis of prospective cohort studies. J. Am. Heart Assoc..

[15] Rouhani M.H., Salehi-Abargouei A., Surkan P.J., Azadbakht L. (2014). Is there a relationship between red or processed meat intake and obesity? A systematic review and meta-analysis of observational studies. Obes. Rev..

[16] Mbow H., Reisinger A., Canadell J., Ginevra P.O. (2017). An IPCC Special Report on Climate Change, Desertification, Land Degradation, Sustainable Land Management, Food Security, and Greenhouse Gas Fluxes in Terrestrial.

[17] Willett W., Rockström J., Loken B., Springmann M., Lang T., Vermeulen S., Garnett T., Tilman D., DeClerck F., Wood A. (2019). Food in the anthropocene: The EAT-Lancet Commission on healthy diets from sustainable food systems. Lancet.

[18] Kirbiš A., Lamot M., Javornik M. (2021). The role of education in sustainable dietary patterns in slovenia. Sustainability.

[19] Pohlmann A. (2021). Lowering barriers to plant-based diets: The effect of human and non-human animal self-similarity on meat avoidance intent and sensory food satisfaction. Food Qual. Prefer..

[20] Scarborough P., Appleby P.N., Mizdrak A., Briggs A.D.M., Travis R.C., Bradbury K.E., Key T.J. (2014). Dietary greenhouse gas emissions of meat-eaters, fish-eaters, vegetarians and vegans in the UK. Clim. Chang..

[21] Segovia-Siapco G., Sabaté J. (2018). Health and sustainability outcomes of vegetarian dietary patterns: A revisit of the EPIC-Oxford and the adventist health study-2 cohorts. Eur. J. Clin. Nutr..

[22] Ploll U., Petritz H., Societal T.S.-E.I. (2020). A social innovation perspective on dietary transitions: Diffusion of vegetarianism and veganism in Austria. Environ. Innov. Soc. Transit..

[23] Ruby M.B. (2012). Vegetarianism. A blossoming field of study. Appetite.

[24] Beardsworth A.D., Keil E.T. (1991). Vegetarianism, veganism and meat avoidance: Recent trends and findings. Br. Food J..

[25] Fox N., Ward K. (2008). Health, ethics and environment: A qualitative study of vegetarian motivations. Appetite.

[26] Hussar K.M., Harris P.L. (2010). Children who choose not to eat meat: A study of early moral decision-making. Soc. Dev..

[27] Jabs J., Devine C.M., Sobal J. (1998). Model of the process of adopting vegetarian diets: Health vegetarians and ethical vegetarians. J. Nutr. Educ. Behav..

[28] Neale R.J., Tilston C.H., Gregson K., Stagg T. (1993). Women vegetarians: Lifestyle considerations and attitudes to vegetarianism. Nutr. Food Sci..

[29] Malek L., Umberger W.J. (2021). How flexible are flexitarians? examining diversity in dietary patterns, motivations and future intentions. Clean. Responsible Consum..

[30] Dagevos H., Voordouw J. (2013). Sustainability and meat consumption: Is reduction realistic?. Sustain. Sci. Pract. Policy.

[31] Dagevos H. (2021). Finding flexitarians: Current studies on meat eaters and meat reducers. Trends Food Sci. Technol..

[32] Sabaté J., Soret S. (2014). Sustainability of plant-based diets: Back to the future. Am. J. Clin. Nutr..

[33] Glanz K., Rimer B.K., Viswanath K., Orleans C.T. (2008). Health Behavior and Health Education: Theory, Research, and Practice.

[34] Rothgerber H. (2020). Meat-related cognitive dissonance: A conceptual framework for understanding how meat eaters reduce negative arousal from eating animals. Appetite.

[35] Silva Souza L.G., O’Dwyer E. (2022). Animal rights, environment, or health? Effects of argument type and dissonance on the attitudes toward the consumption of animals. Appetite.

[36] Mathur M.B., Peacock J., Reichling D.B., Nadler J., Bain P.A., Gardner C.D., Robinson T.N. (2021). Interventions to reduce meat consumption by appealing to animal welfare: Meta-analysis and evidence-based recommendations. Appetite.

[37] De Boer J., Aiking H. (2022). Considering how farm animal welfare concerns may contribute to more sustainable diets. Appetite.

[38] Alonso M.E., González-Montaña J.R., Lomillos J.M. (2020). Consumers’ concerns and perceptions of farm animal welfare. Animals.

[39] Buller H., Blokhuis H., Jensen P., Keeling L. (2018). Towards farm animal welfare and sustainability. Animals.

[40] Brambell R. (1967). Report of the Technical Committee to Enquire into the Welfare of Animals Kept under Intensive Livestock Husbandry Systems.

[41] Sutherland M.A., Webster J., Sutherland I. (2013). Animal health and welfare issues facing organic production systems. Animals.

[42] Eur-Lex (2008). Council of the European Union Council Directive 2008/120/EC laying down minimum standards for the protection of pigs. Off. J. Eur. Union.

[43] Clark B., Stewart G.B., Panzone L.A., Kyriazakis I., Frewer L.J. (2016). A systematic review of public attitudes, perceptions and behaviours towards production diseases associated with farm animal welfare. J. Agric. Environ. Eth..

[44] Rowlands D.J. (2008). Foot and mouth disease viruses. Encycl. Virol..

[45] Algers B., Blokhuis H.J., Botner A., Broom D.M., Costa P., Domingo M., Greiner M., Hartung J., Koenen F., Müller-Graf C. (2009). Scientific opinion on the overall effects of farming systems on dairy cow—Adopted on 5 June 2009. EFSA J..

[46] Nielsenm S.S., Alvarez J., Bicout D.J., Calistri P., Depner K. (2020). Welfare of pigs at Slaughter_EFSA.Pdf. EFSA Panel Anim. Health Welf..

[47] Pattison J. (1998). The emergence of bovine spongiform encephalopathy and related diseases. Emerg. Infect. Dis..

[48] Ellis W.A., Adler B. (2014). Animal leptospirosis. Leptospira and Leptospirosis.

[49] Collins A.M. (2013). Advances in ileitis control, diagnosis, epidemiology and the economic impacts of disease in commercial pig herds. Agriculture.

[50] Maclachlan N.J., Mayo C.E. (2013). Potential strategies for control of bluetongue, a globally emerging, culicoides-transmitted viral disease of ruminant livestock and wildlife. Antivir. Res..

[51] Espinosa R., Tago D., Treich N. (2020). Infectious diseases and meat production. Environ. Resour. Econ..

[52] (2017). EUR_Lex Directive 98/58/EC—Protection of Animals Kept for Farming Purposes.

[53] Bergeron R., Meunier-Salaun M., Robert S., Faucitano L., Schaefer A. (2008). The welfare of pregnant and lactating sows. Welfare of Pigs. From Birth to Slaughter.

[54] Fleming R.H., McCormack H.A., McTeir L., Whitehead C.C. (2006). Relationships between genetic, environmental and nutritional factors influencing osteoporosis in Lay.Pdf. Br. Poult. Sci..

[55] Baxter M. (1994). The welfare problems of laying hens in battery cages. Vet. Res..

[56] Prunier A., Bonneau M., von Borell E.H., Cinotti S., Gunn M., Fredriksen B., Giersing M., Morton D.B., Tuyttens F.A.M., Velarde A. (2006). A Review of the welfare consequences of surgical castration in piglets and the evaluation of non-surgical methods. Anim. Welf..

[57] Ison S.H., Eddie Clutton R., Di Giminiani P., Rutherford K.M.D. (2016). A review of pain assessment in pigs. Front. Vet. Sci..

[58] Stafford K.J., Mellor D.J. (2011). Addressing the pain associated with disbudding and dehorning in cattle. Appl. Anim. Behav. Sci..

[59] Hudson S.J., Mullord M.M. (1977). Investigations of maternal bonding in dairy cattle. Appl. Anim. Ethol..

[60] Campbell J.M., Crenshaw J.D., Polo J. (2013). The biological stress of early weaned piglets. J. Anim. Sci. Biotechnol..

[61] Hartung J., Marahrens M., Von Holleben K. (2003). Recommendations for future development in cattle transport in Europe. Dtsch. Tierarztl. Wochenschr..

[62] Saxmose Nielsen S., Alvarez J., Joseph Bicout D., Calistri P., Depner K., Ashley Drewe J., Garin-Bastuji B., Luis Gonzales Rojas J., Gort azar Schmidt C., Michel V. (2020). Welfare of pigs at slaughter. Efsa J..

[63] Grandin T. (2010). Auditing animal welfare at slaughter plants. Meat Sci..

[64] Bekoff M., Turner J., D’Silva J. (2005). Animals, Ethics and Trade: The Challenge of Animal Sentience.

[65] Dawkins M.S. (2008). The science of animal suffering. Ethology.

[66] Balcombe J. (2009). Animal pleasure and its moral significance. Appl. Anim. Behav. Sci..

[67] Edgar A.J.L., Lowe J.C., Paul E.S., Nicol C.J., Edgar J.L., Lowe J.C., Paul E.S., Nicol C.J. (2016). Avian maternal response to chick distress. Proc. R. Soc. B Boil. Sci..

[68] Knight S., Barnett L. (2008). Justifying attitudes toward animal use: A qualitative study of people’s views and beliefs. Anthrozoos.

[69] Schuppli C.A., von Keyserlingk M.A.G., Weary D.M. (2014). Access to pasture for dairy cows: Responses from an online engagement. J. Anim. Sci..

[70] Weary D.M., Schuppli C.A., von Keyserlingk M.A.G. (2011). Tail docking dairy cattle: Responses from an online engagement. J. Anim. Sci..

[71] Vandresen B., Hötzel M.J. (2021). Pets as family and pigs in crates: Public attitudes towards farrowing crates. Appl. Anim. Behav. Sci..

[72] Cardoso C.S., Von Keyserlingk M.A.G., Hötzel M.J. (2017). Brazilian citizens: Expectations regarding dairy cattle welfare and awareness of contentious practices. Animals.

[73] Toma L., Stott A.W., Revoredo-Giha C., Kupiec-Teahan B. (2012). Consumers and animal welfare. A comparison between European Union Countries. Appetite.

[74] Lagerkvist C.J., Hess S. (2011). A meta-analysis of consumer willingness to pay for farm animal welfare. Eur. Rev. Agric. Econ..

[75] Frewer L.J., Kole A., van de Kroon S.M., de Lauwere C. (2005). Consumer attitudes towards the development of animal-friendly husbandry systems. J. Agric. Environ. Ethics.

[76] García-Gudiño J., Blanco-Penedo I., Gispert M., Brun A., Perea J., Font-I-Furnols M. (2020). Understanding consumers’ perceptions towards Iberian pig production and animal welfare. Meat Sci..

[77] Te Velde H., Aarts N., Van Woerkum C. (2002). Dealing with ambivalence: Farmers’ and consumers’ perceptions of animal welfare in livestock breeding. J. Agric. Environ. Eth..

[78] De Jonge J., van Trijp H.C.M. (2013). The impact of broiler production system practices on consumer perceptions of animal welfare. Poult. Sci..

[79] Tomasevic I., Bahelka I., Čandek-Potokar M., Čítek J., Djekić I., Djurkin Kušec I., Getya A., Guerrero L., Iordăchescu G., Ivanova S. (2020). Attitudes and beliefs of eastern european consumers towards piglet castration and meat from castrated pigs. Meat Sci..

[80] Bell E., Norwood F.B., Lusk J.L. (2017). Are consumers wilfully ignorant about animal welfare?. Anim. Welf..

[81] Mckendree M.G.S., Croney C.C., Widmar N.J.O. (2014). Effects of demographic factors and information sources on united states consumer perceptions of animal welfare. J. Anim. Sci..

[82] Foster E.D., Deardorff A. (2017). Open Science Framework (OSF). JMLA.

[83] Fonseca R.P., Sanchez-Sabate R. Consumers Attitudes towards Animal Suffering. A Systematic Review On Awareness, Willingness and Dietary Change. https://osf.io/pxrua/.

[84] OSF. https://osf.io/.

[85] Moher D., Liberati A., Tetzlaff J., Altman D.G., Altman D., Antes G., Atkins D., Barbour V., Barrowman N., Berlin J.A. (2009). Preferred reporting items for systematic reviews and meta-analyses: The PRISMA statement. PLoS Med..

[86] Ryan E.B., Fraser D., Weary D.M. (2015). Public attitudes to housing systems for pregnant pigs. PLoS ONE.

[87] Ventura B.A., von Keyserlingk M.A.G., Schuppli C.A., Weary D.M. (2013). Views on contentious practices in dairy farming: The case of early cow-calf separation. J. Dairy Sci..

[88] Ventura B.A., Von Keyserlingk M.A.G., Wittman H., Weary D.M. (2016). What difference does a visit make? Changes in animal welfare perceptions after interested citizens tour a dairy farm. PLoS ONE.

[89] Robbins J.A., Roberts C., Weary D.M., Franks B., von Keyserlingk M.A.G. (2019). Factors influencing public support for dairy tie stall housing in the US. PLoS ONE.

[90] Earle M., Hodson G., Dhont K., MacInnis C. (2019). Eating with our eyes (closed): Effects of visually associating animals with meat on antivegan/vegetarian attitudes and meat consumption willingness. Group Process Intergroup Relat..

[91] Kunst J.R., Palacios Haugestad C.A. (2018). The effects of dissociation on willingness to eat meat are moderated by exposure to unprocessed meat: A cross-cultural demonstration. Appetite.

[92] Haile M., Jalil A., Tasoff J., Vargas Bustamante A. (2021). Changing hearts and plates: The effect of animal-advocacy pamphlets on meat consumption. Front. Psychol..

[93] Johnson C., Schreer G., Bao K.J. (2021). Effect of anthropomorphizing food animals on intentions to eat meat. Anthrozoos.

[94] Herchenroeder L., Forestell C.A., Bravo A.J. (2022). The effectiveness of animal welfare-, environmental-, and health-focused video appeals on implicit and explicit wanting of meat and intentions to reduce meat consumption. J. Soc. Psychol..

[95] Zickfeld J.H., Kunst J.R., Hohle S.M. (2018). Too sweet to eat: Exploring the effects of cuteness on meat consumption. Appetite.

[96] Kunst J.R., Hohle S.M. (2016). Meat eaters by dissociation: How we present, prepare and talk about meat increases willingness to eat meat by reducing empathy and disgust. Appetite.

[97] Palomo-Vélez G., Tybur J.M., van Vugt M. (2018). Unsustainable, unhealthy, or disgusting? Comparing different persuasive messages against meat consumption. J. Environ. Psychol..

[98] Leach S., Piazza J., Loughnan S., Sutton R.M., Kapantai I., Dhont K., Douglas K.M. (2022). Unpalatable truths: Commitment to eating meat is associated with strategic ignorance of food-animal minds. Appetite.

[99] Piazza J., McLatchie N., Olesen C. (2018). Are baby animals less appetizing? tenderness toward baby animals and appetite for meat. Anthrozoos.

[100] Anderson E.C., Barrett L.F. (2016). Affective beliefs influence the experience of eating meat. PLoS ONE.

[101] Monteiro C.A., Pfeiler T.M., Patterson M.D., Milburn M.A. (2017). The carnism inventory: Measuring the ideology of eating animals. Appetite.

[102] Bratanova B., Loughnan S., Bastian B. (2011). The effect of categorization as food on the perceived moral standing of animals. Appetite.

[103] Olynk Widmar N., Morgan C.J., Wolf A.C., Yeager A.E., Dominick S.R., Croney C.C. (2017). US resident perceptions of dairy cattle management practices. Agric. Sci..

[104] Sato P., Hötzel M.J., Von Keyserlingk M.A.G. (2017). American citizens’ views of an ideal pig farm. Animals.

[105] Tonsor G.T., Wolf C., Olynk N. (2009). Consumer voting and demand behavior regarding swine gestation crates. Food Policy.

[106] Estévez-Moreno L.X., María G.A., Sepúlveda W.S., Villarroel M., Miranda-de la Lama G.C. (2021). Attitudes of meat consumers in mexico and spain about farm animal welfare: A cross-cultural study. Meat Sci..

[107] Miranda-de la Lama G.C., Estévez-Moreno L.X., Sepúlveda W.S., Estrada-Chavero M.C., Rayas-Amor A.A., Villarroel M., María G.A. (2017). Mexican consumers’ perceptions and attitudes towards farm animal welfare and willingness to pay for welfare friendly meat products. Meat Sci..

[108] Estévez-Moreno L.X., Miranda-de la Lama G.C., Miguel-Pacheco G.G. (2022). Consumer attitudes towards farm animal welfare in argentina, chile, colombia, ecuador, peru and bolivia: A segmentation-based study. Meat Sci..

[109] Vanhonacker F., Tuyttens F.A.M., Verbeke W. (2016). Belgian citizens’ and broiler producers’ perceptions of broiler chicken welfare in belgium versus brazil. Poult. Sci..

[110] De Queiroz R.G., de Faria Domingues C.H., Canozzi M.E.A., Garcia R.G., Ruviaro C.F., Barcellos J.O.J., Borges J.A.R. (2018). How do brazilian citizens perceive animal welfare conditions in poultry, beef, and dairy supply chains?. PLoS ONE.

[111] Yunes M.C., Von Keyserlingk M.A.G., Hötzel M.J. (2017). Brazilian citizens’ opinions and attitudes about farm animal production systems. Animals.

[112] Hötzel M.J., Cardoso C.S., Roslindo A., von Keyserlingk M.A.G. (2017). Citizens’ views on the practices of zero-grazing and cow-calf separation in the dairy industry: Does providing information increase acceptability?. J. Dairy Sci..

[113] Soriano V.S., Phillips C.J.C., Taconeli C.A., Fragoso A.A.H., Molento C.F.M. (2021). Mind the gap: Animal protection law and opinion of sheep farmers and lay citizens regarding animal maltreatment in sheep farming in southern brazil. Animals.

[114] Vandresen B., Hötzel M.J. (2021). “Mothers Should Have Freedom of Movement”—Citizens’ attitudes regarding farrowing housing systems for sows and their piglets. Animals.

[115] Tamioso P.R., Rucinque D.S., Miele M., Boissy A., Molento C.F.M. (2018). Perception of animal sentience by brazilian and french citizens: The case of sheep welfare and sentience. PLoS ONE.

[116] Teixeira D.L., Larraín R., Hötzel M.J. (2018). Are views towards egg farming associated with brazilian and chilean egg consumers’ purchasing habits?. PLoS ONE.

[117] Krystallis A., de Barcellos M.D., Kügler J.O., Verbeke W., Grunert K.G. (2009). Attitudes of european citizens towards pig production systems. Livest. Sci..

[118] Vanhonacker F., Verbeke W., Van Poucke E., Buijs S., Tuyttens F.A.M. (2009). Societal concern related to stocking density, pen size and group size in farm animal production. Livest. Sci..

[119] Aluwé M., Heyrman E., Almeida J.M., Babol J., Battacone G., Čítek J., Furnols M.F.I., Getya A., Karolyi D., Kostyra E. (2020). Exploratory survey on european consumer and stakeholder attitudes towards alternatives for surgical castration of piglets. Animals.

[120] Herrewijn L., De Groeve B., Cauberghe V., Hudders L. (2021). VR outreach and meat reduction advocacy: The role of presence, empathic concern and speciesism in predicting meat reduction intentions. Appetite.

[121] Kupsala S., Vinnari M., Jokinen P., Räsänen P. (2016). Public Perceptions of mental capacities of nonhuman animals: Finnish population survey. Soc. Anim..

[122] Kupsala S., Vinnari M., Jokinen P., Räsänen P. (2015). Citizen attitudes to farm animals in finland: A population-based study. J. Agric. Environ. Eth..

[123] Clarkid B., Panzone L.A., Stewart G.B., Kyriazakis I., Niemi J.K., Latvala T., Tranter R., Jones P., Frewer L.J. (2019). Consumer attitudes towards production diseases in intensive production systems. Plos ONE.

[124] Tian Q., Hilton D., Becker M. (2016). Confronting the meat paradox in different cultural contexts: Reactions among chinese and french participants. Appetite.

[125] Heid A., Hamm U. (2012). Consumer attitudes towards alternatives to piglet castration without pain relief in organic farming: Qualitative results from germany. J. Agric. Environ. Eth..

[126] Christoph-Schulz I., Rovers A.K. (2020). German citizens’ perception of fattening pig husbandry—Evidence from a mixed methods approach. Agriculture.

[127] Sonntag W.I., Spiller A. (2018). Measuring public concerns? Developing a moral concerns scale regarding non-product related process and production methods. Sustainability.

[128] Wernsmann A., Wildraut C., Südwestfalen F., Von Meyer-Höfer M., Mergenthaler M. (2018). Perception and evaluation of a pig fattening pen based on film material in an online survey experiment with german citizens. GJAE.

[129] Cordts A., Nitzko S., Spiller A. (2014). Consumer response to negative information on meat consumption in germany. Int. Food Agribus. Manag. Rev..

[130] Busch G., Gauly S., Von Meyer-Höfer M., Spiller A. (2019). Does picture background matter? People’s evaluation of pigs in different farm settings. PLoS ONE.

[131] Weible D., Christoph-Schulz I., Salamon P., Zander K. (2016). Citizens’ perception of modern pig production in germany: A mixed-method research approach. Br. Food J..

[132] Weinrich R., Kühl S., Zühlsdorf A., Spiller A. (2014). Consumer attitudes in germany towards different dairy housing systems and their implications for the marketing of pasture raised milk. Int. Food Agribus. Manag. Review.

[133] Vanhonacker F., Verbeke W. (2011). Consumer response to the possible use of a vaccine method to control boar taint v. physical piglet castration with anaesthesia: A quantitative study in four european countries. Animal.

[134] Hartmann C., Siegrist M. (2020). Our daily meat: Justification, moral evaluation and willingness to substitute. Food Qual. Prefer..

[135] Kühl S., Gauly S., Spiller A. (2019). Analysing public acceptance of four common husbandry systems for dairy cattle using a picture-based approach. Livest. Sci..

[136] Rovers A., Sonntag W.I., Brümmer N., Christoph-Schulz I. (2018). Citizens’ perception of recent livestock production systems in Germany. Ger. J. Agric. Econ..

[137] Peden R.S.E., Camerlink I., Boyle L.A., Loughnan S., Akaichi F., Turner S.P. (2020). Belief in pigs’ capacity to suffer: An assessment of pig farmers, veterinarians, students, and citizens. Anthrozoos.

[138] Phillips C.J.C., Izmirli S., Aldavood S.J., Alonso M., Choe B.I., Hanlon A., Handziska A., Illmann G., Keeling L., Kennedy M. (2012). Students’ attitudes to animal welfare and rights in Europe and Asia. Anim. Welf..

[139] De Haas E.N., Oliemans E., van Gerwen M.A.A.M. (2021). The need for an alternative to culling day-old male layer chicks: A survey on awareness, alternatives, and the willingness to pay for alternatives in a selected population of dutch citizens. Front. Vet. Sci..

[140] Gremmen B., Bruijnis M.R.N., Blok V., Stassen E.N. (2018). A public survey on handling male chicks in the dutch egg sector. J. Agric. Environ. Eth..

[141] Bergstra T., Hogeveen H., Erno Kuiper W., Oude Lansink A.G.J.M., Stassen E.N. (2017). Attitudes of dutch citizens toward sow husbandry with regard to animals, humans, and the environment. Anthrozoos.

[142] De Jonge J., van Trijp H.C.M. (2013). Meeting heterogeneity in consumer demand for animal welfare: A reflection on existing knowledge and implications for the meat sector. J. Agric. Environ. Eth..

[143] Bergstra T.J., Gremmen B., Stassen E.N. (2015). Moral values and attitudes toward dutch sow husbandry. J. Agric. Environ. Eth..

[144] Sødring M., Nafstad O., Håseth T.T. (2020). Change in norwegian consumer attitudes towards piglet castration: Increased emphasis on animal welfare. Acta Vet. Scand..

[145] Fredriksen B., Johnsen A.M.S., Skuterud E. (2011). Consumer attitudes towards castration of piglets and alternatives to surgical castration. Res. Vet. Sci..

[146] Piazza J., Loughnan S. (2016). When meat gets personal, animals’ minds matter less: Motivated use of intelligence information in judgments of moral standing. Soc. Psychol. Personal. Sci..

[147] Tawse J. (2010). Consumer attitudes towards farm animals and their welfare: A pig production case study. Biosci. Horizons.

[148] Connor M., Cowan S.L. (2020). Consumer evaluation of farm animal mutilations. Res. Vet. Sci..

[149] Fuseini A., Knowles T.G. (2020). The ethics of halal meat consumption: Preferences of consumers in england according to the method of slaughter. Vet. Rec..

[150] Fuseini A., Wotton S.B., Hadley P.J., Knowles T.G. (2017). The perception and acceptability of pre-slaughter and post-slaughter stunning for halal production: The views of UK islamic scholars and halal consumers. Meat Sci..

[151] Tomasevic I., Bahelka I., Čítek J., Čandek-Potokar M., Djekić I., Getya A., Guerrero L., Ivanova S., Kušec G., Nakov D. (2020). Attitudes and beliefs of eastern european consumers towards animal welfare. Animals.

[152] Bray H.J., Ankeny R.A. (2017). happy chickens lay tastier eggs: Motivations for buying free-range eggs in Australia. Anthrozoos.

[153] Buddle E.A., Bray H.J., Ankeny R.A. (2018). Why would we believe them? meat consumers’ reactions to online farm animal welfare activism in Australia. Commun. Res. Pract..

[154] McGrath N., Walker J., Nilsson D., Phillips C. (2013). Public attitudes towards grief in animals. Anim. Welf..

[155] Malek L., Umberger W.J., Rolfe J. (2018). Segmentation of Australian meat consumers on the basis of attitudes regarding farm animal welfare and the environmental impact of meat production. Anim. Prod. Sci..

[156] Dowsett E., Semmler C., Bray H., Ankeny R.A., Chur-Hansen A. (2018). Neutralising the meat paradox: Cognitive dissonance, gender, and eating animals. Appetite.

[157] Bastian B., Loughnan S., Haslam N., Radke H.R.M. (2012). Don’t mind meat? The denial of mind to animals used for human consumption. Personal. Soc. Psychol. Bull..

[158] Walker J.K., McGrath N., Handel I.G., Waran N.K., Phillips C.J.C. (2014). Does owning a companion animal influence the belief that animals experience emotions such as grief?. Anim. Welf..

[159] Coleman G., Jongman E., Greenfield L., Hemsworth P. (2016). Farmer and public attitudes toward lamb finishing systems. J. Appl. Anim. Welf. Sci..

[160] Tiplady C.M., Walsh D.A.B., Phillips C.J.C. (2013). Public response to media coverage of animal cruelty. J. Agric. Environ. Eth..

[161] Syazwani N., Jalil A., Tawde A.V., Zito S., Sinclair Id M., Fryer C., Idrus Z., Phillips C.J.C. (2018). Attitudes of the public towards halal food and associated animal welfare issues in two countries with predominantly muslim and non-muslim populations. PLoS ONE.

[162] Rice M., Hemsworth L.M., Hemsworth P.H., Coleman G.J. (2020). The impact of a negative media event on public attitudes towards animal welfare in the red meat industry. Animals.

[163] Lemos Teixeira D., Larraín R., Melo O., Hö Tzel M.J. (2018). Public opinion towards castration without anaesthesia and lack of access to pasture in beef cattle production. Plos ONE.

[164] Hötzel M.J., Yunes M.C., Vandresen B., Albernaz-Gonçalves R., Woodroffe R.E. (2020). On the road to end pig pain: Knowledge and attitudes of brazilian citizens regarding castration. Animals.

[165] Van Beirendonck S., Driessen B., Geers R. (2013). Belgian consumers’ opinion on pork consumption concerning alternatives for unanesthetized piglet castration. J. Agric. Environ. Eth..

[166] Vanhonacker F., Verbeke W., Tuyttens F.A.M. (2009). Belgian consumers’ attitude towards surgical castration and immuno-castration of piglets. Anim. Welf..

[167] Sonntag W.I., Spiller A., Von Meyer-Hofer M. (2019). Discussing modern poultry farming systems—Insights into citizen’s lay theories. Poult. Sci..

[168] Tonsor G.T., Olynk N., Wolf C. (2009). Consumer preferences for animal welfare attributes: The case of gestation crates. J. Agric. Appl. Econ..

[169] Yunes M.C., Keyserlingk M.A.G.V., Hötzel M.J. (2018). Restricting the ability of sows to move: A source of concern for some Brazilians. Anim. Welf..

[170] Benningstad N.C.G., Kunst J.R. (2020). Dissociating meat from its animal origins: A systematic literature review. Appetite.

[171] Bastian B., Costello K., Loughnan S., Hodson G. (2012). When closing the human-animal divide expands moral concern: The importance of framing. Soc. Psychol. Personal. Sci..

[172] Loughnan S., Haslam N., Bastian B. (2010). The role of meat consumption in the denial of moral status and mind to meat animals. Appetite.

[173] Dijkstra A., Rotelli V. (2022). Lowering red meat and processed meat consumption with environmental, animal welfare, and health arguments in Italy: An online experiment. Front. Psychol..

[174] Niemyjska A., Cantarero K., Byrka K., Bilewicz M. (2018). Too humanlike to increase my appetite: Disposition to anthropomorphize animals relates to decreased meat consumption through empathic concern. Appetite.

[175] Fonseca R.P. (2020). “120 Em 60”: Práticas e atitudes de trabalhadores para com animais num matadouro português. Rev. Sociol. Probl. Práticas.

[176] Fonseca R.P. (2022). Animal farming impacts. critical overview of primary school books. J. Agric. Environ. Eth..

[177] Pedersen H. (2004). Schools, speciesism, and hidden curricula: The role of critical pedagogy for humane education futures bringing the human-animal relation into education research. J. Futur. Stud..

[178] Bryant C.J. (2019). We can’t keep meating like this: Attitudes towards vegetarian and vegan diets in the united kingdom. Sustainability.

[179] Rothgerber H. (2014). Efforts to overcome vegetarian-induced dissonance among meat eaters. Appetite.

[180] Schnettler M.B., Vidal M.R., Silva F.R., Vallejos C.L., Sepúlveda B.N. (2008). Consumer perception of animal welfare and livestock production in the araucania region, chile. Chil. J. Agric. Res..

[181] Kubberod E., Risvik E. (2002). Attitudes towards meat and meat eating among adolescents in norway: A attitudes towards meat and meat-eating among adolescents in norway: A qualitative study. Appetite.

[182] Gossard M.H., York R. (2003). Social structural influences on meat consumption. Hum. Ecol. Rev..

[183] Einhorn L. (2021). Meat consumption, classed?: The socioeconomic underpinnings of dietary change. Osterr. Z. Fur. Soziol..

[184] Koch F., Heuer T., Krems C., Claupein E. (2019). Meat consumers and non-meat consumers in germany: A characterisation based on results of the german national nutrition survey II. J. Nutr. Sci..

[185] Sanchez-Sabate R., Sabaté J. (2019). Consumer attitudes towards environmental concerns of meat consumption: A systematic review. Int. J. Environ. Res. Public Health.

[186] Graça J., Calheiros M.M., Oliveira A. (2015). Attached to meat? (Un)Willingness and intentions to adopt a more plant-based diet. Appetite.

[187] Fiddes N. (1992). Meat: A Natural Symbol.

[188] Lloro-Bidart T., Banschbach V.S. (2019). Animals in Environmental Education Interdisciplinary Approaches to Curriculum and Pedagogy.

[189] Kopnina H. (2012). Education for sustainable development (ESD): The turn away from “environment” in environmental education?. Environ. Educ. Res..

[190] Love H.J., Sulikowski D. (2018). Of meat and men: Sex differences in implicit and explicit attitudes toward meat. Front. Psychol..

[191] De Backer C., Erreygers S., De Cort C., Vandermoere F., Dhoest A., Vrinten J., Van Bauwel S. (2020). Meat and masculinities. can differences in masculinity predict meat consumption, intentions to reduce meat and attitudes towards vegetarians?. Appetite.

[192] Sobal J. (2005). Men, meat, and marriage: Models of masculinity. Food Foodways.

[193] Fonseca R.P. (2017). A construção de masculinidades através da carne vermelha: Dois casos de estudo na publicidade portuguesa. Tropos Comun. Soc. Cult..

[194] Rothgerber H. (2013). Real men don’t eat (vegetable) quiche: Masculinity and the justification of meat consumption. Psychol. Men Masc..

[195] Vandermoere F., Geerts R., De Backer C., Erreygers S., Van Doorslaer E. (2019). Meat consumption and vegaphobia: An exploration of the characteristics of meat eaters, veg173 aphobes, and their social environment. Sustainability.

[196] Markowski K.L., Roxburgh S. (2019). “If I became a vegan, my family and friends would hate me Anticipating vegan stigma as a barrier to plant-based diets. Appetite.

[197] Kwasny T., Dobernig K., Riefler P. (2022). Towards reduced meat consumption: A systematic literature review of intervention effectiveness, 2001–2019. Appetite.

