# Supplementary material for: Consumers’ Attitudes towards Animal Suffering: A Systematic Review on Awareness, Willingness and Dietary Change
Source: Int J Environ Res Public Health. 2022 Dec 6;19(23):16372. doi: 10.3390/ijerph192316372 (PMC9741386; doi:10.3390/ijerph192316372)
Supplement: Supplementary file 1 [file ijerph-19-16372-s001.zip › Table Awareness (3).pdf]

Supplementary Materials

Table S1 - Consumers' awareness of animal suffering in animal agriculture

Authors, year	Design; year data collected	Country; sample	Main research question	(Type of?) Information provided on animal suffering	Outcome measure:		
					Question or dependent variable	Response or finding	Effects of covariates
(Aluwé et al., 2020) [1]	Online survey; 2020	European pork consumers Total N=4278 Belgium (N=417) Bulgaria (N=227), Croatia (N=208); Czechia (N=226), Switzerland (), Germany (), Spain (N=253), France (N=213), Croatia (N=208), Italy (N=204),	Consumer and stakeholder attitudes towards alternatives for surgical castration of piglets	Participants received basic information about each practice and reason of piglet castration followed by an explanation about the 4 options of how male pigs can be produced. Information was presented in an infographic instead of only via text to enhance information capture by the consumer	Awareness on how pork is mainly produced;<br><br>Without anaesthesia / analgesia (acceptability / Likert scale)	Awareness Overall, 59%<br><br>10% acceptability rate and 61% not acceptability for castration without anaesthesia	Awareness<br><br>Belgium (48%) Bulgaria (50%), Croatia (48%) Czechia (61%), Switzerland (-), Germany (42%), Spain (41%), France (63%), Croatia (-), Italy (29%), Netherlands (-), Norway (47%), Poland (31%), Portugal (52%), Romania (29%), Russia (29%), Serbia (33%), Slovenia (not available), Sweden (67%), Ukraine (49%)<br><br>Castration without

		Netherlands (), Norway (N=177), Poland (N=210), Portugal (N=191), Romania (N=224), Russia (N=224), Serbia (N=352), Slovenia (not available), Sweden (N=258), Ukraine (N=255)					anaesthesia (not acceptable): Belgium (87%) Bulgaria (37%), Croatia (52%) Czechia (38%), Germany (76%), Spain (72%), France (74%), Croatia (N=208), Italy (74%), Poland (68%), Portugal (66%), Romania (55%), Russia (48%), Serbia (54%), Sweden (80%), Ukraine (31%)<br><br>Male respondents agreed more with the practice of castration without anaesthesia
(Anderson & Barret, 2016) [2]	Interviews, experimental; 2016	U.S. undergraduate students, N=248 people (study 2)	To access participants' beliefs about meat eating experience	Two provided labels: animals grazing outdoors; animals confined to indoor pens + 2.5 grams of	Study 2: if beliefs about how animals were raised can influence the experience of meat eating	Descriptions influenced meat liking, $F(3, 244) = 3.25$, $p < 0.023$, $\eta 2\ p = 0.038$	Factory farm description was less liked compared to the outdoor farm, $t(119) = 2.52$, $p < 0.014$, and control description, $t(119) = 2.33$, $p < 0.022$

				meat			
(Bastian et al., 2012a) [3]	To write essay; N.D.	Undergraduate students from Canada, N=36, 22 women (Study 1)	If framing similarities between humans and animals has consequences for the extension of moral concern towards other animals	Study 1: measure of mind attribution required participants to view a picture of a cow	Study 1: similarities between humans and animals (pain, hunger, pleasure, fear, happiness, consciousness, seeing, hearing, tasting, thinking, imagining, wishing, needing desire, intending, planning, choosing, reasoning)	Participants who wrote animals-are-human-like indicated more inclusive moral circles (M ¼ 20.44, SD ¼ 6.06) than those who wrote humans-are-animal-like (M ¼ 14.06, SD ¼ 8.02), t(34) ¼ 2.70, p ¼ .011, d ¼ 0.94. Participants who wrote animals-are-human-like attributed more sensation to the cow (M ¼ 6.15, SD ¼ .60) compared with those who wrote humans-are-animal-like (M ¼ 5.43, SD ¼ 1.18), t(34) ¼ 2.32, p ¼ .027, d ¼ 0.80. This pattern was evident for	n.a.

						intellect (animals-are-human-like: M ¼ 4.08, SD ¼ 1.65; humans-are-animal-like: M ¼ 3.38, SD ¼ 1.08; t(34) ¼ 1.51, p ¼ .14, d ¼ 0.52)	
(Bastian et al., 2012b) [4]	Experimental, questionnaire, N.D.	Australian students N=71, 59 women; Meat-eating students; N=66, 43 women;	Denial of food-animals minds, especially when meat eaters are reminded of the link between meat and animal suffering	No info.	Study 1: rating 32 animals in terms of mental capacities (hunger, fear, pleasure, pain, rage)<br>Study 2: completing two versions of questionnaires that required them to look at a picture of a cow and a sheep	Study 1: perceived mind negatively associated with the animal's edibility (r = –.42, p < .001; positively with feeling bad about eating the animal (r = .77, p < .001) and with how morally wrong it would be to eat the animal (r = .80, p < .001)<br>Study 2: when reminded that an animal would be used for food, meat eaters denied animals mental capacities (:M = 4.08, SD =	n.a.

						.86) compared to when no such reminders where provided (non food animal: M = 4.30, SD = .82), t(65) = 3.24, p = .002	
(Beirendonck et al., 2013) [5]	Questionnaire; 2009	Belgian consumers, N=1018	Consumers' opinion on alternatives for unanesthetized piglet castration	The explanation of unanesthetized piglet castration was given as follows: during unanesthetized piglet castration, the testicles are removed without anaesthesia or pain relief. (...)This is a quick and cheap method, but also painful and stressful for the animals. Another example is the description of	Awareness: Are you aware of male piglets being castrated? Are you aware that this happens without anaesthesia or analgesia? Do you know the reason for piglet castration? Need for alternatives: Do you think alternatives to unanesthetized piglet castration are necessary? Do you think unanesthetized piglet castration should be banned? Acceptability:	Awareness: low consumers' awareness regarding piglet castration 45.9%, only 30.2% knew why piglets are castrated; 45.9% awareness regarding it takes place without anaesthesia or analgesia Acceptability of alternatives and preference: 98.5%, agreed that castration under anaesthesia and analgesia is acceptable	Awareness: 45.9% were aware of piglet castration. This was associated with the living environment (p = 0.0422), education level (p = 0.0448); 69.8% could not explain the reason for piglet castration; Alternatives: agree (1-2 times consumption of pork for week - 97.4%; 3-4 times - 93.4%; more - 33.3%; Banning of unanesthetized castration: (consumption pork 1-2 times for week - 74.4%; 3-4 - 69.9%;

				immunovaccination: The purpose of immunovaccination is to make castration unnecessary by stemming the development of the testicles. The injected hormone like substances influence the hormonal system of the pig so that puberty is delayed. This means that the risk of boar taint is reduced strongly, but (...) it cannot be guaranteed a 100% that boar taint cannot occur	Do you think castration with anaesthesia and analgesia is an acceptable alternative?<br>Do you think raising entire males is an acceptable alternative?<br>Do you think immunovaccination is an acceptable alternative?		More - 0.0%<br>Acceptability: (consumption pork 1-2 times for week - 98.6%; 3-4 times - 97.8%; More - 66.7%<br>Awareness: Rural - 49.3%; urban - 43.0%<br>Education - high school 40.6; higher education 49.4%; university 47.8%<br>Banning of unanesthetized castration: men 68.7%; women 75.4%;<br>Education, high school 67.4%; 75.3%; university 73.6%<br>Alternatives: 96.3% felt that it was necessary to find alternative; 72.0% wanted unanesthetized castration to be banned;<br>Opinion on the need for alternatives was

							associated with pork consumption per week (p = 0.0018); The opinion on a ban for unanesthetized piglet castration was influenced by gender (stronger support for a ban among females p = 0.0188), education level (stronger support for a ban among higher educated respondents; p = 0.0445); Pork consumption per week (stronger support for a ban among respondents that never eat pork; p = 0.0208) Acceptability of Alternatives: castration under anaesthesia had the most positive response, followed by immunovaccination,

							while raising entire males was least acceptable.<br>More men than women found immunovaccination acceptable; p = 0.0199
(Benningstad et al., 2020) [6]	Systematic literature review; 2019	Several	Consumers' tendency to dissociate meat from its animal origins	n.a.	Awareness: About meat and animal origins	Presentation of animal foods, food vocabulary reduced consumers' ability to reflect upon the animal origins (Evans and Miele 2012);<br>Removing its animal characteristics (e.g.,<br>the head of an animal) facilitated dissociation (Kunst and Hohle 2016);<br>Observing transportation of animals to the slaughterhouse triggers animal-meat connection (Holm 2018);	Gender differences exist often for animal welfare (Leroy & Praet, 2015; Rosenfeld, 2018; Rothgerber, 2013; Ruby, 2012);<br>Age differences not conclusive;<br>Rural consumers may be less affected by interruptions of the dissociation process and, thus, have less of a need to use it as a strategy to<br>Reduce discomfort (Bray et al. 2016; Kubberød et al. (2002)

						Perceptions of cuteness linked to caring responses (Zickfeld et al. 2018; Piazza et al. 2018).<br><br>Try not to think of the lives and deaths of the animals is central strategy used when consuming meat (Graça et al. 2014; Bandura, 1999)	
(Bergstra et al., 2015) [7]	Online questionnaire; 2011	Dutch citizens; N=2572 (Additional Care - AC) Cl1 high-AC cluster N=645), Cl2 moderate-AC cluster (N=623), Cl3 Max-AC cluster	Moral values and attitudes toward sow husbandry	No info.	Metabolic/physical exhaustion<br><br>Fear/anxiety<br><br>Pain<br><br>Number of kept animals<br><br>Number of animals per m2	(five-point scale (1: no Additional Care necessary, 5: maximal Additional Care)<br><br>Metabolic/physical exhaustion (Cl1) 3.7, (Cl2) 3.0, (Cl3) 4.5, (Cl4) 1.7<br><br>Fear/anxiety	n. a.

		(N=225), Cl4 no-AC cluster (N=114) (farmers not included)			Tail docking<br><br>Castration<br><br>Weaning age<br><br>Motherless care	(Cl1) 4.3, (Cl2) 3.3, (Cl3) 4.8, (Cl4) 2.1<br><br>Pain (Cl1) 4.4, (Cl2) 3.3, (Cl3) 4.8, (Cl4) 2.0<br><br>Number of animals per m2 (Cl1) 4.4, (Cl2) 3.4, (Cl3) 4.8, (Cl4) 2.0<br><br>Floor cover (Cl1) 4.3, (Cl2) 3.3, (Cl3) 4.8, (Cl4) 2.0<br><br>Tail docking (Cl1) 4.2, (Cl2) 3.1, (Cl3) 4.7, (Cl4) 1.7<br><br>Castration (Cl1) 4.2, (Cl2) 3.0, (Cl3) 4.7, (Cl4) 1.7<br><br>Weaning age (Cl1) 4.1, (Cl2) 3.0, (Cl3) 4.7, (Cl4) 1.6	

						(Level of agreement (D: disagree, N: neutral, A: agree)<br><br>Pigs have intrinsic value<br>(CI1) D 2.6, N 18.6, A 78.8<br>(CI2) D 7.9, N 44.3, A 47.8;<br>(CI3) D 1.8, N 15.6, A 82.7,<br>(CI4) D 26.3, N 41.2, A 32.5<br><br>Pigs are sentient<br>(CI1) D 1.4, N 10.2. A 88.4.<br>(CI2) A 3.4. N 26.8. A 69.8<br>(CI3) D 0.9. N 9.8. A 89.3<br>(CI4) A 9.6, N 21.1, A 69.3<br><br>Pig is meat for humans<br>(CI1) D 31.5, N 36.6, A 31.9<br>(CI2) D 14.3, N 41.9, A 43.8<br>(CI3) D 31.1, N 30.7, A 38.2.	

						(CI4) D 4.4, N 25.4, A 70.2	
(Bergstra et al., 2017) [8]	Questionnaire; 2011	Dutch respondents, N=1607, 47.7% women	To determine the attitudes of Dutch people toward sow husbandry with regard to animals, humans, and environment	Selected issues related to pig husbandry that were presented at least two times as a news item on the website of at least one of the animal organisations in the years 2009 to 2011. These issues were: piglet mortality, pig housing, scale increase (increase production and decrease production costs), interventions (castration, tail docking), euthanasia, sow lifespan, litter size, weaning age,	(Acceptability, Likert scale)<br>Piglet mortality;<br>Weaning age;<br>The castration of piglets;<br>The docking of tails of piglets;<br>Interventions (castration/tail docking) without sedation;<br>Housing pigs inside for their entire lifetime;<br>Keep sows in farrowing pens (iron fences on both sides of the sow to minimise piglet mortality) until the piglets are separated from the sow	More than 50% of the respondents indicated that much or utmost AC (Additional Care) was necessary for most issues except for metabolic/physical exhaustion, freedom to act	Indication of Strong AC (Additional Care) (SAC) and Utmost AC (UAC):<br>Disease/infection/injuries 39.6% SAC, 25.9%UAC;<br>Fear/anxiety 36.3% SAC, 28.7% UAC;<br>Pain 35.5% SAC 30.9%UAC<br>Number of animals per m2 34.0% SAC, 33.3% UAC<br>Castration 27.4% SAC, 28.4% UAC<br>Tail docking 27.4% SAC, 28.4% UAC<br>Weaning age 31.5% SAC, 21.9% UAC<br>Females have more negative attitudes toward animal husbandry practices, such as sow husbandry, than males;<br>Older respondents had more negative attitudes toward

				motherless care, use of antibiotics, transport, and use of antibiotics			sow husbandry than younger respondents
(Bratanova et al., 2011) [9]	Online survey; N.D.	U.S. citizens, N=80, 46 women	If categorising an animal as ‘food’ may diminish our perceived capacity to suffer, which in turn dampens our moral concern	Rating capacity to suffer (Animal; Accidental-Death; Collected-Meat; Hunted-Meat)	Types of animal dying condition: Animal death; Accidental; Collected-Meat; Hunted-Meat	Animals classified as food are seen as less able to suffer regardless of whether they are deliberately killed by humans: More capacity to suffer in the Animal condition compared with the Hunted-Meat and Collected-Meat conditions, ts(40/38) > 2.8, ps < 0.01.	n.a.
(Bray & Ankeny 2017) [10]	Focus groups and interviews; 2014	Australia, Participants N=73, 70% women	Participants explanations of their purchasing decisions in terms of ethical consumption	No info.	Consumers’ motivations for buying free-range (or cage-free)	High levels of awareness of caged-egg production;<br><br>“Confinement is seen to restrict natural behaviours.” “Strongly held perceptions that	n.a.

						caged-egg production is “wrong,” unnatural, and even disgusting”	
(Buddle et al., 2018a) [11]	Focus group and interviews; 2015-2016	Australia, Meat consumers N= 66, 67% women	How participants respond to social media content from animal welfare activist group	Social media content from animal welfare activist group	Participants responses to animal welfare activism	Consumers indicated that they ignore activist content, or consider the content to be extreme or not reliable (dissonance)	n. a.
(Buddle et al., 2018b) [12]	Focus groups and “mall-intercept” interviews	Australian consumers; (Total N=66, 67% women); 3 Focus groups (9 participants each) Total N=27 + N=39 mall-intercept interviews	Perceptions about sheep and cattle transportation	No info.	Sheep and cattle transportation	(Transport via Truck) A predominant concern was the idea that animals are “crammed”, “shoved”, or forced into trucks, with limited space to move, and that this was “bad treatment”;<br><br>(Transport via ship) Participants expressed disgust and concern for	n. a.

						animal suffering when animals were tightly packed on live export vessels and transported long distances;<br><br>Concerns about animal treatment in receiving countries were generally associated by participants with halal slaughter; Participants used emotive language to describe their perceptions of transport, including “disgust” and “sadness”, and the idea that animals were treated “cruelly” during transport; Animals themselves were also described as appearing	

						"stressed" and "troubled" during transport	
(Busch et al., 2019) [13]	Online survey; 2016	German citizens, N=1019<br><br>(split 1, N=489, 50.3%)<br>(split 2, N=530, 49.8%)	Beliefs in pigs' minds.<br><br>To test how the evaluation of a picture showing a farmed pig is influenced by portrayed attributes	Four pictures showing (Split 1 N=489 and Split 2 N=530) combinations of both 'happy'- or 'unhappy'-looking pig in a pen with slatted floor or straw bedding, respectively.<br><br>In the pretest, participants evaluated seven snapshots on a seven-point semantic differential using 'happy' and 'unhappy' as poles for the evaluation of the pigs	Participants' belief in pigs' minds;<br><br>Evaluations of pigs and pens	Participants' belief in pigs' minds:<br>86% are for sure or probably capable of experiencing emotions;<br><br>For 57.8% pigs are rather conscious of what is happening to them;<br><br>46% agrees about pigs' abilities to solve problems	Pigs are able to think to some extent to solve problems and make decisions about what to do.<br>Yes, for sure 10.9%, Probably yes 35.1%, I am not sure 34.6%, Probably no 14.% No, for sure not 5.3%;<br><br>Pigs are capable of experiencing a range of emotions (e.g. pain, suffering, contentment, maternal affection, aggression…)<br>Yes, for sure 49.6%, Probably yes 36.4%, I am not Sure 11.4%, Probably no 1.8% No, for sure not 0.9%;

							Pigs are conscious and aware of what is happening to them Yes, for sure 20.6%, Probably yes 37.2%, I am not sure 28.1%, Probably no 11.4%, No, for sure not 2.7%;<br><br>Pigs have limited abilities to see cause and effect of an action. Yes, for sure 5.0%, Probably yes 21.8%, I am not sure 39.4%, Probably no 23.7%, No, for sure not. 10.2%<br>Pigs experience emotions less intensely than humans Yes, for sure 3.1%, Probably yes12.5%, I am not sure 42.0% Probably no 27.3%, No, for sure not 15.1%;

							The evaluations of the pens:<br>The straw pen receives more positive values compared to the slatted floor pen on all pictures. Looking at how comfortable the pens are rated by participants, the most positive evaluation is given to the straw pen with the ‘happy’ pig, followed by the other two straw pens (separately and with the ‘unhappy’ pig);<br><br>The slatted floor pen is evaluated always the same and more negatively;<br><br>Regarding the evaluation of the two pens, the slatted floor is perceived more negatively

							than straw bedding. The negative perception of the slatted floor in this study is striking and is in line with the lack of acceptance for this husbandry system by the majority of German citizens
(Cardoso et al., 2017) [14]	Questionnaire, 2017	Brazilian citizens N=296	Explore attitudes of Brazilian urban citizens about specific dairy farming practices	Study 2: The specific practices were presented as follows: (a) the newborn dairy calf is separated from its mother shortly after birth; zero-grazing, on some dairy farms cows are reared inside barns, without access to pasture at anytime when lactating; (b) some male calves are	Participants awareness and acceptability about specific practices in dairy farming	Awareness of the specific practices was low: early cow-calf separation (45%), zero-grazing (32%), culling the newborn male calf (21%), and dehorning/disbudding without pain control (15%);	Rejection of specific dairy practices: Early cow-calf separation (84%), zero-grazing (85%), culling newborn male calf (90%), dehorning without pain control (89%)

				killed immediately after birth because they are not used to produce milk; (c) the horns of young calves are removed without use of any medication to control the pain			
(Christoph-Schulz & Rovers, 2020) [15]	Focus group + online survey; 2017	German citizens, 6 groups (N=8-11 participants each) + N=399, 43.1% women)	Perception of fattening pig husbandry;<br><br>Perception of outdoor access, farm design and flooring type, sources of engagement, space availability, surgical intervention (...)	Respondents from online survey provided with information in case of surgical interventions via the text in brackets ("Usual surgical interventions in pig husbandry are tail docking, teeth grinding and castration	Fattening pig husbandry<br><br>I believe that pigs are intelligent animals<br><br>Interventions on the pig, e.g. shortening of tails, castration or grinding of teeth are performed	(Focus group) The lack of outdoor access in connection with the term "factory farming" was described negatively<br><br>(Online survey) 75% of the respondents think management of fattening pigs need improvement, 70% respondents think saw room,	(Online survey) I believe that pigs are intelligent animals: rather agree (28%); agree (23%); agree at all (26%)<br><br>Interventions on the pig, e.g. shortening of tails, castration or grinding of teeth are performed: Do not agree at all (9%); do not agree (2%) neither nor (21%); rather agree (21%);

				of male piglets”)		sow management and piglet production need improvement	agree (23%); agree at all (20%)<br><br>“The fattening pigs can live out their natural behaviour well” females” (0.117), participants with ages 36 up to 55 (0.141) more often disagreement, less often agreement
(Clark et al., 2019) [16]	Online survey; 2017	Public from five European countries (Finland, Germany, Poland, Spain and the UK) broilers (N=789), layers (N=790) and pigs (N=751) Total N=2330)	Public attitudes towards production diseases	No info.	Intensive animal production for laying hens, broilers and pigs	Three quarters of respondents in all countries were unfamiliar with modern farming practices;<br>Most respondents (51.6% - 88.5%) had not heard anything about production diseases;<br><br>Attitudes citizens towards intensive animal production systems for laying hens, broilers and pigs from 5 EU	Respondents in Germany rated intensive systems more unfavourably than respondents in other countries, especially in relation to them being ‘unpleasant’, ‘bad’ and ‘unethical’

						countries (mean rank on a linear scale: 1 to 5 ± SD)<br>Layers:<br>Unpleasant (1)/pleasant (5) 2.22 ± 1.04<br>Bad (1)/good (5) 2.44 ± 1.05<br>Unethical (1)/ethical (5) 2.17 ± 1.04<br>Broilers:<br>Unpleasant (1)/pleasant (5) 2.09 ± 1.03<br>Bad (1)/good (5) 2.32 ± 1.05<br>Unethical (1)/ethical (5) 2.12 ± 1.06<br>Pigs:<br>Unpleasant (1)/pleasant (5) 2.18 ± 1.06 Bad (1)/good (5) 2.43 ± 1.04<br>Unethical (1)/ethical (5) 2.23 ± 1.10	
(Coleman et al., 2016) [17]	Focus group, telephone	Australian consumers	Public and sheep farmers’	No info.	Measured consumers	Outdoor pens, which are	The more intensive the housing,

	interview, survey	N=490, 257 women	attitudes toward the welfare of lambs in intensive finishing systems		opinions on confinement, limited space to move, movement restrictions: “To what extent do you approve or disapprove of the following practices? To what extent do you agree with a range of possible advantages and disadvantages of lamb finishing systems?”	currently more common than indoor feedlots, were only approved by 20% to 30% of respondents	the more respondents disapproved; 65% of males and 78% of females disapproved of indoor pens; Housing lambs in groups in pens indoors (strongly disapprove - strongly approve) Urban and suburban: 49%, 24%, 18%, 5%, 4% Regional city: 41%, 11%, 34%, 7%, 7% Country town and rural: 55%, 21%, 15%, 5%, 5%<br><br>Welfare issues in feedlots (quite unconcerned - quite concerned) Confinement (to small area) Urban and suburban: 2%, 1%, 5%, 7%, 12%, 20%, 54% Regional city: 2%, 0%, 0%, 14%, 23%, 20%, 41%

							Country town and rural: 4%, 4%, 4%, 5%, 8%, 12%, 63%<br>Limited space to move<br>Urban and suburban: 1%, 1%, 3%, 6%, 11%, 21%, 56%,<br>Regional city: 0%, 0%, 5%, 11%, 14%, 20%, 50%,<br>Country town and rural: 4%, 2%, 4%, 5%, 8%, 11%, 67%<br>Movement restrictions:<br>Urban and suburban: 1%, 1%, 2%, 8%, 12%, 22%, 54%<br>Regional city: 0%, 0%, 5%, 11%, 9%, 25%, 50%<br>Country town and rural: 3%, 2%, 5%, 6%, 7%, 16%, 62%
(Connor & Cowan, 2020) [18]	Online survey, N.D.	UK participants. N=386, 183 women	Consumer evaluation of farm animal mutilations	Each mutilation procedure was explained trying to avoid technical	Tail docking, teeth-clipping and nose-ringing pigs; debeaking hens, debeaking,	Knowledge about mutilations and legislation was assessed with 13 statements (See Table 1 in the	When information is provided, participants viewed mutilations as largely unnecessary both

				terminology. Mutilations included in this study were tail docking, teeth-clipping and nose-ringing pigs; debeaking hens, debeaking, de-toeing and desnooding turkeys; different methods of lamb castration and tail-docking; different methods of cattle castration and nose-ringing as well as dehorning and disbudding cattle and goats	de-toeing and desnooding turkeys; different methods of lamb castration and tail-docking; different methods of cattle castration and nose- ringing as well as dehorning and disbudding cattle and goats	paper from which this data was extracted). Cronbach's α for the scale was 0.69 (N=13). Participants answered the statement ‘Ear-tagging (putting tag/s in the ear/s) farm animals such as lambs, pigs and calves has to be carried out with anaesthetic in the UK’ most often correctly (45.7%). Perception of pain: Participants perceived surgically castration in calves and kids up to 2 months without anaesthetic as the most painful procedure (M=6.86, SD=2.22) followed by	for the animal and the farmer. Furthermore, acceptance of mutilations among participants was low and mainly influenced by peoples' pain perception and perceptions of necessity. Providing the public with Information about pain and necessity has the potential to influence consumer habits and decision making regarding purchasing animal products.<br><br>Participants answered between 0 and 9 knowledge statements correctly with a mean of 2.82 (SD=2.41) with no difference between male (M=3.08, SD=2.51, n=123) and

						surgical castration in lambs with a sharp knife whereby the scrotum is cut open and testes are pulled out Less 3 months old (M=6.84, SD=2.50) and crushing of the spermatic cord in calves and kids up to 2 months of age without anaesthetic (M=6.79, SD=2.26). Dehorning cattle and goats by using a saw was perceived to be the least painful procedure (M=4.35, 2.27) followed by disbudding calves and kids with a hot iron (M=4.48, SD=2.77) Participants perceived the	female's (M=2.65, SD=2.35, n=183) knowledge about mutilations (t304=1.54, p=0.125). There were significant differences between people who work with animals (M=4.25, SD=2.00, n=36) and participants who do not work with animals (M=2.63, SD=2.40, n=270, t49.4=4.43, p < 0.001)

						procedures carried out on poultry to be the most painful ones (M=6.42, SD=2.09) followed by procedures carried out in pigs (M=6.28, SD=1.08), cattle/goats (M=5.79, SD=1.96) and lambs (M=5.76, SD=2.01). Acceptability of procedures: Participants rated tail docking in lambs (M=2.28, SD=1.68) as the most necessary procedure to be carried out for the animals followed by teeth clipping in pig (M=2.25, SD=1.62) and disbudding kids and claves (M=2.24, SD=1.65)	

(de Haas et al., 2021) [19]	Survey; 2020	Dutch public, N=259, 143 women	To assess the awareness and acceptance of culling male chicks, and preference for alternatives	Information about culling day old chicks was provided in different stages of the questionnaire	Awareness regarding culling; Acceptance regarding culling	52% knew about the practice of culling;<br><br>78.8% disagree with culling	When provided with information (Q44) highly educated women strongly disagree (45.5%) and disagree (32.2%) with male chicks culling
(de Jonge & van Trijp, 2013) [20]	Survey; 2012	Dutch participants, N=209, 59% women	To what extent different farm management practices influence the perceived animal friendliness of broiler production systems	Respondents were asked to what extent they perceived one broiler system profile description (A) as more or less animal friendly than another broiler system profile description (B) graded paired comparisons between 2 production systems that were described on the basis of 7 attributes	Outdoor access; Stocking density; Breed/growth rate; Enrichment; Transport; Slaughter	Stocking density came out as the second most salient attribute; broiler production systems were perceived as more animal friendly when individual animals had more space in the barn (i.e., when stocking density was low);<br><br>Transport duration and breed selection for growth rate came out as significant but less salient broiler system attributes in the	Consumers perceived conventional broiler systems (mean = −0.71; SD = 0.32) to be less animal friendly than organic broiler production systems [M = 1.61; SD = 0.41; t(190) = −79.6; P < 0.001];<br><br>Familiarity with farming, subjective knowledge, fairness beliefs, care about animals, and gender showed significant relationships with the relative importance attached to production

						perception of animal welfare	practices
(Dowsett et al., 2018) [21]	Online survey; N.D.	Australian meat eaters, N=460, 274 women	To determine whether exposing participants to the meat-animal connection would alter their affect, meat attachment, and attitude towards animals	Audio-visual footage demonstrating the intelligence of a lamb by its ability to open two fastened farm gates	Experimental manipulation: exposure to the animal origin of meat or learning about the nutritional benefits of meat	Exposure to one's moral inconsistencies may trigger greater attachment to meat and more defensive justifications for meat eating, leading to increased commitment to meat consumption	Female participants experienced increased negative affect, decreased meat attachment, and greater concern for animals than male participants
(Estévez-Moreno et al., 2021) [22]	Cross-cultural survey	Mexican and Spanish consumers N=833, 55.9% women; N=1455, 53.5% women	How meat consumers perceive farm animal welfare	No info.	(6 questions from a list of 15. With Likert scale) “Do you believe that farm animals should be free of fear and stress?” “Do you believe that farm animals can feel pain and Suffering?” “Do you believe that farm		Concerning animal welfare: spanish women scored than men (8.7 vs. 8). For Mexico, a similar trend (8.4 women vs. 7.9 men); Spaniards of rural and urban origins gave a slightly higher overall score than Mexicans; Spanish and Mexican urban consumers scored

					animals can feel positive or negative emotions?” “Do you believe that the living conditions of farm animals have improved in the last 10 years?” “Would you like to be informed about the living conditions of farm animals you eat?” “Do you think that the welfare and protection of farm animals in our country should be improved?”		higher than rural ones; More educated Mexicans were more concerned about animal welfare; People aged between 31 and 45 years old assign the highest value for animal welfare in each country; For the age range between 18 and 30 years old, Spaniards attach greater importance to animal welfare than Mexicans (8.4 vs. 8.1); The oldest consumers (46 to 60 years, >60 years) in both Countries assigned a lower grade to the importance of animal welfare compared to those between 30 and 45 yrs;

							Spaniards compared to Mexicans, give more importance to aspects such as: i) the health conditions of farm animals, ii) that farm animals should express normal patterns of behaviour, iii) that farm animals should be free from fear and stress
(Estévez-Moreno et al., 2022) [23]	Online survey; 2018 - 2019	Argentina, Chile, Colombia, Ecuador, Peru and Bolivia. Cluster 1 (ethically concerned and least informed) N=1323) Cluster 2<br><br>(ethically concerned and	Consumer attitudes towards farm animal welfare	No info.	1) Do you believe that farm animals should be free from fear and distress?<br>2) Do you believe that farm animals feel pain?<br>3) Do you believe that farm animals feel positive or negative emotions?	1) 5.0/5 (5= definitely yes) (Median Interaquartile range)<br>2) 5.0/5 (5= definitely yes) (Median Interaquartile range)<br>3) 5.0/5 (5= definitely yes) (Median Interaquartile range)	n.a.

		information intermediate)<br><br>N=2852 participants, 60% women Argentina (26.8%), Chile (20.4%) and Colombia (18.8%)					
(Fredriksen et al., 2011) [24]	Focus group and web-interview study, 2008	Norwegian consumers (Group 1, 5 women; Group 2, 5 women; Group 3; 5 women) + N=1013 participants from internet-based study	Consumers attitudes towards surgical castration of piglets and alternatives	Statements used to inform the consumers in the focus group study (F) and the internet study (I) about current castration practice and possible alternatives. All statements were presented all participants in the respective studies	Surgical castration of piglets	Focus groups consumers did not seem to be concerned about animal welfare during the purchasing situation.<br>The knowledge of castration practice was low. 77% of participants (from focus group study) found surgical castration without anaesthesia not acceptable	Majority of the consumers (60%) were not aware that Norwegian male piglets are routinely castrated. The knowledge was higher in the rural areas (57%), and lowest among younger people (18% in the group 15–24 years)

(Frewer et al., 2005) [25]	Online questionnaire, N.D.	Dutch consumers, N=500	Consumer attitudes to the development of optimal animal husbandry practices for pigs and fish	No info.	Perceptions of farmed pigs, and the other for farmed fish; Welfare Items (13): “To what extent do you agree / think..”	The extent consumers perceive that fish or pigs experience emotions: (Pigs Mean SD: 2.99 (1.29) 111 pleasure; 4.47 (1.10) 112 pain; 4.59 (1.31) 108 boredom; 4.60 (1.17) 93 fear; 4.83 (1.10) 84 stress) (Fish Mean SD: 3.19 (1.29) 193 pleasure; 3.92 (1.30) 183 pain; 3.73 (1.50) 200 boredom; 4.03 (1.27) 188 fear; 4.43 (1.31) 158 stress	n.a.
(Fuseini & Knowles, 2020) [26]	Questionnaire; 2017-2018	U.K. Halal consumers, N=250, 32.1% women	Halal meat consumers understanding a preferred method of Halal slaughter	No info.	Understanding of pre-stunned Or post-cut stunned during Halal slaughter;<br><br>Preferred method of Halal Slaughter	(Agreebility) ‘Stunning of meat animals prior to slaughter has been shown to reduce the pain associated with slaughter’. 31% Yes	Male respondents, 70.7% indicated preference for meat from animals slaughtered without stunning over meat from pre-stunned

						69% No<br><br>Understanding of pre-stunned Or post-cut stunned during Halal slaughter: 78.9% understand;<br><br>Preferred method of Halal Slaughter: Pre-stunned slaughter on condition that the animal was alive at the point its neck was cut 14.2%;<br>Slaughter without stunning 69.9%<br>Post-cut stunned slaughter (this is where a live animal is slaughtered followed by stunning) 0.4%	animals.<br>The preference for method of slaughter by female respondents was similar at 67.9%
(Fuseini et al., 2017) [27]	(Online) survey; 2015-2016	U.K. Islamic scholars and Halal consumers	Perception and acceptability of pre-slaughter	No info.	Stunning of meat animals prior to slaughter has been shown	(Scholars' survey)<br>Animal must be alive at the point	

		N=66 5% women<br>N=314 27% women	and post-slaughter stunning for Halal		to reduce the pain associated with slaughter;<br><br>(Agreeability)<br>Some methods of stunning have been shown to be reversible, that is, such methods do not lead to the death of animals prior to slaughter (bleeding-out).<br>(Awareness)<br>If an animal is stunned and then slaughtered by a Muslim and the method of stunning does not injure or result in the death of that animal before slaughter, and blood loss is not adversely affected, would you regard this meat as Halal?	of slaughter 98%<br>Animal must be healthy and not injured 38%<br><br>The slaughterman bleeding the animal must be a Muslim 80%<br><br>Knife sharpness is important 51%<br>Appropriate blood vessels must be severed 51%<br><br>The name of God must be recited before slaughter 21%<br><br>Stunning of meat animals prior to slaughter has been shown to reduce the pain associated with slaughter.<br>(Agreeability)<br>Yes 31%<br>No 69%	

					If an animal is slaughtered whilst it is alive, followed immediately with stunning, would you regard this practice as Halal?<br><br>If an animal is slaughtered without any form of stunning, which of the following do you consider to apply?<br>The animal will feel reduced pain because the knife acts as a stun.<br>The animal will feel pain.	If an animal is stunned and then slaughtered by a Muslim and the method of stunning does not injure or lead to the death of that animal before slaughter (bleed-out)<br>and does not affect blood loss, would you regard this meat as Halal? Yes 95%<br>No 5%<br><br>If an animal is slaughtered without any form of stunning, which of the following<br>do you consider to apply?<br>The animal will feel reduced pain because the knife acts as a stun 78%<br>The animal will feel pain 22%	

						If an animal is slaughtered whilst it is alive, followed immediately with stunning,<br>would you regard this practice as Halal?<br>Yes 88% No 12%<br><br>(Consumers' survey)<br>Animal must be alive at the point of slaughter 95%<br><br>The slaughterman bleeding the animal must be a Muslim 72%<br><br>Stunning of meat animals prior to slaughter has been shown to reduce the pain associated with slaughter. Do you agree with this statement?<br>Yes 42% No 58%	

						If an animal is stunned and then slaughtered by a Muslim and the method of stunning does not injure or lead to the death of that animal before slaughter (bleed-out) and does not affect blood loss, would you regard this meat as Halal?<br>Yes 53% No 32% Not sure 15%<br><br>If you answered No to the preceding question, please state why?<br>Pre-slaughter stunning is against Islamic law 57%<br>Stunning is painful/can injure animals 14%<br>Stunning may result in the death of animals before slaughter 14%	

						Would avoid stunned meat because they have little or no knowledge about it 11%<br>Stunning obstructs bleed-out 3%<br><br>If an animal is slaughtered without any form of stunning, do you consider any of the following to apply?<br>The animal will feel reduced pain because the knife acts as a stun 60%<br>The animal will feel pain 33%<br>There is improved blood loss in comparison with stunned animals 20%<br><br>If an animal is slaughtered whilst it is alive,	

						followed immediately with stunning, would you regard this practice as Halal?<br>Yes 69% No 31%	
(Gremmen et al., 2018) [28]	Online survey; 2015	Dutch respondents, N=1022, 533 women	How do Dutch society perceive the killing of day-old male chicks in the egg sector	No info.	Killing of day-old male chicks (awareness) (Multiple choice options of the online survey)<br>3. What is your opinion about killing day-old male chicks?	55% of respondents are aware of the killing of day-old male chick;<br>30% of respondents think that it is a good practice or do not have problems with it	n.a.
(Hartmann & Siegrist, 2020) [29]	Online survey; 2015	German participants, N=973, 51% women	To determine whether meat-eating justification strategies - unapologetic (pro-meat, denial, hierarchical justification, religious justification, health justification,	No info.	Participants' assessments of how morally justifiable they perceive the following meat production methods to be:<br>Foi gras;<br>Alive boiled lobster;<br>Bull beef from intensive production;	Most of the investigated production methods were perceived as morally not justified.<br>Conventional meat production methods received the most negative evaluations.<br>These negative attitudes towards	The least morally justifiable meat was foie gras, with a mean value of 7.31 on a scale with a maximum value of 8.<br>Only the three meat production methods 'beef from cattle from organic farms', 'free-range chicken' and

			human destiny and slaughter justification) and apologetic (dichotomization , dissociation And avoidance) - not only exert an effect on meat consumption frequency but also on the moral evaluations of diverse meat production systems		Veal from conventional production; Beef from cattle in tie-stalls; Meat from broilers from intensive production systems; Pork meat from pigs from intensive commercial units; Hunted wild animals (e.g. deer, wild boar, duck); Free-range chicken; Beef from cattle from organic farms; Moral justifiability of various meat production systems;	meat production systems seem to have a very limited effect on meat consumption, and even though people claim to be concerned about welfare issues, the majority does not consider these aspects when consuming or buying meat; These justifications may then be used by consumers to avoid/reduce the negative emotions evoked by gap between ethical convictions and consumer behaviour. Meat-eating justification strategies might also influence the moral justifiability of meat production	'hunted wild animals' were perceived as relatively morally justifiable (with values below the theoretical midpoint of 4.5 on the scale). All the justification strategies except dichotomization were significantly correlated with moral justifiability of the three production systems. People who dissociated animals and food and who avoided negative thoughts about animal husbandry systems and slaughter evaluated the meat production systems more negatively. Unapologetic strategies were associated with a more positive moral evaluation of the meat production

						systems, which explains the observed negative correlation between moral evaluation and unapologetic justification strategies. The endorsement of unapologetic meat-eating justification strategies, such as emphasising the taste of meat, denial of animal suffering and asserting that animals are lower than humans in the hierarchy, seem to be a driver for resistance. They were linked to more positive moral evaluation of meat production systems, higher meat consumption and	systems. Males were less morally concerned about conventional meat production compared with females (β=−0.11, $p < .001$). Both the apologetic (β=0.15, $p <.001$) and unapologetic (β=−0.43, $p <.001$) justification strategies were statistically significant predictors. The participants endorsed unapologetic strategies to justify their meat consumption, the more likely they were to consider conventional meat production systems as morally justified. The more the participants endorsed the apologetic strategies to justify their meat

						lower WTS meat. The unapologetic justification strategy of health justification was one of the strongest correlates of the frequency of consuming fresh meat and processed meat, and was also associated with lower WTS meat with alternatives. Men are more likely to use unapologetic strategies, while women would rather use indirect, apologetic strategies by dissociating the animal from the meat on their plate and trying to avoid thinking about it. The unapologetic justification strategy of health	consumption, the more likely they were to evaluate conventional meat production systems as morally not justified. All unapologetic strategies, dichotomization and avoidance were positively correlated with meat consumption frequency. All strategies except for dissociation and avoidance were negatively correlated with WTS meat. The highest correlation coefficients were observed for pro-meat ($r=-0.51$, $p < .001$), hierarchical justification ($r=-0.41$, $p < .001$), health justification ($r=-0.39$, $p < .001$) and slaughter justification

						justification was one of the strongest correlates of the frequency of consuming meat and was also associated with lower WTS meat with alternatives	(r=−0.41, p < .001)
(Heid & Hamm, 2012) [30]	Focus group; 2009	German organic consumers, N=89, 55 women	Organic consumers' attitudes towards piglet castration without pain relief and examines which three alternatives are important to consumers of organic products	At the beginning of the group discussion, the moderator informed consumers that male piglets that are reared for meat production are castrated (referring to the questionnaire). At that point, it was not mentioned that castration is usually performed without	Piglet castration without pain relief:<br>Group 1 Minimal information<br><br>Group 2 Full information<br><br>Group 3 Full information incl. 'hormone'<br>Group 4 Minimal information<br>Group 5Full information<br>Group 6 Full information<br>Group 7 Minimal information<br>Group 8 Full information<br>Group 9 Full	A majority of the consumers (60%) were not aware that male piglets are routinely castrated	The proportion of participants who were not aware of piglet castration ranged between 17% (group 5) and 73% (group 9) in the different focus groups, with in total 54% of participants not knowing that male piglets are castrated for fattening;<br>The knowledge was higher in the rural areas (57%), and lowest among younger people (18% in the group 15–24 years)<br><br>The fact that

				anaesthesia. After a short discussion about the possible reasons for castration, a brief presentation was given, with standardised information on piglet castration and the alternative methods. Information provision varied between each of the three focus groups per region in order to examine the influence of different levels of information	information incl.‘hormone’		castration is usually performed without anaesthesia was widely unknown, even among those participants who claimed to know about castration of male piglets. The few participants that were aware of castration without anaesthesia mainly obtained their knowledge from recent media reports. Several participants expressed surprise and disappointment, especially with reference to organic farming, when they were informed about the usual practice; Castration without anaesthesia was regarded as unnecessary cruelty to animals

(Herrewijn et al., 2021) [31]	Experimental study; 2019	Belgian participants, N=84, 44 women	Does speciesism act as a mediating variable in explaining the impact of a VR experience on empathic concern	Short 360◦ Documentary – “iAnimal: Pig Farms in 360 Degrees” - depicting the life cycle of factory farmed pigs (from their lives on the farm to their death in the slaughterhouse) experienced in a VR format versus in a regular video format Documentary depicts forcefully inseminated and suckling sows; non-sedated castration of male piglets; shows the pigs in cramped and unhygienic crates or spaces, with pathological and aggressive	(Likert scale) I am willing to prepare less meat during the week’, ‘I am willing to prepare smaller portions of meat for each meal’; ‘How strongly did you have the feeling to be present in the meat preparation company during the exposure of the images?‘, ‘How strongly did you have the feeling that you were part of the company during the exposure to the images?‘	The experimental study in which respondents were exposed to Animal Equality’s 360◦ documentary in a VR versus video format showed no significant total effect on participants’ intention to reduce meat consumption; Study demonstrate VR (versus video) experience can have on participants’ sense of feeling present in the slaughterhouse - that increased the participants’ empathic concern for the animals, which subsequently increased their intentions to reduce animal food intake.	VR (vs. video) did have a direct positive effect on presence, a1 = 0.60, β = 0.45, SE = 0.29, t (82) = 2.10, 95% CI [0.03, 1.17], p = .039. Presence had a positive effect on empathic concern, d21 = 0.25, β = 0.37, SE = 0.07, t (81) = 3.54, 95% CI [0.11, 0.39], p < .001, and likewise, VR (vs. video) had an indirect effect on empathic concern via presence, a1d21 = 0.05, BootSE = 0.04, 95% BootCI [<0.01, 0.38]; VR (vs. video) also had a direct, negative effect on empathic concern when the effect of presence was statistically controlled for, a2 = - 0.42, β = -0.47,

				behaviour (and sometimes death); they are brought to slaughterhouse where they are (not always effectively) stunned by Electrocution; hoisted on a rail hanging upside down and then killed by cutting their throat and letting them exsanguinate At the end of the documentary, a message of Animal Equality reads: 'You don't have to see the world through the eyes of a pig to recognize the cruelty and suffering, but		Speciesism can play a counterproductive role in the persuasive impact of VR (compared to video) messages Involving morally salient topics (the horrible realities of the meat industry) when trying to change people's behaviour; Study provides mixed evidence for the effectiveness of Animal Equality's VR (versus video) outreach to promote meat reduction intentions. On the one hand, VR (versus video) might increase consumer intentions to eat less meat because of its	SE = 0.19, t (81) = -2.23, 95% CI [-0.80, -0.05], p = .029, suggesting that another mediating variable (speciesism) counteracted the positive mediating effect of presence between medium format and empathic concern; VR (vs. video) positively predicted intentions to reduce meat consumption via presence and empathic concern in serial, a1d21b2 = 0.05, β = 0.04, BootSE = 0.04, 95% BootCI [0.004, 0.31]; VR (vs. video) also had a negative, counteracting effect on meat reduction intentions because of its negative effect on empathic concern, a2b2 =- 0.13, β =

				you can see an end to this, please leave meat off your plate.’		positive effect on empathic concern via presence. On the other hand, VR might negatively affect empathic concern by evoking more speciesist attitudes	-0.12, BootSE = 0.08; 95% BootCI [-0.32, - 0.01], which could explain why we did not find a total effect of VR (vs. video) on participants’ meat reduction intentions, R2 = 3.4%, c = 0.07, SE = 0.23, t (82) = 0.31, 95% CI [-0.38, 0.53], p = .755. Presence only had a positive effect on meat Reduction intentions via empathic concern and did not affect meat Reduction intentions directly, b1 = 0.14, β = 0.18, SE = 0.09, t (80) = 1.56, 95% CI [-0.04, 0.32], p = .122, so the indirect effect of VR (vs.video) on meat reduction intentions via presence was

							also not significant, a1b1 = 0.08, β = 0.08, BootSE = 0.08, 95% BootCI [0.07, 0.27]
(Hötzel et al., 2017) [32]	Survey; exploratory; N.D.	Brazilian citizens (Total N=400) Group A (N=200, 54% women); Group B N=200, 50%)	Assess the influence of provision of information on lay citizens' opinions regarding zero-grazing and cow-calf separation and to explore the awareness and opinions about these practices	Participants were presented short scenarios with information on the primary production factors and welfare concerns for and against zero-grazing (n = 200) or cow-calf separation (n = 200). Participants were asked to state their position (reject, indifferent, or support), and to provide the reason(s) justifying their position. Immediately following,	Zero-grazing; Cow-calf separation	Only 31% respondents were aware of zero-grazing and 33% of cow-calf separation;<br><br>Animal Welfare. Aspects of animal life included explicit mention of animal welfare, freedom to move, animal comfort, health, stress, or ability to perform Natural behaviours;<br>Many participants described keeping young animals with their mother or cows on pasture as the norm, and alternatives as aberrant	Influence of awareness and Provision of Information on Support for Practices:<br>Support for both zero-grazing ($P < 0.04$) and cow calf separation ($P < 0.001$) was influenced by provision of information.<br>Reasons Given in Support Of or Against Zero-Grazing and Early Cow-Calf Separation<br>50% naturalness<br>45% animal welfare;<br>Reasons Given in Support Of or Against Zero-Grazing:<br>Naturalness. The

				participants were provided a short statement describing either zero-grazing or cow-calf separation, depending on what question they responded to in the first part. Participants were then asked to state their position (reject, indifferent, or support), and to provide the reason(s) justifying their position			word “natural” appeared in 72 of the 172 reasons presented and referred primarily to the living environment provided for the animals; Percentage of participants who either rejected, were indifferent, or supported zero-grazing systems for dairy cows or early cow-calf separation (Information capsule) Zero-grazing<br>Reject 86.1%<br>Indifferent 12.2%<br>Support 1.7%<br>Cow-calf separation<br>Reject 69.2%<br>Indifferent 17.3%<br>Support 13.5%<br>(Short statement)<br>Zero-grazing<br>Reject 75.7%<br>Indifferent 20.5%<br>Support 3.8%<br>Cow-calf separation<br>Reject 61.7%

							Indifferent 32.8%<br>Support 5.6%<br>Brazilians largely living in urban environments and with little or no association with dairy production, were generally unaware that cows are<br>separated from their calf at birth and that an increasing number of cows are reared in zero-grazing systems
(Hötzel et al., 2020) [33]	Survey; 2017	Brazilian citizens;<br>Two surveys (Sv1 N=441 Sv2 N=768) (Total N=1209)	Attitudes of Brazilians towards different piglet castration methods (surgical castration without or with pain control and immunocastration	In Sv1 and Sv2 participants were informed that Brazilian legislation states that all slaughtered male pigs must be castrated and were informed about the procedures	Sv1 included 37 closed questions and 1 open question;<br><br>Sv2 included a 27 closed questions and 1 open question	Pig producers tail dock piglets to avoid them from being bitten by others in<br>their group Sv1 26% Sv2 17%<br><br>Male pigs used in meat production undergo castration or	Sv1 (N=441):<br>Surgical castration Acceptable 15; Unacceptable 73; Neutral 12<br>Immunocastration Acceptable 53; Unacceptable 27; Neutral 20<br>Surgical castration with pain control Acceptable 63;

				and motifs about castration types		immunocastratio Sv1 30% Sv2 33%<br><br>Participants in the two surveys were opposed to piglets' surgical castration without pain control.<br>The main reason for this position was the perception that the practice causes unnecessary pain and stress to animals and is therefore ethically unacceptable	Unacceptable 22; Neutral 15<br>Surgical castration Acceptable 18; Unacceptable 67; Neutral 15;<br><br>Sv2 (N=768)<br>Immunocastration Acceptable 56; Unacceptable 25; Neutral 19<br>Entire male Acceptable 52; Unacceptable 32; Neutral 16<br><br>In Sv2, attitudes were influenced by the method of dealing with boar taint (surgical castration without pain control = 2.08; immunocastration = 3.38; entire male 3.28; SEM 0.08; p = 0.001) and sex of participant (female = 2.72; male = 3.11; SEM 0.06; p = 0.001).<br>Interactions

							between methods and sex were tested and excluded from the model, as they were not significant ($p > 0.2$). Attitudes towards immunocastration and entire males did not di er ($p = 0.74$) and both were higher than towards surgical castration without pain control ($p = 0.001$)
(Jalil et al., 2018) [34]	Quantitative questionnaire; December 2015 and January 2016	Malaysia and Australia; 563 Australian and 735 Malaysian. (51% female) Malaysian (80% muslims); Australian (46% none, 45%	Beliefs about animal welfare, Islam, and halal products	A definition of animal welfare was given: "an animal is in a good state of welfare if (as indicated by scientific evidence) it is healthy, comfortable, well nourished, safe, able to express innate behaviour, and	In halal slaughter, the humane and respectful treatment of animals is: (not important (1) to very important (5))		1) Religion: Muslims: 4.61; Christians 3.82; None: 3.84<br><br>2) Education: Undergraduate degree: 4.40; School leavers: 4.05; Certificate diploma: 4.17; Post-graduate degree: 4.16<br><br>3) Income:

		Christian)		if it is not suffering from unpleasant states such as pain, fear, and distress”			<RM10,000/ AUD20,000: 4.36; RM10,000-29,000/ AUD20,000-39,000: 3.95 RM20,000-49,000/ AUD40,000-59,000: 4.19 RM50,000-69,000/ AUD60,000-79,000: 4.10 >RM70,000/ AUD80,000: 4.03<br><br>Reasons for purchasing or avoiding halal meat: animal welfare reasons were the most common reason for avoiding halal meat.
(Krystallis et al., 2009) [35]	Online survey; N.D.	Individuals from four European countries, (Belgium, Denmark, Poland, Germany (Total N=1931,	To map citizen attitudes towards pig meat production systems	15 verbal descriptions of various pig farms	Attitude towards animal welfare was measured with six items from Kendall et al. (2006),	“Housing and floor type” and “efforts to protect soil, air and water at the farm” had the strongest influence on the respondents' evaluation of pig production	Cluster 1 (15.4%) preferred certain attributes of more intensive types of farming, such as medium farm size (about 400 sows), litter type; Cluster 2 (53.7% of

		51.3% women)				systems; lowest evaluations resulted from “slatted floor” type and the “minimum effort” to protect soil, air and water, followed by quality as “demanded by key customers”, a stocking density with “more than 800 sows”	the sample) prefer attributes of extensive types of farming, such as small farm size (less than 100 sows); Cluster 3 (11.2% of the sample) puts the strongest emphasis on the type of housing and floor;<br><br>Attitude towards animal welfare:<br>- “Animal agriculture raises serious ethical questions about the treatment of animals.”<br>(Mean) Cluster 1: 4.45; Cluster 2: 4.38; Cluster 3: 4.96; Cluster 4: 5.25
(Kühl et al., 2019) [36]	Online survey, 2016	German citizens, N=358, 51.7% women	To analyse citizens’ evaluation and acceptance of the four most common	All participants were provided with four sets of four pictures of each	Indicate the overall acceptance of the four husbandry systems	The results show, that the publics’ negative evaluation of indoor housing systems is	In general, the statement animals can live according to their natural behaviour was evaluated most

			husbandry systems for dairy cows in Northwestern Europe	husbandry system were shown (two from the exterior and two from the interior view). These were accompanied by brief details about the particularities of each system, as well as the differences between them (The pictures used in the questionnaire were chosen by three dairy farmers who were asked to pick the pictures which represented CLH systems best and showed the housing from different points	(Likert-scale)	striking;<br>The assumption, that a more natural environment in confinement systems can increase social acceptance, seems only true for a small extent, when including daylight and fresh air in CLH systems.<br>The results presented in Section 4.1 suggest that increased access to fresh air and daylight is not sufficient to increase the acceptance of keeping dairy cows indoors. This is further supported by the evaluation of the pictures of CLH systems	negatively for the three systems WLH, CLH and CLH plus paddock. Only 4.2% of participants either accepted or totally accepted the WLH system.<br>Furthermore, the acceptance of CLH was still low (16.5%), but loose housing with a paddock had a relatively high acceptance rate (54.9%). In contrast, nearly all respondents (96.1%) accepted loose housing with access to pasture in summer.<br>Pictures of CLH systems were evaluated either negatively or very negatively by about 40% of the respondents.<br>The reasons for choosing a picture

				of view)			as the most negative also differed significantly between the pictures ($\chi 2=165.10$; df=39; $P \leq 0.001$; V=0.406; $P \leq 0.001$). For example,<br>reason for choosing picture A as most negative was no freedom of movement, whereas respondents who chose picture B as the most negative cited the unsuitable flooring;<br>24% of all respondents indicated, that they viewed none of the pictures of CLH systems as positive. The reasons given included the lack of outdoor access for cows, their restricted freedom of movement and that this type of

							husbandry system was not species-appropriate
(Kupsala et al., 2014) [37]	Online survey; 2010	Finish citizens; N=1824, 56.1% women	Public perceptions of animal mind	No info.	Mental capacities of animals (several species)	(Pain %)<br>Dog 97.3<br>Cow 96.0<br>Pig 95.3<br>Wolf 92.1<br>Elk 94.0<br>Chicken 91.4<br><br>(Pleasure %)<br>Dog 93.1<br>Cow 89.4<br>Pig 87.6<br>Wolf 77.7<br>Elk 77.3<br>Chicken 69.6<br>(Sadness%)<br>Dog 87.1<br>Cow 69.7<br>Pig 63.6<br>Wolf 62.4<br>Elk 57.0<br>Chicken 37.8<br>(Affection %)<br>Dog 96.6<br>Cow 87.6<br>Pig 79.5<br>Wolf 63.4<br>Elk 56.7<br>Chicken 48.2<br>(Anger %)	Older people attribute fewer mental capacities to dogs, pigs, wolves and elk than younger people; Having a companion animal is associated with a greater belief in the mental capacities of all animals.<br>A negative evaluation of the farm animal welfare situation is related to greater attribution of mental capacities to pigs, wolves, elk and chickens. Animal instrumentalization is connected to reduced belief in the mental capacities of all animals;<br>Chickens are given noticeably fewer mental capacities

						Dog 78.6<br>Cow 59.5<br>Pig 55.7<br>Wolf 66.1<br>Elk 57.1<br>Chicken 39.7<br>(Remembering conspecifics %)<br>Dog 92.8<br>Cow 85.7<br>Pig 80.6<br>Wolf 85.0<br>Elk 84.3<br>Chicken 62.7<br>(Ability to think %)<br>Dog 80.3<br>Cow 66.4<br>Pig 65.5<br>Wolf 66.4<br>Elk 61.6<br>Chicken 41.2<br>(Understanding death%)<br>Dog 77.7<br>Cow 61.1<br>Pig 56.6<br>Wolf 67.7<br>Elk 62.2<br>Chicken 32.4<br><br>(Belief in animal mind scales Mean):	than mammals

						Dog 7.04<br>Cow 6.15<br>Pig 5.84<br>Wolf 5.81<br>Elk 5.50<br>Chicken 4.23	
(Kupsala et al., 2015) [38]	Online survey; 2010	Finish citizens, N=1890, 56% women	To investigate citizen attitudes to farm animals in Finland	No info.	Farm Animal Welfare Evaluation; Trust in Prevalent Animal Production; Behavioral freedom; Valuing Animal Life<br><br>Respondents were asked to evaluate Farm Animal Welfare<br><br>Behavioral Freedom Scale (‘‘All farm animals should be given the opportunity to carry out species-specific behavior’’, ‘‘Farm animals	Farm Animal Welfare Evaluation (M=3.37; SD=0.78):<br>Trust in Prevalent Animal Production (M=3.14; SD=0.78):<br>Behavioral Freedom Scale: M=4.19; SD=0.67<br>Valuing animal life (M=3.02; SD=1.03)	Older people and people with farming backgrounds express more trust in prevailing animal production, while women, urban residents and people with companion animals express less trust in animal welfare in prevalent farming.

					can become depressed in inadequately enriched living conditions'', ''Calves should have enough space to run and play with each other'', ''Calves should not be separated from their mothers right after their birth'' and ''Keeping laying hens in cages should be banned for animal welfare reasons''<br><br>Valuing Animal Life Scale (''Young animals (e.g. calves, fattening pigs and pullets) should not be killed for food production reasons'', ''Dairy cows		

					and sows should not be killed solely for reasons of low productivity'', and ''Meat products should be consumed in low quantities because their production requires animals to be slaughtered''		
(Lama et al., 2017) [39]	Personal interviews; 2015	Mexican consumers; N=84, 55.9% women	Consumers' perceptions and attitudes towards farm animal welfare	No info.	Do you believe that? (several issues concerning animal welfare) (10-point Likert scale)	(Means score)<br>- The animals on the farms feel pain 4.64<br>- The animals on the farms should be free of fear and stress 4.44<br>- The animals on the farms feel positive or negative emotions 4.31<br>- The farm's animals should be able of expressing the natural behaviour of their species	Even though the overall average value of the sample was high (8.1), women gave more importance to farm animal welfare than men (U of Mann-Whitney test, P = 0.003), as did the more highly educated (Kruskal-Wallis test, P = 0.000). In the global sample, the effect of age and origin was not significant

							(Mann-Whitney And Kruskal-Wallis, P ≥ 0.05)
(Leach et al., 2022) [40]	Online survey; n/d	Meat eaters U.K. N=311, 179 women U.S., N=94, 43 women U.K., N=335, 217 women	Study 1: Test the commitment in eating meat and the tendency to avoid information about animal minds<br>Study 2a and 2b: examined people's exposure-motivation for information about pigs and about dogs; test whether meat commitment is strategic in that it might specifically be associated with avoidance of information about animals that challenge	Study 1: Participants provided with descriptions of food animals that are reared and slaughtered; and as sentience as "animals' with capacity to think, feel, experience pain and suffering<br>Study 2a and 2b: Participants presented with background information about pigs and dogs, including that they are slaughtered for food in the US (pigs) and southern China	Variable to be tested: pigs, cows, chickens' capacity to think, feel, experience pain and suffering	A commitment to eating meat was associated with lower exposure time to information about food-animal minds, β = - 0.14, SE = 0.06, 95% CI [-0.26, - 0.02], p = .025, and this relationship was significantly larger than for nonfood-animals, β = 0.19, SE = 0.08, 95% CI [0.03, 0.36], p = .024.	Study 1: Those who were more committed to eating meat were more likely to want to avoid information about food animals' sentience, r(309) = .26, 95% CI [0.15, 0.36], p < .001<br>Study 2a: Those who were more (vs. less) committed to eating meat were less likely to be interested in articles about intelligent pigs, β = -0.15, SE = 0.05, 95% CI [- 0.24, - 0.06], p = .001.<br>No evidence to suggest that meat commitment predicted avoidance of articles about intelligent dogs, β =

			people's dietary choices	(dogs).			- 0.01, SE = 0.05, 95% CI [- 0.10, 0.08], p = .887
(Lemos Teixeira et al., 2018) [41]	(Online) Survey	Chilean participants<br><br>Survey 1 N=400, 52.5% women<br><br>Survey 2 N=100x4 = 57% women, 52% women, 55% women, 49% women (N=400) (Total=800)	Opinion towards castration without anaesthesia;<br><br>Lack of access to pasture in cattle	Survey 1: Participants were given Information in regards to surgical castration without anaesthesia to male cattle pointing pros as cons for animals and production systems;<br><br>Participants were given information (pros and cons for animals and production systems) in regards to cattle fattening;<br><br>Survey 2 four treatment groups of 100 participants	Surgical castration without anaesthesia<br><br>Lack of access to pasture for livestock in confined systems	The majority of participants from the two surveys were opposed to surgical castration without anaesthesia and lack of access to pasture for livestock in confined systems<br><br>Survey 1: 79% participants answered that they were aware of surgical castration without anaesthesia;<br><br>83% aware of lack of access to pasture for livestock in confined systems are common management practices in beef production systems in Chile	Survey 1: 62.5% were opposed to surgical castration without anaesthesia; 20.5% were indifferent and 17% supported the practice;<br><br>59.3% were opposed to lack of access to pasture for livestock in confined systems; 20.5% were indifferent and 20.2% supported this management practice;<br><br>Survey 2: 79.5% of participants were opposed to surgical castration without anaesthesia; 15% were indifferent and 5.5% supported

				each.<br>Participants received one of four types of information regarding surgical castration without anaesthesia (no information; negative; negative and positive; positive)		Survey 2:<br>58.8% aware of surgical castration without anaesthesia<br><br>63.3% aware that lack of access to pasture for livestock are common management practices in beef production systems	such practice;<br><br>74.8% were opposed to lack of access to pasture for livestock in confined systems; 17.7% were indifferent and 7.5% supported this management practice<br><br>Participants that were previously aware of surgical castration without anaesthesia, male participants, those that had visited a beef production farm and those that eat beef 3 or more times a week had higher odds of supporting // or being indifferent versus opposing surgical castration without anaesthesia;<br><br>Survey 2:

							Participants that were previously aware of surgical castration without anaesthesia had higher odds than those that were not aware of supporting or being indifferent versus opposing such practice (P 0.05).<br><br>No associations were found between participants that supported or were indifferent to surgical castration without anaesthesia and the other demographic data and socioeconomic groups (P > 0.1). Awareness and demographic/socio economic characteristics were not associated with participants supporting the lack of access to

							pasture for livestock in confined systems (P > 0.1)
(Loughnan et al., 2010) [42]	Questionnaire, 2009	No info. about the country where the study was made; N=108, 86 women	Eating meat through the withdraw of moral concern towards animals and deny their capacity to suffer	Participants were assigned to eat either beef jerky or cashews and rate taste dimensions. At the questionnaire participants were presented with twenty-seven animals and indicate those they feel morally to show concern for. Participants rate the cow's ability to experience a set of cognitive states and intellect	Moral concern for animals; Attribution of mental states for animals; Attribution for intellect in animals	Consuming meat leads to reduction in moral concern for animals: participants in the beef condition (M = 13.5) participants in the control condition (M = 17.3), t(97) = 2.53, p = 0.013. Moral concern: participants in the beef condition (M = 5.57) viewed the cow as less deserving than participants in the control condition (M = 6.08), t(97) = 2.20, p = 0.030. Attribution of mental states: participants in the beef condition (M = 5.54) did not attribute	n. a.

						significantly less capacity for sensation to the cow than participants in the control condition (M = 5.78), t(97) = 1.12, p = 0.271. Attribution of intellect: beef condition (M = 5.51) and control condition (M = 5.58), t(97) = 0.27, p = 0. 783. Eating meat did not directly lead to a reduction in the meat animal's perceived capacity to experience suffering	
(Malek et al., 2018) [43]	Online survey; 2015	Australian consumers, N=1009,<br><br>Cluster 1, 'neutral-FAW/neutral-environment	Beliefs regarding meat production; Farm animal welfare; Knowledge of livestock-management practices	No info.	Australian meat consumers on the basis of attitudes regarding farm animal welfare and the environmental impact of meat	People exaggerate the feelings and sensitivity of farm animals Pattern matrix 0.73 Structure matrix 0.69 C 0.50	Animal-welfare attitude: C1 (41%) –0.03a; C2 (29%) 0.04a; C3 (13%) 0.47b; C4 (7%) 0.94bc; C5 (7%) –1.40d; C6 (3%) –1.81d

		'; Cluster 2, neutral-FAW/concerned-environment'; Cluster 3, 'anti-FAW/neutral-environment'; Cluster 4, 'anti-FAW/apathetic environment'; Cluster 5, concerned-FAW/neutral-environment; Cluster 6, concerned-FAW/concerned-environment	(Likert-scale)		production	The needs of humans are more important than the needs of farm production animals Pattern matrix 0.71 Structure matrix 0.68 C 0.47 Humans have the right to use animals as they want 0.74 Structure matrix 0.76 C 0.58 I don't care about farm animal welfare issues Pattern matrix 0.61 Structure matrix 0.67 C 0.47;<br><br>70% of consumers remain unconcerned about FAW expressing neutral views	Clusters 5 and 6 comprised 7% and 3% respectively, of the study Sample had a concerned-FAW attitude, as indicated by negative regression scores on the human–animal hierarchy factor
(McGrath et al., 2013) [44]	Survey; 2011	Australian participants,	Public attitudes towards grief in	The questionnaire	To determine people's beliefs	90% respondents believed animals	Females were more likely than males to

		N=999, 47.8% women	animals	gave a definition of grief which was derived from the literature on human grief: An emotional reaction to loss, including sorrow, distress, sadness, anxiety and depression, which causes behavioural, emotional, mental, physical and social symptoms	as to whether animals could experience emotions, with a specific focus on grief;<br><br>Questions about pet ownership	could grieve according to our definition;<br>23% saying all animals could grieve;<br>67% saying some animals could grieve;<br><br>98% believed dogs, (N=883), chimpanzees (Pan troglodytes) (N=872; 97%), dolphins (N=850; 94%) and elephants (N=848; 94%) could grieve (Figure 1). The majority of respondents believed that cats (N=797; 88%), pigs (N=662; 73%), cows (N=637; 71%) and magpies (N=530; 59%) could experience grief. Less than (N=359; 40%), chickens (N=358;	respond that all animals could grieve (OR = 1.49, P = 0.007).<br>They were also more likely than males to say that cats (OR = 2.99, P < 0.001), dogs (OR = 3.82, P = 0.04), pigs (OR = 1.47, P = 0.02), chickens (OR = 1.69, P < 0.001), crocodiles (OR = 1.48, P = 0.004), bats (OR = 1.31, P = 0.049) (...) could grieve.<br>However, there was no statistically significant difference between males and females in response to whether dolphins, elephants, cows or prawns could grieve (P > 0.05).<br>As age increased, respondents were more likely to be

						40%), and crocodiles (N=353; 39%) could grieve	uncertain as to whether elephants could grieve (OR = 0.74, P = 0.02) and less likely to believe that cats (OR = 0.78, P = 0.01), cows (OR = 0.87, P = 0.03), pigs (OR = 0.85, P = 0.02), chickens (OR = 0.85, P = 0.004), crocodiles (OR = 0.85, P = 0.005), bats (OR = 0.86, P = 0.007) or turtles (OR = 0.82, P = 0.001) could grieve
(Monteiro et al., 2017) [45]	Questionnaire; 2012-2015	U.S students, N=302 (study 1)	To measure two positively related components of carnistic beliefs: carnistic defense and carnistic domination	No info.	Study 1: it measures both carnistic defense and carnistic domination beliefs	(“Humans should continue to eat meat because they've been doing it for thousands of years” A - 0.73; B - 0.70; C - 0.83; D - 0.85) (I've been eating meat my whole life, I could never give up” A - 0.68:	n.a.

						B - 0.73; C - 0.70; D - 0.73); (“Eating meat is better for my health” A - 0.73; B - 0.44; C - 0.84; D - 0.82); (“The production of meat causes animals to suffer” A - 0.52; B - 0.60; C - 0.43; D - 0.30). (“Animals aren't intelligent enough to suffer in intensive confinement” A - 0.61; B - 0.59; C - 0.58; D - 0.80); (“Animals are dirty and deserve to be eaten” A - 0.70; B - 0.65; C - 0.80; D - 0.91); (“Eating animals builds character” A - 0.60; B - 0.73; C - 0.75; D - 0.84); (“I have the right to kill any animal if I want” A - 0.67; B - 0.68; C - 0.66;	

						D - 0.78)	
(Peden et al., 2020) [46]	Questionnaire; 2017 - 2018	Citizens from Scotland, England and Ireland; N=58, 70,7% women<br><br>(Farmers, Veterinarians, and students excluded)	Belief in pigs (and cows) capacity to suffer (hunger, pain, fear, and boredom)	Measured participants' beliefs in the capacity of each of four species (dogs, cats, pigs, cows) to feel the sensations of hunger and pain and the emotions of fear and boredom (100 mm visual analogue scale)	If pigs and cows can experience hunger, pain, fear, and boredom	(Agreeability)<br>Hunger:<br>Dogs 88.0 %<br>Cats 85.6 %<br>Pigs 82.9 %<br>Cows 76.9 %<br>Pain:<br>Dogs 91.4%<br>Cats 87.6<br>Pigs 81.0%<br>Cows 85.5%<br>Fear:<br>Dogs 87.1 %<br>Cats 79.5 %<br>Pigs 76.6 %<br>Cows 75.6 %<br>Boredom:<br>Dogs 77.1 %<br>Cats 64.5 %<br>Pigs 57%<br>Cows 54.3 %	Women with higher scores than men for pain (M = 3.0, SE = 1.3, 95% CI = 0.4 to 5.6, p < 0.05) and boredom (M = 5.5; SE = 2.4, 95% CI = 0.9 to 10.1, p < 0.05), . Participants from Ireland scored higher about the capacity for pain than those located in England (M = 4.4, SE = 1.6, 95% CI = 0.6 to 8.3, p < 0.05) and Scotland (M = 5.0, SE = 1.7, 95% CI = 0.8 to 9.2, p < 0.05).
(Phillips et al., 2012) [47]	(Online) questionnaires, convenience sample, N.D.	Europe (Czech Republic 57% women, Great Britain 67%	Students' attitudes towards the welfare and rights of animals	No info.	Rating animal sentience (by species)	The overall order of attributed sentience (Mean rank [± SEM]) for the different species, from the highest	Animal sentience rating (by species): (Human Infant, Chimpanzee, Dog, Dolphin, Cat, Horse, Cattle, Pig: Rat, Chicken,

		women, Ireland 60% women, Macedonia 51% women, Norway 79% women, Spain 57% women, Sweden 82% women) and Asia (...)				to lowest, was human infant (10.7 [± 0.043]) > chimpanzee (9.7 [± 0.040]) > dog (9.5 [± 0.030]) > dolphin (8.6 [± 0.044]) > cat (7.7 [± 0.035]) > horse (7.2 [± 0.034]) > cattle (5.5 [± 0.034]) + > pig (5.2 [± 0.039]) > rat (4.8 [± 0.045]) > chicken (3.8 [± 0.032]) > octopus (2.7 [± 0.038]) > fish (2.6 [± 0.039])	Octopus, Fish) Czech Republic - 10.4b, 9.7bc 9.5ab, 9.0, 8.7b, 7.8bc, 4.7, 5.4c, 5.1b, 3.3d, 2.5c, 1.8cd; Ireland - 10.2bc, 9.5bc, 9.1b, 8.2, 8.7bc, 7.7c, 4.3, 6.3a, 4.5d, 4.1bc, 3.4ab, 1.9c; Macedonia - 10.3bc, 8.8d, 9.2b, 9.4, 8.5c, 6.5d, 4.3, 4.4e, 4.4d, 5.7a, 2.9bc, 3.6a; Norway - 9.9c, 9.9b, 9.2b, 8.0, 9.2ab, 7.7bc, 4.9, 6.6a, 5.1b, 3.6cd, 2.2cd, 1.7cd; Serbia -9.9c, 9.2c, 9.7a, 9.1, 9.0b, 7.9b, 4.4, 5.3c, 4.3d, 4.3b, 2.5c, 2.5b; Spain - 10.4b, 9.6b, 9.5ab, 8.9, 9.2ab, 7.3c, 5.0, 5.8b, 3.8e, 3.9c, 3.1b, 1.7cd, Sweden - 9.2c, 10.41a, 9.3ab, 9.4, 8.6c, 7.5c, 5.2,

							6.6a, 5.3b, 3.2d, 1.6d, 1.7cd
(Piazza & Loughnan, 2016) [48]	Experiment, questionnaire, 2016	U.K. participants, N=143, 73 N=117, 67 women	Study 2: manipulation of the intelligence of animals and its influence on moral standing. Study 3: learning about pig intelligence can lead to high levels of moral concern	Study 2: comparing a animal used for food (pigs) with a real animal not used for food (tapir) and with a fictitious animal hypothetically used for food in another culture. Study 3: Intelligence manipulation of pigs and dogs; and how pigs are treated	Study 2: manipulation of animal's intelligence (high vs. low) Categorization of animal as food or not food. Study 3: intelligence information utilised differently for the same animal target (pigs) when a person takes the perspective of another person versus themselves	Study 2: No difference in the perceived intelligence of the animal target, F(2, 65) ¼ .23, p ¼ .794, Z2 p ¼ .007 (Mpigs ¼ 5.55, SD ¼ 1.01; Mtapirs ¼ 5.62, SD ¼ 0.86; Mtrablans ¼ 5.44, SD ¼ 0.82). In the low intelligence condition pigs rated more intelligent (M ¼ 2.83, SD ¼ 1.27) than trablans (M ¼ 1.96, SD ¼ 1.26), p ¼ .028, but pigs and tapirs (M ¼ 2.21, SD ¼ 0.98), p ¼ .166, and tapirs and trablans, p ¼ .740, rated equally intelligent. Study 3:	n.a.

						Main effect of intelligence, F(1, 113) ¼ 20.90, p < .001, Z2 p ¼ .156, but no main effect of perspective, F(1, 113) ¼ 2.06, p ¼ .15, Z2 p ¼ .018.6	
(Piazza et al., 2018) [49]	Questionnaire; 2016-2018	U.S. omnivores, N=168, 68 women	Associating (through images) baby animals to meat may reduce appetite for meat	Study 1: images of baby animals, versus adult animals, as the source of meat; Study 2: replicated study 1 using a larger sample and two new animal sources Study 3: only a baby animal presented (no meat)	Testing the demotivating influence of baby animals on appetite for meat	Meat sourced from a baby animal was rated overall less appetising (M = 49.28, SD = 32.91) than the same meat sourced from an adult animal (M = 59.42, SD = 31.83).	Collapsing across gender, the meat was least appetising when it was presented along with an image of a baby animal (M = 59.38, SD = 35.14) as the source, and most appetising when it was presented without any image of the animal source (M= 76.89, SD = 25.99), with the adult animal source falling in between (M = 71.56, SD = 27.17)
(Queiroz, et al., 2018) [50]	Online survey; 2016-2017	Brazilian citizens	Perceptions of Brazilian citizens	No info.	Poultry supply chain;	Conditions of animal welfare	Socio-demographic characteristics

		N=1614 Total (N=728=poultry chain; N=586=beef supply; N=300 dairy supply chain)	about general conditions of animal welfare in the poultry, beef, and dairy supply chains		Beef supply chain;<br>Dairy supply chain;<br>(Conditions of animal welfare<br>Awareness animal welfare<br>Transportation<br>Slaughtering)	(%) (1:very bad; 2:bad; 3:regular; 4:good; 5:very good):<br>Poultry supply chain 1:29.7; 2:29.9; 3:30.63; 4:9.06; 5: 1.23<br>Beef supply chain 1:14.0; 2:27.1; 3:42.83; 4:14.85; 5:1,19<br>Dairy supply chain 1:14.0; 2:27.0; 3:47.3; 4:10.3; 5:1.3<br>Awareness animal welfare (0:no; 1:yes)<br>Poultry supply chain 0:15.7; 1: 84.3<br>Beef supply chain 0:18.3; 1:81.7<br>Dairy supply chain 0:19.7; 1:80.3<br>Transportation (%) (1:strongly disagree; 2:disagree; 3:neutral; 4:agree; 5: strongly agree)	(age, gender, pet ownership, and consumption of animal products) did not significantly have an impact on participants' perceptions about the general conditions of animal welfare in any supply chain. Participants who reported the previous contact with poultry farms were more likely to perceive the general conditions of animal welfare in the poultry supply chain as being bad compared to participants who had not reported the previous contact. Participants who reported a higher level of knowledge about poultry and dairy supply chains were

						Poultry supply chain 1:40.4; 2:37.4; 3:8.8; 4:7.7; 5:5.8<br>Beef supply chain 1:27.6; 2:42.0; 3:13.9; 4:13.5; 5:2.91<br>Dairy supply chain (not measured)<br>Slaughtering (%) (1:strongly disagree; 2:disagree; 3:neutral; 4:agree; 5:strongly agree)<br>Poultry supply chain 1:32.7; 2:36.3; 3:12.1; 4:11.4; 5:7.6<br>Beef supply chain 1:25.7; 2:41.1; 3:10.2; 4:16.3; 5:5.7<br>Dairy supply chain (not measured)	more likely to perceive the general conditions of animal welfare in the poultry and dairy supply chains as being bad compared to those participants who reported a lower level of knowledge about these supply chains

(Rice et al., 2020) [51]	Telephone survey (CATI); 2018	Australian consumers, Pre campaign N=278, 60% women<br>Post campaign N=224, 48%	Impact of a negative media event on public attitudes towards animal welfare	Australian media campaign exposing animal cruelty in live export of sheep by sea	(Questionnaire item)<br>Animal rights:<br>Sheep and beef cattle have the same right to life as domestic animals;<br>Sheep and beef cattle have the same feelings as domestic animals;<br><br>Approval of husbandry practices:<br>Mulesing<br>Crutching<br>Dehorning<br>Pre-slaughter stunning<br>Curfew<br>Tail docking<br>Ear tagging<br>Hot iron branding<br>Castration<br>Feedlotting<br>Spaying<br><br>General welfare:<br>Social contact with animals of	(N=278 pre-event) (Mean)<br>Caring for and balancing the needs of pets and people 4.11<br><br>Approval of husbandry Practices 3.00<br><br>Land beef transport conditions 2.53<br><br>Sea beef transport conditions 2.18<br><br>Sea sheep transport conditions 2.12	(N=224 post-event) (Mean)<br>Caring for and balancing the needs of pets and people 4.11<br><br>Approval of husbandry Practices 3.09<br><br>Land beef transport conditions 2.46<br><br>Sea beef transport conditions 2.01<br><br>Sea sheep transport conditions 1.91<br>Despite the wide media coverage respondents' underlying attitudes and beliefs about farm animal welfare were not affected

					the same species<br>Contact with their young<br><br>Land beef transport conditions:<br>Space per animal<br>Provision of food and water<br>Ventilation<br>Journey length<br>Road/truck conditions (e.g. sound, vibration, braking levels<br>Loading of animals onto vehicles (e.g., use of handling aids, human handling);<br><br>Sea beef transport conditions:<br>(similar items)<br><br>Land sheep transport conditions:<br>(same items)		

					Sea sheep transport conditions (same items)		
(Robbins et al., 2019) [52]	Online survey; N.D.	U.S. consumers, N=430 (Experiment 1), 47% women	Public perceptions of tie stall housing for dairy cattle	Experiment 1: written information about "tie stall" barns, and implications for cows	Experiment 1: indicate how much they had read or thought about tie stall housing for dairy cattle (5-point scale)	Awareness on tie stall housing<br><br>Total None at all 53.6%<br><br>Experiment 1: A total of 65% participants indicated they would support a ban on tie stalls. Participants predicted 63% of their fellow survey respondents would support the ban	Awareness on tie stall housing<br><br>Experiment 1 None at all 51.2% Experiment 2 None at all 56.5%<br><br>Liberal political orientation increased the probability of supporting the ban, and female participants tended to be more supportive of the ban
(Rovers et al., 2018) [53]	Qualitative study with focus group; 2015	German citizens N= 1420, 49.4% women	Citizens' perceptions of recent livestock production in Germany for different animal species (pigs, cattle or poultry)	No info.	Actual animal husbandry with respect to housing systems, animal health and well-being regarding the Scientific Advisory Board's	In all discussions, the topic "factory farming" was discussed critically. The lack of free movement, too little space per animal and, for	(Clusters 1, 2, 3)<br>1. supporters of efficient animal husbandry (More often male, Knowledge of animal husbandry average or bellow; more South, Less:

					(Total=9)<br>guidelines:<br>4. sufficient space per animal (only this one was selected)	citizens, non-transparent, locked systems, especially in pig and poultry production, were mostly mentioned. The use of technology (e.g. for feeding processes or milking in case of cows) is seen as a negative;<br>Pig production concerns about space per animal, especially sows.<br>Cattle production Participants were aware of limitations and said that dairy cattle are often kept only in stables. Some respondents even stated that cows were fixed in grids;<br>In discussions about poultry production participants often	North, West (36%)<br>2. evaluating pros and cons More often female, Knowledge of animal husbandry above average<br>More: West, East<br>Less: South, North (36%);<br>3. Opponents of an efficiency driven husbandry (28%)<br>More often female, Knowledge of animal husbandry above average (even better than the 2nd cluster)<br>More: North, Less: East

						described keeping systems with bars and cages for laying hens and broiler chicken as very negative	
(Ryan et al., 2015) [54]	Online survey; 2014	Canadian and U.S. participants, N= 242, 77% women	If respondents believe if pregnant sows should be housed in gestation stalls or in groups	Provided information through scientific papers, You Tube videos, Google images and questions	Participants support of gestation stalls	After being provided with additional information about gestation stall, the initially supportive group (30.4%) dropped to 17.8%	Younger participants, Canadians, participants with higher education, and females were less supportive of gestation stalls
(Sato, et al., 2017) [55]	Online survey; 2015	U.S. residents; N=199	Views of an Ideal Pig Farm	"Take a short survey asking your opinion of pig farms. We want to know what characteristics you think make the 'ideal' pig farm". Due to the possibility of these different interpretations, we used both terms "pig farm" and	"What do you consider to be an ideal (pig, pork) farm and why are these characteristics important to you?".	Space, largely associated with animals0 freedom to move and the ability to perform innate natural behaviours, was the main theme addressed by the respondents. Many described their desire for housing systems that are not overcrowded and that do not limit restriction of	74% of the respondents addressed concerns relating to animal welfare, including space to move, feeding, contact with outdoors or nature, absence of pain, suffering, and mistreatment; 44% reflecting concerns about the animals' housing or space.

				“pork farm”,. Participants were provided to a single open-ended question: “What do you consider to be an ideal (pig, pork) farm and why are these characteristics important to you?”		movement;<br>lack of space and overcrowding represents a greater concern for our participants than a confined housing system;<br>absence of pain and suffering	20% of the respondents pointed the need for an outdoor area for pigs where they could move around, interact with each other and perform natural behaviours;<br>6% mentioned the term “free range”.<br>“Free” or “freedom” (16%);<br>“Absence of pain and suffering” (12%)
(Sødring et al., 2020) [56]	Online survey; web-panel, 2016	Norwegian consumers, N=1002	Consumer attitudes towards castration of pigs	Respondents were asked to answer the acceptability of castration with following info available: “Norwegian pigs are slaughtered at 4–6 months of age. (...)The current practice in Norway is that all male pigs	Castration with anaesthesia;<br>Castration without anaesthesia	60% of the web-panel participants answered that they were unaware of surgical castration in pigs;<br>Both surgical castration with anaesthesia was generally highly accepted among the participants;<br>88%;<br>5%	Twice as many women (24%) than men (11%) considered the current practice of surgical castration with anaesthesia to be unacceptable<br><br>Consumers aged 18–29 deemed surgical with anaesthesia “completely

				(except breeding animals) are given local anaesthesia and castrated by same low levels that are achieved with surgical castration.”		characterised surgical castration with anaesthesia as “not acceptable”. Surgical castration without anaesthesia was considered unacceptable among (70%); 22% considered surgical castration without anaesthesia to be acceptable (8% “completely acceptable”; 14% “acceptable with reservation”	acceptable”; 53% consumers over 60 considered surgical castration with anaesthesia to be “completely acceptable” With additional information unacceptability for castration without anaesthesia was 77%
(Sonntag & Sprilleer, 2019) [57]	Online survey; 2016-2017	German citizens (Pilot study) N=202, 51.5% women (Representative study) N=1009, 52.7% women	Measuring public moral concerns (hens laying eggs in battery cages)	No info.	I consider keeping laying hens in battery cages as disrespectful to hens<br><br>I consider it unjust to keep egg-laying hens in battery cages	Battery eggs (N=1009)<br><br>Values (VAL) 81.3%<br><br>Cognitive Assessment (COG) 65.1%<br><br>Readiness to	Pilot Study (N =202)<br><br>Values (VAL) AVE = 0.655 CR = 0.930<br><br>I consider it unjust to keep egg-laying hens in battery cages 0.768

					Battery cages constitute disrespectful handling of animals to me<br><br>Battery cages constitute disrespectful handling of animals to me<br><br>It is alright to keep egg-laying hens in battery cages	Act (ACT)<br>36.4%<br><br>No Willingness to Accept (WTA)<br>70.2%	I feel sorry for the egg-laying hens in battery cages 0.864<br>Battery cages constitute disrespectful handling of animals to me<br>0.813<br>Egg-laying hens must not be kept in battery cages 0.740<br><br>Emotions (EMO)<br>AVE = 0.760<br>CR = 0.940<br>Keeping egg-laying hens in battery cages infuriates me<br>0.920<br><br>The fact that there are still battery cages makes me mad<br>0.916<br><br>Cognitive assessment (COG)<br>AVE = 0.601<br>CR = 0.858<br>I do not want a prohibition of keeping egg-laying

							hens in battery cages - (no data)<br>In my point of view, battery cages are not a violation of animal welfare 0.750<br>Keeping hens in battery cages is legitimate 0.788<br><br>Readiness to act (ACT) AVE = 0.610 CR = 0.861<br>I like to inform people that laying hens are still kept in cages 0.828<br><br>I am generally willing to promote the abolition of battery cages 0.848
(Soriano et al., 2021) [58]	Survey; 2017	Brazilian citizens, N=209, 70% women (farmers sample excluded)	Lay citizen respondents' attitudes regarding animal welfare and animal maltreatment	No info.	Tail docking without anaesthesia<br><br>Movement restriction<br><br>Animal isolation	(Animal maltreatment items scales)<br>Docked tail without anaesthetic use 80.9%<br>Movement restriction 68.5%<br>Animal isolation	n. a.

						from other animals 93% Sheep farming 50%	
(Tamioso et al., 2018) [59]	Online survey, 2014-2016	Brazilian and French citizens (OB) Total=388, 70.6% women (biologists, veterinarians and animal scientists excluded)	Perception of animal welfare, sheep welfare, sentience and animals' emotional capacities.	At question 8 (Q.08) management procedures from the question (identification, castration, tail docking, shearing, reproductive techniques and weaning) are described, with definitions on how they are commonly performed.<br><br>(Rate according to your perception of suffering) Identification: through ear notching or punching, tattooing, ear	Animal welfare<br><br>Welfare for farm animals<br><br>(Likert-scale) Sheep that are raised indoors, under intensive management systems, have low levels of welfare. III. Sheep are capable of feeling emotions, such as fear and happiness, in addition to suffering.<br><br>Sheep clearly express how they feel, that is why it is easy to identify if they are in positive or negative	Freedom from hunger, thirst and malnutrition 25.7%, Freedom from pain, injury and disease 14%, Freedom to express normal behaviour 11%, Freedom from discomfort 18%, Freedom from fear and distress 27% and Other 8%<br><br>46.9% believed that welfare is not taken into consideration for farm animals<br><br>Aspects related to freedom from discomfort were cited 31.3% as the most important issues of animal farming	Aged 40±49 years-old / with secondary or less educational levels tended to agree that sheep that are healthy and grow well have their welfare guaranteed; Older and with lower education seem to view animal welfare mainly in terms of physical health;<br><br>Less participants in France agreed that sheep are capable of feeling emotions;<br><br>Citizens from Brazil showed higher perception of sheep suffering during castration, tail docking, shearing, reproductive techniques and weaning;

				tagging or microchipping.<br>Castration: removal or destruction of the testicles, through rubber rings, emasculator/burdizzo or surgery.<br>Tail docking/ tail removal: through rubber rings, cauterization using a hot docking iron or surgery.<br>Shearing: cutting or shaving the fleece/wool, though the use of electric shears, shearing machines or scissors.<br>Reproductive techniques: artificial insemination, synchronizatio	situations<br><br>(Likert-scale) classify the management procedures that are frequently performed on sheep farms: identification, castration, tail docking, shearing, reproductive techniques and weaning;<br><br>(Likert-scale) classify the ability of each animal to feel emotions: pigeon, butterfly, human baby, rat, dog, chicken, fish, sheep, cattle, cockroach and wolf	that contribute to good animal welfare<br><br>Sheep that are raised indoors, under intensive management systems, have low levels of welfare, 61.3% strongly agreed<br><br>(Levels of agreement concerning sheep welfare and sentience)<br><br>Sheep that are raised indoors, under intensive management systems, have low levels of welfare 61.3%;<br>Sheep are capable of feeling emotions, such as fear and happiness, in addition to suffering 75%;<br>Sheep clearly	Female attributed higher scores of suffering to sheep during tail docking, reproductive techniques, weaning than male.<br>Higher concern from women toward management procedures was expected, as women tend to react more emotionally and empathetically to animal suffering;<br><br>A general high perception of sheep pain was noted among older<br><br>French citizens with higher educational levels attributed severe suffering to sheep during identification1, when compared with other groups;<br><br>Women attributed

				n of estrus (through the use of intravaginal sponge impregnated with progestagen) and laparoscopic embryo transfer. Weaning: separation of ewes and lambs before the lambs reach 6 months of age<br><br>Q10-12 Watch the video and describe in 3 adjectives, at most, how the animal is feeling<br><br>Q13-15 Watch the video again and choose, at most, 3 adjectives that best describe		express how they feel, that is why it is easy to identify if they are in positive or negative situations 75%<br><br>(Levels of agreement about the statement) ªSheep are capable of feeling emotions, such as fear and happiness, in addition to sufferingº<br><br>Mammals were given the highest scores of sentience by the respondents, followed by birds, fish and invertebrates;<br><br>Higher scores attributed to dogs and human baby. The wolf was perceived as a highly sentient	higher scores of sentience to animals than men<br><br>Older respondents showed higher levels of perception of animal welfare issues (e.g., knowledge about animal welfare, perception and identification of sheep emotions and sheep suffering);<br><br>For the videos showing positive events 68.0% attributed adjectives of positive valence to sheep emotions Concerning the video showing a negative event 89.4% believed that sheep experienced negative emotions

				how the animal is feeling		being by the surveyed participants	
(Tawse, 2010) [60]	Questionnaire	U.K. consumers, N=173	(Part B of the questionnaire) To measure participants' attitudes towards pig welfare on farms	No info.	Statements along with five-point scale one which respondents can indicate the extend they agree or disagree:<br><br>Intensive pig farming is cruel;<br><br>It does not matter how pigs are reared as they don't know any better<br><br>Pet animals deserve better treatment than farm animals	Consumers who show the highest levels of concern are more likely to associate positive attributes with pigs, more likely to be interested in animals, more likely to be aware of modern pork production methods, and are more likely to have been exposed to a working pig farm sometime in the past;<br>Awareness of pork production methods<br>Students who showed a greater awareness of pork pro- duction methods had significantly	n.a.

						higher PLIs, showed a greater concern for farm animal welfare, showed a greater concern for pig welfare on farms Statements: “I think intensive (standard, non-free range) pig farming is cruel” rs 0.378, p 0.000, P >0.01; “It does not matter how pigs are reared as they don't know any better” rs -0.459, p 0.000 P P >0.01 “Pet animals deserve better treatment than farm animals.” rs -0.257, p 0.001 P >0.01	
(Tian et al., 2016) [61]	Questionnaire, (online in France); N.D.	French and Chinese participants, N=520, 176 women	Focusing on meat production and meat consumption was examined whether	Study 1: Abattoir condition: photo of a cow with a short	The meat paradox in the meat production stage: abattoir condition; pasture	Cognitive dissonance in response to the meat paradox is observed among French	Study 1: Manipulation check - 86% (98% French) participants in the abattoir condition

		*Only French data included in this systematic review (see inclusion criteria)	participants used reduction of mind attribution to food animals as strategies to reduce cognitive dissonance from the meat paradox	statement saying that the cow will be sent to the abattoir tomorrow. Pasture condition: photo of a cow with a statement that the cow will be sent to another pasture tomorrow. Meat condition: diagram of a cow that displays the names of the different kinds of beef from the various parts of the cow's body; Control condition had no experimental manipulation Study 2: recipe in a short text, the	condition; meat condition; control condition	participants. Study 1: focused on the meat production stage, found that the abattoir condition led most participants to think about the slaughter. The results that participants reduced their willingness to mind attribution to cows when the animal origin of meat was explicitly shown in the pasture; Meat conditions supported the hypothesis that people would reduce mind attribution to animals to resolve cognitive dissonance resulting from the meat paradox. Study 2: focused on the meat	mentioned the killing, 47% did so in the pasture condition (38%);. Participants who mentioned the slaughter did not attribute less mind to cows (Mwillingness ¼ 3.58, SDwillingness ¼ 1.35; Magency ¼ 27.63, SDagency ¼ 7.19; Mexperience ¼ 36.27, SDexperience ¼ 5.87) than those who did not mention the slaughter (Mwillingness ¼ 3.67, SDwillingness ¼ 1.39; Magency ¼ 27.78, SDagency ¼ 6.61; Mexperience ¼ 36.61, SDexperience ¼ 5.53), ps > .10.<br><br>Mind perception of

				recipe with animal image condition showed a photo of a cow, illustrating the source of beef in the dish, and the recipe with dish image condition showed a photo of the dish; the recipe alone condition described the recipe only in a short text; the control condition did not present the recipe but moved directly on to the dependent measures after showing a sentence of acknowledgment for participation		consumption stage, revealed that the recipe with animal image condition led participants to like a dish less, compared with the recipe with dish image and recipe alone conditions. The marginally significant findings that participants attributed less mind to cows strategies to deal with the dissonance resulting from the meat paradox	cows - French participants did show significant differences between the dissonance manipulation conditions, F(1, 498) ¼ 3.91, p ¼ .009. French participants in the pasture condition (p ¼ .069) and meat condition (p ¼ .088) attributed less agency to cows than those in the control condition: French participants showed a marginally significant tendency to attribute less mind to cows on the dimension of agency after the animal origin of meat had been made explicit in the pasture and meat conditions,

							compared to the control condition.<br><br>Study2:<br>Participants in the recipe with animal image condition (M ¼ 4.63, SD ¼ 1.97) reported less liking than those in the recipe with dish image (M ¼ 5.39, SD ¼ 1.71) (p ¼ .001) and recipe alone conditions (M ¼ 5.60, SD ¼ 1.34), p < .001, leading us to think that greater cognitive dissonance had been induced in the recipe with animal image<br>Condition;<br>French participants reported hunger (M ¼ 2.80, SD ¼ 1.83), F(1,510) ¼ 7.07, p ¼ .008, h2P ¼ .014.<br><br>Participants<br>in the recipe with

							animal image condition (M ¼ 26.18, SD ¼ 7.07) and in the recipe with dish image condition (M ¼ 25.97, SD ¼ 7.15) attributed less mind to cows than those in the recipe alone condition (M ¼ 28.13, SD ¼ 7.23), p's ¼ .10 and .054
(Tiplady et al., 2013) [62]	Survey, 2011	Australian participants, N=157, 90 women	To ascertain the responses of the public who had encountered the media coverage of cruelty to Australian cattle	Two-week period of the ‘‘Four Corners’’ footage first being broadcast - an investigation into the treatment of Australian cattle exported to Indonesia that included several cruelty practices	Several optional emotional reactions were presented (to look away; to cry; feeling sad; etc.);<br>Several optional actions were presented (told others; sought counselling; wrote a letter to a politician; etc.)	105 respondents indicated they had encountered several sources of media coverage about animals.<br>Common reactions:<br>Pity for the cattle 85%<br>Sadness 72 %<br>Anger 68%;<br>Pleased that the footage had been broadcast 75 %;<br>Determined to take action to stop live export	Women were more likely than men to feel sad and angry;<br>Women were also much more likely than men to look away or stop listening to the media coverage;<br>Women were more likely than men to perform any action and to discuss the media coverage with others;<br>Those with a university bachelor or postgraduate level of

						26%;<br>The coverage was manipulating the public 24%;<br>From other responses (27%) 33% felt disgust and 12% felt sick	education to be more likely to sign a petition to ban live export

(Tomasevic et al., 2020a) [63]	Online survey; Exploratory, 2017	13 Eastern European countries (Bosnia and Herzegovina N=309, 55.3% women, Bulgaria, N=352, 59.1 women, Czech Republic N=506, 61.1% women, Croatia N=301, 60.5% women, North Macedonia N=284, 51.8 women, Hungary N=400, 44%, Moldova 504, 68.9% women, Poland N=504, 51.4% women,	To investigate the Eastern European consumers' beliefs and attitudes toward animal welfare	No info.	Statements comprised beliefs dealing with particular aspects of animal management (transport, rearing space, way of rearing, and slaughter), possible consequences and ethical issues (dignity, mistreatment and welfare) (Seven-point Likert scale)	Eastern European consumers are not clear about whether the animals that are consumed are transported incorrectly (4.4); If slaughter systems should be improved to avoid animal suffering, the average score for all Eastern European consumers was above the "Agree" mark (5.4.)	Women were a bit (4.5) closer than men to agree with the statement that transportation of animals is inadequate (4.2). Older people (>55 years old) were more affirmative about that belief than the young ones (<36 years old) Bulgarian consumers agree more with the statement that transportation of animals is inadequate (4.8), and the closest to disagree were Hungarian consumers (3.9); Women (5.5) agreed more strongly than men (5.2) slaughter systems should be improved, and consumers that grew up in urban areas (5.5) more strongly than

		Romania N=556, 50.2% women, Serbia N=661, 52.3 women, Slovakia N=296, 60.8 women, Slovenia N=215, 54% women, and Ukraine N=701, 60.1% women) (Total N=5508)					consumers that were raised in rural areas (5.2); Consumers from Slovenia agreed very strongly and gave significantly highest score slaughter systems should be improved (6.7), followed by consumers from Bulgaria (5.7), North Macedonia (5.7), Romania (5.7), and Ukraine (5.6); Bulgarian (73%) and Czech (64%) consumers and by half of the Romanian ones (50%). It is the cluster with the highest proportion of urban consumers (42%), consumers between 36 and 55 years old (37%), and wealthy consumers (41%). This group of consumers is also concerned about

							animal welfare since they consider, like cluster 2, that ‘slaughter systems should be improved to avoid animal suffering’ (5.6)

(Tomasevic et al., 2020b) [64]	Online survey; Exploratory, 2017	3 Eastern European countries (Bosnia and Herzegovina N=324, 48.4% women, Bulgaria, N=352, 59.1 women, Czech Republic N=510, 61.4% women, Croatia N=301, 60.5% women, North Macedonia N=285, 51.9% women, Hungary N=400, 44.0%, Moldova 300, 68.7% women, Poland N=504, 51.4%	The present work has an exploratory character and aims to investigate the beliefs and attitudes of Eastern European consumers regarding boar taint, surgical castration immunocastration and perception of meat from castrated pigs		Questionnaire included 8 statements (beliefs) about castration and perception of meat from castrated pigs. Respondents had to indicate their degree of agreement (Likert scale)	When Eastern European consumers were asked if they believed that surgical castration produces pain to the animals, they agreed with the statement (average score 4.9). Average Eastern European consumers' opinion about whether surgical castration is harmful or beneficial tends to move to the beneficial side (4.6).	When Eastern European consumers were asked if they believed that surgical castration produces pain to the animals, the difference between men (4.9) and women (5.0) and consumers that grew up in urban (5.0) and rural areas (4.8), although significant, is quite irrelevant. Hungarian consumers, had significantly the lowest score (4.2) and were the only ones that answered on average 'neither agree nor disagree'. Bulgarian consumers had significantly the highest average score (5.6) and were the only ones that answered on average 'agree

		women, Romania N=557, 50.3% women, Serbia N=678, 52.2% women, Slovakia N=301, 60.1 women, Slovenia N=246, 55.0% women, and Ukraine N=750, 59.7% women) (Total N=5508)					strongly' that surgical castration produces pain to the animals; The attitude about surgical castration being beneficial was significantly more pronounced for the consumers that grew up in rural (4.7) than in urban (4.5) areas. The same could be observed for the respondents older than 55 years compared to those younger than 36 years and in men (4.6) compared to women (4.5) Large differences between countries were observed, with Hungarian respondents (6.2) most strongly thinking that surgical castration is beneficial and Bulgarian ones (2.6) with a very

							strong opinion that it is harmful. On a beneficial end of the scale, consumers from Ukraine (5.3), North Macedonia (5.1) and Croatia (5.0) could be placed, and on the harmful end of the scale consumers from Poland (3.5)
(Tonsor et al., 2009) [65]	Survey; 2008	U.S. Consumers, N=1001, 50% women	Consumer perceptions regarding swine gestation crates	Respondents were informed about a ballot initiative in California for bane gestation crates.	If your state income taxes increased by $T per year due to the ban, would you vote FOR or AGAINST the ban? Please answer as if you were actually voting on a real referendum involving real taxes	Animals should not reside in confined areas like gestation crates - Mean 4.987<br>I rarely think about the use of gestation crates when purchasing pork 5.275	Consumers to generally (69%) support a gestation crate ban when asked in a typical ballot setting, without direct reference to tax implications; However, when residents are told that state income taxes would increase if passed; resident support fell to 31%.<br>Age is the only demographic

							variable identified to significantly impact WTP, with older consumers having less WTP higher taxes to support a ban
(Vandresen & Hötzel, 2021) [66]	Online survey; 2020	Brazilian citizens, Farrowing crates (FC) N=395 53% women, Loose Farrowing pens (LP) N=384 48% women, Outdoor farrowing (OF) N=392 55% women (Total N=1171, 52% women)	Citizens' attitudes regarding farrowing housing systems for sows and their piglets	Participants read a short text about farrowing housing (crates, loose pens, and outdoors) and were provided six images displayed on graphics to illustrate each housing system	(Likert-scale) "Do you consider this farrowing system appropriate?"<br><br>Do you approve of this farrowing system?"<br><br>"Do you consider this farrowing system acceptable?"<br><br>Rate the quality of life of the sow and piglets in the farrowing system<br><br>+ Open question asking	Previous awareness of the housing system Yes N=601 (51.32%)<br><br>(5-Likert-scale, with higher numbers indicating more positive attitudes)<br><br>Attitude (Mean)<br><br>Crates (N=395) 1.64 SE 0.06<br><br>Loose pens (N=384) 3.02 SE 0.06<br><br>Outdoors (N=392) 4.36 SE 0.07	Attitude scores were most negative toward the farrowing crates and most positive toward the outdoor farrowing system<br><br>Housing system ($F_{2, 1160}$ =640.5, $p < 0.001$), participant's sex ($F_{1, 1160}$ = 38.2, $p < 0.001$), and days per week they ate meat ($F_{4, 1160}$ = 4.35 $p < 0.01$) influenced participants' attitude scores.<br><br>There was also an interaction between sex and farrowing system ($F_{1, 1160}$ = 11.9, $p < 0.001$),

					participants to justify their answer to these questions	Participants described farrowing crates as ethically wrong due to the negative impact on animals' quality of life and freedom of movement. Many used the words "cruel", "inhumane", and "suffering" to describe farrowing crates; Restriction of space, especially regarding the sow, was a main concern about farrowing crates	with women showing lower attitude scores than men on the farrowing crate and loose farrowing pen systems (Tukey's HSD test, $p < 0.0001$)
(Vanhonacker & Verbeke, 2011) [67]	Survey and online interviews, 2008-2009	Consumers from Belgium, France, Germany, the Netherlands N=1031, 494 women	Consumer awareness and response to the possible use of a vaccine method to control boar taint v. physical piglet castration with	Textual provided information about Boar taint; Physical castration with anaesthesia; Vaccination against boar	Boar taint; Physical castration with anaesthesia; Vaccination against boar taint	Polled sample (Awareness)<br>Boar taint<br>Never heard of it 53.7%<br>Heard of, but do not know much about 35.0%<br>Heard of and	(Acceptance)<br>Physical castration Mean (S.D:) 3.74 (1.74)<br>Vaccine method (S.D.) 5.38 (1.61)<br><br>Differences in scores between

		N=993, 477 women<br>N=1006, 468 women<br>N=1001, 479 women<br><br>(Total=4031)	anaesthesia:	taint		know a lot about 11.3%<br>Physical castration<br>Never heard of it 48.5%<br>Heard of, but do not know much about 33.1%<br>Heard of and know a lot about 18.4%<br>Vaccine method<br>Never heard of it 86.8%<br>Heard of, but do not know much about 11.2%<br>Heard of and know a lot about 2.0%	both methods were most pronounced in Belgium, France and the Netherlands. German responses were characterized by a higher number of neutral answers on both questions. For both methods, a similar degree of confidence was expressed with regard to eliminate boar taint. Absolute confidence scores were slightly in favour of the physical castration method in Germany, whereas the opposite was found in all other countries
(Vanhonacker et al., 2009) [68]	Web-based questionnaires; 2008	Belgium, N=225, 45% women	Consumer awareness of piglet castration and attitudes	Respondents were provided with printed information three	Respondents were provided with a list of 27 items related to characteristics	Awareness of piglet castration: 50% of respondents reported they	Message condition did not have a significant impact on the respondents’

			towards immunocastration	components. 1. General description as to why castration was performed, the manner in which it is currently practised (surgical castration without anaesthesia), and a short explanation about immunocastration. 2. Description of the advantages of immunocastration: reduction of pain and stress when compared to surgical castration, a reduction of aggression and sexual behaviour compared	of and practices in the pig production chain, among them piglet castration	were aware that male piglets are castrated. This group is further termed as the 'aware group'. From the aware group, 78% was able to report that the reason for this practice was related to meat quality, with the vast majority referring to the odour of the meat and 12% explicitly mentioning boar taint. 40% is well informed on the topic; Concerns about pig production practices: Space availability 5.12 (± 1.48) Slaughter without pain and stress 5.27 (± 1.56) Transport climate 4.96 (± 1.65) Stress 5.06 (±	general attitude towards immunocastration; respondents evaluated immunocastration slightly better than surgical castration with 60% of the sample indicating a preference for immunocastration over surgical castration

				to entire males, a better feed conversion ratio compared to barrows and sows, and the reassurance that such meat is safe for the consumer. In the third component, the major downsides/risks associated with immunocastration were given, in terms of the danger of self-injection, the costs associated with injecting pigs (...)		1.54)<br>Tail docking 4.17 (± 1.77)<br>Castration 4.14 (± 1.73)	
(Vanhonacker et al., 2009) [69]	Survey;<br><br>(dataset 1 2000, 2001, 2002)<br>(dataset 2 2006	Belgium citizens, N=179 59.2% women N=185 60.5%	Citizens' concerns related to stocking density, pen size and group size in farm animal	No info.	(Dataset1 and Dataset 2) (Likert-scale) Evaluative beliefs concerning the aspect of	(Dataset 1) pen size and stocking density were attributed the second and third highest perceived	(Dataset 1) no significant effect of gender on concern towards stocking density was found, despite a more negative

		women N=157 66.9% women N=521 62.2% women N=459 51.5% women	production		stocking density (number of animals housed per m2, i.e. the concept of space allowance) as well as of its two components – pen size and group size<br><br>(Dataset 2) Group size	importance score among other 23 aspects;<br><br>Pen size and stocking density were ranked 11th and 12th respectively, in terms of evaluative belief;<br><br>When ranking all 23 aspects from highest to lowest concern (i.e. perceived importance score minus evaluative belief score), stocking density and pen size ranked third and fourth. The highest concern was expressed for the transport of animals and the (un)loading of animals;<br><br>(Dataset 1) stocking density's perceived	evaluative belief among males. (Dataset 2) highly significant effect of gender was found, with a higher concern among women. Women reported both a higher perceived importance and a more negative evaluative belief;<br><br>Concerning age, a significant effect on concern was found in both datasets. In dataset 1, the concern decreased from the youngest to the oldest age category. In dataset 2, concern scores differed between the youngest two categories and the oldest two categories;

						importance was very high (ranking 7th on 72 aspects);<br>Pen size ranked relatively high (14th);<br>Group size, ranked only 58th;<br>Stocking density was attributed the lowest mean evaluative belief score (i.e. perceived as the most problematic aspect in relation to farm animal welfare) of all 72 aspects, while pen size also scored as highly "problematic" (6th).	
(Vanhonacker, et al., 2016) [70]	Questionnaire; 2011	Citizens from Belgium; N=541, 46.3% women	Citizens' and broiler producers' perceptions of broiler chicken welfare in Belgium versus Brazil	No info.	Statements were included to document general attitudes towards animal welfare: 'Broiler chickens suffer during their life on the	Mean scores (± SD) 'Broiler chickens suffer during their life on the farm' 4.31 (1.63) |t| 23.65; 'Broiler chickens suffer during transport' 5.21	Citizens perceived that broiler chickens suffer, while producers strongly disagreed;<br>Both groups believed that suffering is the

					farm'; 'Broiler chickens suffer during transport'; 'Broiler chickens suffer during Slaughter' (Likert scales)	(1.36) |t| 19.63; 'Broiler chickens suffer during slaughter' 4.73 (1.59) |t| 14.71	lowest 'on the farm' as compared to 'during transport' or 'during slaughter'
(Ventura et al., 2013) [71]	Online forum; 2010-2011	Diverse range of participants (Canada, U.S., others) N=163, 74% women (Groups 1-4 38-43 participants per groups)<br><br>Participants from the dairy industry (N=28) excluded	To examine the views of participants on the specific practice of separating the dairy calf from the dam at or soon after birth	Was provided background information on cow-calf separation pros and cons for animals and producers	"Should dairy calves be separated from the cow within the first few hours after birth?" and could respond "Yes, because…," "No, because…," or "Neutral, because…".	Overall, 44% supported early separation (chose "yes"), 48% were opposed (chose "no"), and 9% were "neutral"	Support for early separation was higher among males, people in their 20s, people with graduate education, and participants originating from the United States.
(Ventura et al., 2016) [72]	Exploratory survey, 2014	Canadian participants, N=50, 30 women	Perceptions and concerns about a dairy cattle farm and welfare husbandry	Participants were invited to participate in the survey before and after visiting a	(Before visit) Please rank up to three of your top concerns, and indicate why they concern	(Before visit) Fresh food and water (n = 35), pasture and/or outdoor access (n = 28, often with	(After visit) (n=14) Affirmative answer to the question of whether dairy cattle had a good life on this

				dairy farm	you;<br><br>How many days after birth does the dairy calf typically stay with its mom? A) 0 days B) 1 week C) 1 month D) never separated<br><br>(After visit) Write up to five (5) words that come to mind when you think about dairy farming;<br>Do you feel that animals on this farm have a good life? please share any concerns about the quality of life for dairy cattle, in general or on this farm	specific mentions of fresh air and sunshine), gentle and humane care (n = 28), space and freedom to perform behaviors (n = 24), hygiene (n = 10), shelter (n = 9), absence of stress (n = 6), social companions;<br>(After visit)<br>(n = 16 of 50) commented again on existing concerns, e.g.: separation of the calf from the cow, barn space and hygiene,	farm, such that only positive attributes and no concerns were mentioned.<br>(n = 27) mentioned both positive and negative attributes after the farm visit (e.g. cows should be outdoors grazing)<br>(n = 9) who after touring the farm indicated that the dairy cattle did not have good lives (e.g. little space, indoors
(Walker et al., 2014) [73]	Questionnaire via face-to-face	Australia; Convenienc e sampling,	If people believe that animals could	A definition of grief derived from the	1) Can animals experience emotions	1) 96% said yes<br>2) (fear 99%, n = 959; happiness	1) Respondents who did not own a companion animal

	interviews; (year not specified)	N= 999, 478 females The majority of respondents (68%) were Australian nationals, 12% were from New Zealand and the United Kingdom, and the remaining 20% were 'other' nationalities	experience emotions in general and grief in specific	literature on human grief	generally?<br>2) Can animals experience specific emotions (fear, happiness, distress, sadness…)?<br>3) Do animals experience a different intensity of grief to humans?<br>4)Do animals experience a higher or lower intensity of grief than humans?<br>5) Do animals experience grief for a different length of time to humans?<br>6) Do animals experience different emotions to humans?<br>7) In which situations might animals grieve?	96%, n = 937; distress 95%, n = 928; sadness 92%, n = 898; anger 86%, n = 834; love 85%, n = 823; grief 84%, n = 820; and depression 70%, n = 682).<br>3) Yes<br>126/152 (83%)<br>No<br>26/152 (17%)<br>4)Higher<br>11/123 (9%)<br>Lower<br>112/123 (91%)<br>5)Yes<br>122/143 (85%)<br>No<br>21/143 (15%)<br>More<br>4/121 (3%)<br>Less<br>117/121 (97%)<br>6) Yes<br>74/110 (67%)<br>No<br>36/110 (33%)<br>7) 'separation of parent and offspring' (93 and 91%,	were 3.2× as likely to believe animals do not experience emotions (OR = 3.37, P = 0.006) and 4.2× as likely to be uncertain as to whether animals experience emotions (OR = 4.27, P = 0.012) than respondents who did currently own a companion animal.<br>2) Respondents who did not own a companion animal were 1.5 and 2.7× as likely to believe animals do not experience depression (OR = 1.62, P = 0.022) or anxiety (OR = 2.98, P < 0.0001), respectively. They were 1.6, 5.3 and 3.4× as likely to be uncertain whether animals experience depression (OR = 1.74, P = 0.017), distress (OR =

						respectively); ‘separation of other related individuals’ (79 and 74%, respectively); ‘separation of unrelated animals living together’ (78 and 71%, respectively); ‘movement of an animal from his/her home’ (90 and 87%, respectively) and ‘loss of a mating partner’ (93 and 91%, respectively).	5.83, P = 0.001) or anxiety (OR = 3.76, P < 0.0001), respectively; As the age of respondents decreased they became more likely to believe that animals do not experience depression (OR = 0.85, P = 0.02), distress (OR = 0.47, P = 0.007), love (OR = 0.83, P = 0.03) or anxiety (OR = 0.72, P = 0.002) and more likely to be uncertain as to whether animals experience distress (OR = 0.64, P = 0.039). Conversely, as the age of respondents increased, they became more uncertain as to whether animals can experience depression (OR = 1.28, P < 0.0001)

							and more likely to believe that animals do not experience happiness (OR = 1.7, P = 0.005) or sadness (OR = 2, P < 0.0001). Respondents that lived in an urban location were 1.7× as likely to believe that animals do not experience love as those respondents that lived in a suburban or rural location (OR = 1.79, P = 0.019).<br><br>3) Respondents that did own a companion animal were 1.1× as likely as those that did not to believe that 'some' animals can grieve (Z = 2.33, OR = 1.78 (CI = 1.1–2.89), P = 0.02). Age and gender also had a significant influence on respondents' beliefs regarding

							this question and are detailed in McGrath et al (2013). No significant differences were revealed between respondents who currently owned a companion animal and those who did not when questioned as to which animal species (from a pre- defined list) they believed could experience grief ($P > 0.05$).<br><br>4) n.a.<br>5) n.a.<br>6) n.a.<br><br>7) Respondents who did not currently own a companion animal were 1.8× as likely as respondents who did own a companion animal to be uncertain as to whether the

							separation of unrelated animals living together would cause an animal to grieve (OR = 1.88, P = 0.017). No significant difference was found between companion animal owners and non-owners regarding the other four situations posited (P > 0.05).<br>As the age of respondents increased, they became less likely to believe that the separation of related animals (OR = 0.86, P = 0.04) and the loss of a mating partner (OR = 0.74, P = 0.05) would cause an animal to experience grief. However, they also became more uncertain as to whether the

							separation of animals living together would result in grief (OR = 1.18, P = 0.04). Respondents that lived in an urban location were 2.1× as likely to be uncertain as to whether the loss of a mating partner would cause an animal to experience grief than respondents from rural and suburban locations (OR = 2.56, P = 0.03)
(Weible et al., 2016) [74]	Online survey	German citizens, 50% women N=1519	Citizens' perception of modern pig production in Germany	Online survey: to answer two statements, among others, regarding pig husbandry	Citizens' perception of modern pig production	Opponents (22%) are very critical about pig husbandry and have a very low acceptance of modern production systems; Animals do not have enough space to move in modern pig	Younger people and people with better knowledge of agriculture were stronger opponents of intensive pig husbandry. Age is highly significant in the opponents (22%) cluster −0.003 (0.001) towards

						husbandry<br>(Factor 1<br>α¼0.839: 0.664<br>Factor 2<br>α¼0.801: 0.115<br>Factor 3<br>α¼0.630: 0.156<br>Factor 4<br>α¼0.437:<br>−0.059);<br>The pigs feel comfortable in modern stables because they have no other experience<br>(Factor 1<br>α¼0.839: 0.102<br>Factor 2<br>α¼0.801: −0.226<br>Factor 3<br>α¼0.630: 0.836<br>Factor 4<br>α¼0.437: 0.014	pig husbandry
(Weinrich et al., 2014) [75]	Survey, 2013	German consumers N=1009, 50.6% women (Cluster 1 N=281, “quality-conscious”;	Consumer perceptions towards dairy housing systems<br><br>What is the image of fully housed systems?	Respondents were provided with images of cows taken from indoor housing systems and outdoor housing	Agreeability with indoor / fully housing systems for cows	Fully housed systems<br>Animal friendly<br>Very 9.2%<br>Slightly 10.9%<br>Partly 28.2%<br>Cruel towards animals<br>Very 25.9%	The third cluster, the generalists (25.7 %) do not disapprove of indoor-housing as much as the other groups. In this respect they agree particularly with the

		Cluster 2<br>N=not determined, “undecided cluster”;<br>Cluster 3<br>N=257, “generalists”<br>;<br>Cluster 4= not determined, “pasture supporters”		systems: (Figure 1) from typical farm, indoor systems Figure 2 presents images from outdoor systems		Slightly 25.9%.<br>Animal Welfare (Cronbach’s Alpha = 0.644 %of explained variance = 4.624)<br>“I cannot imagine that cows that are living only in a barn can feel well.”<br>0.669<br>“For me, keeping cows indoors year-round is cruelty to animals”.<br>0.653	statements in favour of the indoor-housing system that refer to advantages for animals (e.g., “In indoor systems, animal illness will be noticed faster.”)
(Wernsmann & Wildraut, 2018) [76]	Online questionnaire, 2016	German participants, N=464, 50.4 % women<br>Cluster 1 – uninvolved (N=64)<br><br>Cluster 2 – moderate (N=115).<br><br>Cluster 3 – ethical minded	To gain insights into the participants’ contact to pig farming and included the subjective self-assessments;<br><br>Belief in animal’s mind was inquired (ability to feel emotions and to act conscious)	Sixteen videos in an online experiment varied according to housing conditions (e.g. weight of the pigs, stocking density) and recording conditions (e.g. camera angle, lighting conditions)	Four statements concerning belief in animal mind;<br><br>(Likert-scale) disagree)<br><br>Affective component.<br><br>Cognitive component.<br><br>Behavioural	Pigs have a consciousness and they are aware of what actually happens to them. Strongly disagree 3.2 % Rather disagree 6.9 % I'm not Sure 27.6 % Rather agree 38.4 % Strongly agree 23.9 %;<br><br>Pigs react	(Mean SD)<br>Pigs have a consciousness and they are aware of what actually happens to them. Cluster 1 2.95a (0.33) Cluster 2 4.23b (0.53) Cluster 3 4.18b (0.92) Cluster 4 2.88a (0.97)<br><br>Pigs react automatically,

		(N=181)<br><br>Cluster 4 - pragmatic N=104			component.	automatically, guided by instincts. They do not know what they are doing. Strongly disagree 6.3 % Rather disagree 19.2 % I'm not Sure 34.7 % Rather agree 31.3 % Strongly agree 8.6 % Pigs are able to feel emotions, such as pain, suffering, fear, satisfaction and maternal affection. Strongly disagree 0.2 % Rather disagree 0.2 % I'm not Sure 9.1 % Rather agree 33.8 % Strongly agree 56.7 % Pigs' feelings tend to be less intense than human emotions. Strongly disagree 15.5 %	guided by instincts. They do not know what they are doing. Cluster 1 3.03a (0.35) Cluster 2 3.14a (0.86) Cluster 3 2.68b (1.15) Cluster 4 4.13c (0.48)<br><br>Pigs are able to feel emotions, such as pain, suffering, fear, satis-faction and maternal affection. Cluster 1 3.66a (0.76) Cluster 2 4.50b (0.58) Cluster 3 4.72c (0.55) Cluster 4 4.48b (0.57)<br><br>Pigs' feelings tend to be less intense than human emotions. Cluster 1 3.06a (0.24) Cluster 2 3.13a (0.41) Cluster 3 1.75b (0.79) Cluster 3 3.44c (0.77)<br><br>Videos<br>Cluster 1 is

						Rather disagree 22.4 %<br>I'm not sure 45.7 %<br>Rather agree 14.0 % Strongly agree 2.4 %	‘uninvolved’. The attitudes of the respondents concerning the animal’s mind are undecided. This could be due to the little to no knowledge about modern pig farming. It can be assumed that participants from Cluster 1 do not know how to judge;<br><br>Cluster 2 is ‘moderate. Participants describe themselves as uninformed and have diverse perceptions. They describe pigs as being able to feel emotions and to act consciously, but they are unsure whether pigs are able to act and feel like human beings. Furthermore, the videos were rated

							poorly<br><br>Cluster 3 is ‘ethical minded’.<br>In regard to items concerning pigs’ abilities to feel emotions and to act consciously it can be noted that participants from Cluster 3 have a clear line of argumentation. They strongly agree to the statements concerning pigs’ ability to feel emotions and to act consciously while they disagree that pigs are only guided by instinct and have less intense emotions than human beings. Overall, they have a strong belief in animal mind.<br><br>Cluster 4 is described as ‘pragmatic’. Compared to

							Cluster 1 these respondents are also less critical, but they are interested and well-informed. Especially with regard to belief in animal mind, it can be assumed that participants from Cluster 4 think pragmatically. Respondents attribute the ability to feel emotions but they are rather unsure whether pigs feel emotions less intense than humans.<br><br>A strongly reduced stocking density leads to a better rating.<br><br>Only strong reductions of currently common stocking densities have the potential to lead to better evaluations of pig

							fattening. Overall, it is confirmed that for citizens the space to move is an important animal welfare criteria
(Widmar et al., 2017) [77]	Online survey, 2015	U.S. residents, N=1201, 610 women	To identify respondents' perceptions of humane animal treatment relative to their shopping preferences	No info.	Tail docking; Dehorning (Likert scale)	Tail docking and dehorning were viewed as more detrimental to dairy cattle than the other practices considered	Tail docking Female (SE) −0.6450 (0.1471)<br><br>Dehorning (SE) −0.6333 (0.1404)
(Yunes et al., 2017) [78]	Survey; 2014-2015	Brazilian citizens, N=479, 53% women	To explore the beliefs and attitudes of Brazilians towards systems that are associated with restriction of movement	Two pages with two images each were presented to participants, with three questions that were repeated after each set of pictures of different production systems: free-range beef cattle and beef cattle in intensive open-	In your opinion, which of these situations is the most common in commercial production in Brazil?	Participants showed limited awareness of animal food production systems and practices used in Brazil, but were critical of perceived outcomes of practices and systems on the quality of the products and in regards to the lives led by the animals (e.g.,	79% considered farm animals are not well-treated; For 39% farm animal welfare was a major concern; How informed participants considered themselves to be about animal production, 7% very informed, 31% somewhat informed, 34% intermediate, 20% somewhat uninformed, 8%

				air confinement (feedlot); free-range broilers or broilers in intensive indoor confinement; free-range laying hens or layers in battery cages; free-range farrowing sows or sows in farrowing cages; and, group housed gestating sows or gestating sows in individual cages		suffering, freedom)	totally uninformed; The proportion of participants that believed that farm animals in Brazil are reared in intensive confinement or caged systems varied by animal industry: 23% for beef cattle, 82% for poultry, 81% for laying hens, 56% for gestating sows, and 63% for farrowing sows
(Yunes et al., 2018) [79]	Online survey; 2014-2015	Brazilian participants N=173 (considered NotALP - participants not associated with	Views of Brazilians on gestation stall housing	Participants could choose to access a 90-s video that depicted sows living in individual or group stalls, and also	What is your position regarding housing gestating sows in individual stalls?	Participants were generally opposed to restrictive housing for sows who despite considering themselves uninformed about	What is your position regarding housing gestating sows in individual stalls?<br><br>Rejected (N=151)<br>Supported (N=14)<br>Indifferent (N=8)

		livestock production) (not considered ALP - participants associated with livestock production excluded N=176)		showed potential behaviour problems associated to each system (eg, stereotyped behaviours in sows housed in stalls and scenes of social tension in group-housed sows) + text pointing the pros for individual stalls for the industry and arguments pointing animal suffering		swine production, and being unaware of the widespread prevalence of gestation stalls for sows in Brazilian farms, were overwhelming in their rejection of this system	Animal welfare<br>Rejected (87%)<br>Supported (53%)<br>Indifferent (29%)<br><br>Number and percentage of participants who rejected, supported or were indifferent to the practice of housing gestating sows in stalls:<br><br>Reject (N=151)<br>Support (N=14)<br>Indifferent (N=8)<br>Female<br>Reject 108 Support 7 Indifferent 3<br>Male<br>Reject 43<br>Support 7<br>Indifferent 5<br><br>Information about swine production:<br>Very informed<br>Reject 21 Support 1 Indifferent 0<br>Somewhat informed<br>Reject 28

							Support 3<br>Indifferent 2<br>Intermediate Reject 25<br>Support 3<br>Indifferent 0<br>Somewhat uninformed Reject 36 Support 4<br>Indifferent 1<br>Totally uninformed Reject 41<br>Support 3<br>Indifferent 5

References

1. Aluwé, M.; Heyrman, E.; Almeida, J.M.; Babol, J.; Battacone, G.; Čítek, J.; Furnols, M.F.I.; Getya, A.; Karolyi, D.; Kostyra, E.; et al. Exploratory Survey on European Consumer and Stakeholder Attitudes towards Alternatives for Surgical Castration of Piglets. *Animals* **2020**, *10*, 1–26, doi:10.3390/ani10101758.

2. Anderson, E.C.; Barrett, L.F. Affective Beliefs Influence the Experience of Eating Meat. *PLoS One* **2016**, *11*, doi:10.1371/journal.pone.0160424.

3. Hartmann, C.; Siegrist, M.; Cardoso, C.S.; von Keyserlingk, M.A.G.; Hötzel, M.J.M.J.; Bray, H.J.; Ankeny, R.A.; Leach, S.; Piazza, J.; Loughnan, S.; et al. When Closing the Human-Animal Divide Expands Moral Concern: The Importance of Framing. *Appetite* **2018**, *11*, 1–18, doi:10.1186/s13028-020-00522-6.

4. Bastian, B.; Loughnan, S.; Haslam, N.; Radke, H.R.M. Don’t Mind Meat? The Denial of Mind to Animals Used for Human Consumption. *Personal. Soc. Psychol. Bull.* **2012**, *38*, 247–256, doi:10.1177/0146167211424291.

5. van Beirendonck, S.; Driessen, B.; Geers, R. Belgian Consumers’ Opinion on Pork Consumption Concerning Alternatives for Unanesthetized Piglet Castration. *J. Agric. Environ. Ethics* **2013**, *26*, 259–272, doi:10.1007/s10806-012-9378-y.

6. Benningstad, N.C.G.; Kunst, J.R. Dissociating Meat from Its Animal Origins: A Systematic Literature Review. *Appetite* 2020, *147*.

7. Bergstra, T.J.; Gremmen, B.; Stassen, E.N. Moral Values and Attitudes Toward Dutch Sow Husbandry. *J. Agric. Environ. Ethics* **2015**, *28*, 375–401, doi:10.1007/s10806-015-9539-x.

8. Bergstra, T.; Hogeveen, H.; Erno Kuiper, W.; Oude Lansink, A.G.J.M.; Stassen, E.N. Attitudes of Dutch Citizens toward Sow Husbandry with Regard to Animals, Humans, and the Environment. *Anthrozoos* **2017**, *30*, 195–211, doi:10.1080/08927936.2017.1310985.

9. Bratanova, B.; Loughnan, S.; Bastian, B. The Effect of Categorization as Food on the Perceived Moral Standing of Animals. *Appetite* **2011**, *57*, 193–196, doi:10.1016/j.appet.2011.04.020.

10. Bray, H.J.; Ankeny, R.A. Happy Chickens Lay Tastier Eggs: Motivations for Buying Free-Range Eggs in Australia. *Anthrozoos* **2017**, *30*, 213–226, doi:10.1080/08927936.2017.1310986.

11. Buddle, E.A.; Bray, H.J.; Ankeny, R.A. Why Would We Believe Them? Meat Consumers' Reactions to Online Farm Animal Welfare Activism in Australia. *Commun. Res. Pract.* **2018**, *4*, 246–260, doi:10.1080/22041451.2018.1451209.

12. Buddle, E.A.; Bray, H.J.; Ankeny, R.A. "I Feel Sorry for Them": Australian Meat Consumers' Perceptions about Sheep and Beef Cattle Transportation. *Animals* **2018**, *8*, doi:10.3390/ani8100171.

13. Busch, G.; Gauly, S.; Von Meyer-Höfer, M.; Spiller, A. Does Picture Background Matter? People's Evaluation of Pigs in Different Farm Settings. *PLoS One* **2019**, *14*, 1–19, doi:10.1371/journal.pone.0211256.

14. Cardoso, C.S.; Von Keyserlingk, M.A.G.; Hötzel, M.J. Brazilian Citizens: Expectations Regarding Dairy Cattle Welfare and Awareness of Contentious Practices. *Animals* **2017**, *7*, doi:10.3390/ani7120089.

15. Christoph-Schulz, I.; Rovers, A.K. German Citizens' Perception of Fattening Pig Husbandry—Evidence from a Mixed Methods Approach. *Agric.* **2020**, *10*, 1–20, doi:10.3390/agriculture10080342.

16. Clark, B.; Panzone, L.A.; Stewart, G.B.; Kyriazakis, I.; Niemi, J.K.; Latvala, T.; Tranter, R.; Jones, P.; Frewer, L.J. Consumer Attitudes towards Production Diseases in Intensive Production Systems. *PLoS One* **2019**, *14*, doi:10.1371/journal.pone.0210432.

17. Coleman, G.; Jongman, E.; Greenfield, L.; Hemsworth, P. Farmer and Public Attitudes Toward Lamb Finishing Systems. *J. Appl. Anim. Welf. Sci.* **2016**, *19*, 198–209, doi:10.1080/10888705.2015.1127766.

18. Connor, M.; Cowan, S.L. Consumer Evaluation of Farm Animal Mutilations. *Res. Vet. Sci.* **2020**, *128*, 35–42,

doi:10.1016/j.rvsc.2019.10.006.

19. de Haas, E.N.; Oliemans, E.; van Gerwen, M.A.A.M. The Need for an Alternative to Culling Day-Old Male Layer Chicks: A Survey on Awareness, Alternatives, and the Willingness to Pay for Alternatives in a Selected Population of Dutch Citizens. *Front. Vet. Sci.* **2021**, *8*, doi:10.3389/fvets.2021.662197.

20. de Jonge, J.; van Trijp, H.C.M. The Impact of Broiler Production System Practices on Consumer Perceptions of Animal Welfare. *Poult. Sci.* **2013**, *92*, 3080–3095, doi:10.3382/ps.2013-03334.

21. Dowsett, E.; Semmler, C.; Bray, H.; Ankeny, R.A.; Chur-Hansen, A. Neutralising the Meat Paradox: Cognitive Dissonance, Gender, and Eating Animals. *Appetite* **2018**, *123*, 280–288, doi:10.1016/j.appet.2018.01.005.

22. Estévez-Moreno, L.X.; María, G.A.; Sepúlveda, W.S.; Villarroel, M.; Miranda-de la Lama, G.C. Attitudes of Meat Consumers in Mexico and Spain about Farm Animal Welfare: A Cross-Cultural Study. *Meat Sci.* **2021**, *173*, 108377, doi:10.1016/j.meatsci.2020.108377.

23. Estévez-Moreno, L.X.; Miranda-de la Lama, G.C.; Miguel-Pacheco, G.G. Consumer Attitudes towards Farm Animal Welfare in Argentina, Chile, Colombia, Ecuador, Peru and Bolivia: A Segmentation-Based Study. *Meat Sci.* **2022**, *187*, doi:10.1016/j.meatsci.2022.108747.

24. Fredriksen, B.; Johnsen, A.M.S.; Skuterud, E. Consumer Attitudes towards Castration of Piglets and Alternatives to Surgical Castration. *Res. Vet. Sci.* **2011**, *90*, 352–357, doi:10.1016/j.rvsc.2010.06.018.

25. Frewer, L.J.; Kole, A.; van de Kroon, S.M.; de Lauwere, C. Consumer Attitudes towards the Development of Animal-Friendly Husbandry Systems. *J. Agric. Environ. Ethics* **2005**, *18*, 345–367, doi:10.1007/s10806-005-1489-2.

26. Fuseini, A.; Knowles, T.G. The Ethics of Halal Meat Consumption: Preferences of Consumers in England According to the Method of Slaughter. *Vet. Rec.* **2020**, *186*, doi:10.1136/vr.105287.

27. Fuseini, A.; Wotton, S.B.; Hadley, P.J.; Knowles, T.G. The Perception and Acceptability of Pre-Slaughter and Post-Slaughter Stunning for Halal Production: The Views of UK Islamic Scholars and Halal Consumers. *Meat Sci.* **2017**, *123*, 143–150, doi:10.1016/j.meatsci.2016.09.013.

28. Gremmen, B.; Bruijnis, M.R.N.; Blok, V.; Stassen, E.N. A Public Survey on Handling Male Chicks in the Dutch Egg Sector. *J. Agric. Environ. Ethics* **2018**, *31*, 93–107, doi:10.1007/s10806-018-9712-0.

29. Hartmann, C.; Siegrist, M. Our Daily Meat: Justification, Moral Evaluation and Willingness to Substitute. *Food Qual. Prefer.* **2020**, *80*, doi:10.1016/j.foodqual.2019.103799.

30. Heid, A.; Hamm, U. Consumer Attitudes Towards Alternatives to Piglet Castration Without Pain Relief in Organic Farming: Qualitative Results from Germany. *J. Agric. Environ. Ethics* **2012**, *25*, 687–706, doi:10.1007/s10806-011-9350-2.

31. Herrewijn, L.; De Groeve, B.; Cauberghe, V.; Hudders, L. VR Outreach and Meat Reduction Advocacy: The Role of Presence, Empathic Concern and Speciesism in Predicting Meat Reduction Intentions. *Appetite* **2021**, *166*, 105455, doi:10.1016/j.appet.2021.105455.

32. Hötzel, M.J.; Cardoso, C.S.; Roslindo, A.; von Keyserlingk, M.A.G. Citizens' Views on the Practices of Zero-Grazing and Cow-Calf Separation in the Dairy Industry: Does Providing Information Increase Acceptability? *J. Dairy Sci.* **2017**, *100*, 4150–4160, doi:10.3168/jds.2016-11933.

33. Hötzel, M.J.; Yunes, M.C.; Vandresen, B.; Albernaz-Gonçalves, R.; Woodroffe, R.E. On the Road to End Pig Pain: Knowledge and Attitudes of Brazilian Citizens Regarding Castration. *Animals* **2020**, *10*, 1–22, doi:10.3390/ani10101826.

34. Syazwani, N.; Jalil, A.; Tawde, A.V.; Zito, S.; Sinclair Id, M.; Fryer, C.; Idrus, Z.; Phillips, C.J.C. Attitudes of the Public towards Halal Food and Associated Animal Welfare Issues in Two Countries with Predominantly Muslim and Non-Muslim Populations. **2018**, doi:10.1371/journal.pone.0204094.

35. Krystallis, A.; de Barcellos, M.D.; Kügler, J.O.; Verbeke, W.; Grunert, K.G. Attitudes of European Citizens towards Pig Production Systems. *Livest. Sci.* **2009**, *126*, 46–56, doi:10.1016/j.livsci.2009.05.016.

36. Kühl, S.; Gauly, S.; Spiller, A. Analysing Public Acceptance of Four Common Husbandry Systems for Dairy Cattle Using a Picture-Based Approach. *Livest. Sci.* **2019**, *220*, 196–204, doi:10.1016/j.livsci.2018.12.022.

37. Kupsala, S.; Vinnari, M.; Jokinen, P.; Räsänen, P. Public Perceptions of Mental Capacities of Nonhuman Animals. *Soc. Anim.* **2014**, *24*, 445–466, doi:10.1163/15685306-12341423.

38. Kupsala, S.; Vinnari, M.; Jokinen, P.; Räsänen, P. Citizen Attitudes to Farm Animals in Finland: A Population-Based Study. *J. Agric. Environ. Ethics* **2015**, *28*, 601–620, doi:10.1007/s10806-015-9545-z.

39. Miranda-de la Lama, G.C.; Estévez-Moreno, L.X.; Sepúlveda, W.S.; Estrada-Chavero, M.C.; Rayas-Amor, A.A.; Villarroel, M.; María, G.A. Mexican Consumers' Perceptions and Attitudes towards Farm Animal Welfare and Willingness to Pay for Welfare Friendly Meat Products. *Meat Sci.* **2017**, *125*, 106–113, doi:10.1016/j.meatsci.2016.12.001.

40. Leach, S.; Piazza, J.; Loughnan, S.; Sutton, R.M.; Kapantai, I.; Dhont, K.; Douglas, K.M. Unpalatable Truths: Commitment to Eating Meat Is Associated with Strategic Ignorance of Food-Animal Minds. *Appetite* **2022**, *171*, 105935, doi:10.1016/j.appet.2022.105935.

41. Teixeira, D.L.; Larraín, R.; Melo, O.; Hötzel, M.J. Public Opinion towards Castration without Anaesthesia and Lack of Access to Pasture in Beef Cattle Production. *PLoS One* **2018**, *13*, 1–16, doi:10.1371/journal.pone.0190671.

42. Loughnan, S.; Haslam, N.; Bastian, B. The Role of Meat Consumption in the Denial of Moral Status and Mind to Meat Animals. *Appetite* **2010**, *55*, 156–159, doi:10.1016/j.appet.2010.05.043.

43. Malek, L.; Umberger, W.J.; Rolfe, J. Segmentation of Australian Meat Consumers on the Basis of Attitudes Regarding Farm Animal Welfare and the Environmental Impact of Meat Production. *Anim. Prod. Sci.* **2018**, *58*, 424–434, doi:10.1071/AN17058.

44. McGrath, N.; Walker, J.; Nilsson, D.; Phillips, C. Public Attitudes towards Grief in Animals. *Anim. Welf.* **2013**, *22*, 33–47, doi:10.7120/09627286.22.1.033.

45. Monteiro, C.A.; Pfeiler, T.M.; Patterson, M.D.; Milburn, M.A. The Carnism Inventory: Measuring the Ideology of Eating Animals. *Appetite* **2017**, *113*, 51–62, doi:10.1016/j.appet.2017.02.011.

46. Peden, R.S.E.; Camerlink, I.; Boyle, L.A.; Loughnan, S.; Akaichi, F.; Turner, S.P. Belief in Pigs’ Capacity to Suffer: An Assessment of Pig Farmers, Veterinarians, Students, and Citizens. *Anthrozoos* **2020**, *33*, 21–36, doi:10.1080/08927936.2020.1694304.

47. Cardoso, Clarissa S.; von Keyserlingk, Marina A. G.; Hötzel, M.J. Brazilian Citizens: Expectations Regarding Dairy Cattle Welfare and Awareness of Contentious Practices. *Animals* **2017**, *7*, 1–15, doi:10.3390/ani7120089.

48. Piazza, J.; Loughnan, S. When Meat Gets Personal, Animals’ Minds Matter Less: Motivated Use of Intelligence Information in Judgments of Moral Standing. *Soc. Psychol. Personal. Sci.* **2016**, *7*, 867–874, doi:10.1177/1948550616660159.

49. Piazza, J.; McLatchie, N.; Olesen, C. Are Baby Animals Less Appetizing? Tenderness toward Baby Animals and Appetite for Meat. *Anthrozoos* **2018**, *31*, 319–335, doi:10.1080/08927936.2018.1455456.

50. de Queiroz, R.G.; de Faria Domingues, C.H.; Canozzi, M.E.A.; Garcia, R.G.; Ruviaro, C.F.; Barcellos, J.O.J.; Borges, J.A.R. How Do Brazilian Citizens Perceive Animal Welfare Conditions in Poultry, Beef, and Dairy Supply Chains? *PLoS One* **2018**, *13*, 1–9, doi:10.1371/journal.pone.0202062.

51. Rice, M.; Hemsworth, L.M.; Hemsworth, P.H.; Coleman, G.J. The Impact of a Negative Media Event on Public Attitudes Towards Animal Welfare in the Red Meat Industry. **2020**, *10*, 619, doi:10.3390/ani10040619.

52. Robbins, J.A.; Roberts, C.; Weary, D.M.; Franks, B.; von Keyserlingk, M.A.G. Factors Influencing Public Support for Dairy Tie Stall Housing in the U.S. *PLoS One* **2019**, *14*, doi:10.1371/JOURNAL.PONE.0216544.

53. Rovers, A.; Sonntag, W.I.; Brümmer, N.; Christoph-Schulz, I. *Number 4 Future Options for Animal Husbandry*; 2018; Vol. 67;.

54. Ryan, E.B.; Fraser, D.; Weary, D.M. Public Attitudes to Housing Systems for Pregnant Pigs. *PLoS One* **2015**, *10*, 1–14, doi:10.1371/journal.pone.0141878.

55. Sato, P.; Hötzel, M.J.; Von Keyserlingk, M.A.G. American Citizens' Views of an Ideal Pig Farm. *Animals* **2017**, *7*, doi:10.3390/ani7080064.

56. Sødring, M.; Nafstad, O.; Håseth, T.T. Change in Norwegian Consumer Attitudes towards Piglet Castration: Increased Emphasis on Animal Welfare. *Acta Vet. Scand.* **2020**, *62*, doi:10.1186/s13028-020-00522-6.

57. Sonntag, W.I.; Spiller, A. Measuring Public Concerns? Developing a Moral Concerns Scale Regarding Non-Product Related Process and Production Methods. *Sustain.* **2018**, *10*, doi:10.3390/su10051375.

58. Soriano, V.S.; Phillips, C.J.C.; Taconeli, C.A.; Fragoso, A.A.H.; Molento, C.F.M. Mind the Gap: Animal Protection Law and Opinion of Sheep Farmers and Lay Citizens Regarding Animal Maltreatment in Sheep Farming in Southern Brazil. *Animals* **2021**, *11*, doi:10.3390/ani11071903.

59. Tamioso, P.R.; Rucinque, D.S.; Miele, M.; Boissy, A.; Molento, C.F.M. Perception of Animal Sentience by Brazilian and French Citizens: The Case of Sheep Welfare and Sentience. *PLoS One* **2018**, *13*, doi:10.1371/journal.pone.0200425.

60. Tawse, J. Consumer Attitudes towards Farm Animals and Their Welfare: A Pig Production Case Study. *Biosci. Horizons* **2010**, *3*, 156–165, doi:10.1093/biohorizons/hzq020.

61. Tian, Q.; Hilton, D.; Becker, M. Confronting the Meat Paradox in Different Cultural Contexts: Reactions among Chinese and French Participants. *Appetite* **2016**, *96*, 187–194, doi:10.1016/j.appet.2015.09.009.

62. Tiplady, C.M.; Walsh, D.A.B.; Phillips, C.J.C. Public Response to Media Coverage of Animal Cruelty. *J. Agric. Environ. Ethics* **2013**, *26*, 869–885, doi:10.1007/s10806-012-9412-0.

63. Tomasevic, I.; Bahelka, I.; Čítek, J.; Čandek-Potokar, M.; Djekić, I.; Getya, A.; Guerrero, L.; Ivanova, S.; Kušec, G.; Nakov, D.; et al. Attitudes and Beliefs of Eastern European Consumers towards Animal Welfare. *Animals* **2020**, *10*, 1–18, doi:10.3390/ani10071220.

64. Tomasevic, I.; Bahelka, I.; Čandek-Potokar, M.; Čítek, J.; Djekić, I.; Djurkin Kušec, I.; Getya, A.; Guerrero, L.; Iordăchescu, G.; Ivanova, S.; et al. Attitudes and Beliefs of Eastern European Consumers towards Piglet Castration and Meat from Castrated Pigs. *Meat Sci.* **2020**, *160*, doi:10.1016/j.meatsci.2019.107965.

65. Tonsor, G.T.; Olynk, N.; Wolf, C. Consumer Preferences for Animal Welfare Attributes: The Case of Gestation Crates. *J. Agric. Appl. Econ.* **2009**, *41*, 713–730, doi:10.1017/s1074070800003175.

66. Vandresen, B.; Hötzel, M.J. "Mothers Should Have Freedom of Movement"—Citizens' Attitudes Regarding Farrowing Housing Systems for Sows and Their Piglets. *Animals* **2021**, *11*, doi:10.3390/ani11123439.

67. Vanhonacker, F.; Verbeke, W. Consumer Response to the Possible Use of a Vaccine Method to Control Boar Taint v. Physical Piglet Castration with Anaesthesia: A Quantitative Study in Four European Countries. *Animal* 2011, *5*, 1107–1118.

68. Vanhonacker, F.; Verbeke, W. *Belgian Consumers' Attitude towards Surgical Castration and Immunocastration of Piglets*; 2009;

69. Vanhonacker, F.; Verbeke, W.; Van Poucke, E.; Buijs, S.; Tuyttens, F.A.M. Societal Concern Related to Stocking Density, Pen Size and Group Size in Farm Animal Production. *Livest. Sci.* **2009**, *123*, 16–22, doi:10.1016/j.livsci.2008.09.023.

70. Vanhonacker, F.; Tuyttens, F.A.M.; Verbeke, W. Belgian Citizens' and Broiler Producers' Perceptions of Broiler Chicken Welfare in Belgium versus Brazil. *Poult. Sci.* **2016**, *95*, 1555–1563, doi:10.3382/ps/pew059.

71. Ventura, B.A.; von Keyserlingk, M.A.G.; Schuppli, C.A.; Weary, D.M. Views on Contentious Practices in Dairy Farming: The Case of Early Cow-Calf Separation. *J. Dairy Sci.* **2013**, *96*, 6105–6116, doi:10.3168/jds.2012-6040.

72. Ventura, B.A.; Von Keyserlingk, M.A.G.; Wittman, H.; Weary, D.M. What Difference Does a Visit Make? Changes in Animal Welfare Perceptions after Interested Citizens Tour a Dairy Farm. *PLoS One* **2016**, *11*, doi:10.1371/journal.pone.0154733.

73. Walker, J.K.; McGrath, N.; Handel, I.G.; Waran, N.K.; Phillips, C.J.C. Does Owning a Companion Animal Influence the Belief That Animals Experience Emotions Such as Grief? *Anim. Welf.* **2014**, *23*, 71–79, doi:10.7120/09627286.23.1.071.

74. Weible, D.; Christoph-Schulz, I.; Salamon, P.; Zander, K. Citizens' Perception of Modern Pig Production in Germany: A Mixed-Method Research Approach. *Br. Food J.* **2016**, *118*, 2014–2032, doi:10.1108/BFJ-12-2015-0458.

75. Weinrich, R.; Kühl, S.; Zühlsdorf, A.; Spiller, A. *Consumer Attitudes in Germany towards Different Dairy Housing Systems and Their Implications for the Marketing of Pasture Raised Milk*; 2014; Vol. 17;.

76. Wernsmann, A.; Wildraut, C.; Südwestfalen, F.; Von Meyer-Höfer, M.; Mergenthaler, M. *Perception and Evaluation of a Pig Fattening Pen Based on Film Material in an Online Survey Experiment with German Citizens*; 2018; Vol. 67;.

77. Olynk Widmar, N.; Morgan, C.J.; A. Wolf, C.; A. Yeager, E.; Dominick, S.R.; Croney, C.C. US Resident Perceptions of Dairy Cattle

Management Practices. *Agric. Sci.* **2017**, *08*, 645–656, doi:10.4236/as.2017.87049.

78. Yunes, M.C.; Von Keyserlingk, M.A.G.; Hötzel, M.J. Brazilian Citizens' Opinions and Attitudes about Farm Animal Production Systems. *Animals* **2017**, *7*, doi:10.3390/ani7100075.

79. Yunes, M.C.; Keyserlingk, M.A.G.V.; Hötzel, M.J. Restricting the Ability of Sows to Move: A Source of Concern for Some Brazilians. *Anim. Welf.* **2018**, *27*, 379–392, doi:10.7120/09627286.27.4.379.
