# Supplementary material for: Consumers’ Attitudes towards Animal Suffering: A Systematic Review on Awareness, Willingness and Dietary Change
Source: Int J Environ Res Public Health. 2022 Dec 6;19(23):16372. doi: 10.3390/ijerph192316372 (PMC9741386; doi:10.3390/ijerph192316372)
Supplement: Supplementary file 1 [file ijerph-19-16372-s001.zip › Table Change (2).pdf]

Supplementary Materials

Table S3 - Consumers that have reduced or halted meat consumption because of animal suffering in animal agriculture

**Authors, year**	**Design; year data collected**	**Country; sample**	**Main research question**	**(Type of?) Information provided on animal suffering**	**Outcome measure:**		
					**Question or dependent variable**	**Response or finding**	**Effects of covariates**
(Dijkstra & Rotelli, 2022) [1]	Online survey; 2019	Italian participants, Environment condition N=38 Animal condition N=23 Health condition N=35 Control condition N=41 (Total N=139)	Lowering red meat and processed meat consumption with environmental, animal Welfare, and health arguments	Three types of persuasive arguments were worded in a separate text, that argued one-sidedly on the negative effects on red and processed meat:<br><br>Environmental impacts;<br><br>Some animal farming conditions (suffering); Health impacts of red and processed meat and	Consequences of meat consumption: Environment; Animal welfare; Health; Text on mustard (control condition)<br><br>(Liker-scale) "Are you planning to decrease your red meat/processe d meat consumption in the next 2 months?"	Persuasive messages only influenced red meat consumption but not processed meat consumption;<br><br>Health arguments were the most effective, and environmental arguments the least effective. Animal arguments fell somewhere in between. In participants with relatively low pre-test meat consumption,	Post-test red meat and processed meat consumption: the main effect of condition was not significant: for red meat consumption, F(3,134) = 1.03, p = 0.38, ^{2} = 0.023, for processed meat consumption, F(3,134) = 1.27, p = 0.29, ^{2} = 0.028;<br><br>Interaction Effects: red meat consumption as the dependent variable, the

				benefits in reducing its consumption; The text included eight pictures; five with surgery scenes and three with close-ups of biological cells.<br><br>The control conditions presented a text on the production of mustard, how it is grown and what it is used for. The text included three pictures of the mustard plant and seeds. Each outcome text was complimented with a text designed to influence other possible determinants		there were no significant differences in red meat consumption post-test. This segment already consumed little or no meat	interaction between pre-test meat consumption and condition was significant, $F(3,125) = 3.08$, $p = 0.03$, $^2 = 0.07$, while the interaction between pre-test intention and condition approached significance, $F(3,125) = 2.27$, $p = 0.084$, $^2 = 0.052$. In the model with processed meat consumption as the dependent variable, both interactions were not significant ($p > 0.22$);<br><br>Pre-Test Meat Consumption as a Moderator: When pre-test consumption was modeled as low,

				of meat consumption. This text was the same for all four conditions. The social norm was addressed by stating that more and more people lower their meat consumption			the main effect of condition was not significant, $F(3,125) = 0.48$, $p = 0.70$, $^2 = 0.011$; Contrast analyses showed that red meat consumption in the health condition (M = 2.92) was significantly lower than in the environment condition (M = 4.27, p = 0.001, 95%; Red meat consumption in the animal condition (M = 3.27) was significantly lower compared to the environment condition (M = 4.27, p = 0.029, 95% CI difference −1.91 to −0.10);

							The animal condition, the correlation approached significance, r(20) = 0.41, p = 0.056, while in the health condition, the correlation was not significant, r(32) = 0.15, p = 0.42;<br><br>In participants with relatively high pre-test meat consumption, health arguments led to lower red meat consumption, compared to environmental arguments and to the control condition;<br><br>The control condition shows that health arguments were effective;

							they lowered red meat consumption compared to when people received no arguments. In the environmental argument condition, red meat consumption was higher compared to the control condition
(Haile et al., 2021) [2]	Online survey, 2018-2020	U.S. students, N=338	Can a randomized-controlled trial of pro-vegan animal-welfare pamphlets with students present any significant effects in meat reduction?	A treatment group received an animal-advocacy pamphlet (by Vegan Outreach). The pamphlet discusses the impact of factory farming and the conditions under which farm animals are treated. The pamphlet	0) Actual food purchase in the campus cafeteria (categories: a) beef, b) poultry/fish, c) vegetarian, d) meat)<br><br>1) Has your diet changed over the last month?<br>2) If your diet changed over the last month,	0) There is no statistically significant effect of the treatment on food choice at the cafeteria for any of a, b, c, d, outcomes and in any of the time windows (during the semester of the intervention and after the semester of the intervention)..<br><br>Vegan/vegetarian	0) Men significantly decrease their consumption of poultry or fish by 2.4 percentage points (5.2%) and increase their consumption of vegetarian/vegan meals by roughly the same magnitude, 2.3 percentage points (10.6%). Overall, meat consumption for

				also contains information on how to eat less meat, i.e., discussions about the health benefits of eating a plant-based diet	which of the following are reasons you think contributed to the change? (check all that apply)<br>3) If a leafleter gave you leaflet(s), do you think the leaflet(s) affected you?<br>4) Reading the leaflet(s) taught me about (choose all the reasons that apply)<br>5) After reading the leaflet I thought more about (choose all the reasons that apply)	diet<br>(Means) Control 0.178 (0.383)Treatment 0.121 (0.326) (Difference) Treatment - Control 0.057 (0.254)<br><br>Meat reduction diet (Means) Control 0.210 (0.407) Treatment 0.157 (0.364) (Difference) Treatment - control -0.052 (0.309)<br><br>Has changed diet (Means) Control 0.141 (0.348) Treatment 0.131 (0.337) (Difference) Treatment - control -0.010 (0.827)<br><br>Reason diet changed: Animal cruelty and ethics	men falls by the same magnitude as the decline in poultry/fish, 2.4 percentage points (3.6%). Women, significantly reduce beef consumption by 1.5 percentage points (12.5%). Poultry and fish consumption increases, though insignificantly, which explains why overall meat consumption does not fall for women.<br>There were statistically significant effects by gender within the semester of the intervention, but not afterwards. The reductions in beef and poultry/fish for men and women,

						(Means) Control 0.081 (0.274) Treatment 0.026 (0.159) (Difference) Treatment - Control -0.056 (0.496)<br><br>The results show that the animal-advocacy pamphlets had no detectable aggregate effects in the short or long term. The treatment effects of reducing meat in the first semester were rejected by 2.6 percentage points or larger (CI = [−0.026, 0.006]), in the second semester by 2.1 percentage points or larger (CI = [−0.021, 0.023]), and over both semesters by 1.9 percentage points or larger	respectively, are statistically significant in the second month after the intervention.<br><br>During the semester of the intervention, men significantly decrease their consumption of poultry or fish by 2.4 percentage points (5.2%) and increase their consumption of vegetarian/vegan meals by roughly the same magnitude, 2.3 percentage points (10.6%), suggesting substitution from meat to vegetarian/vegan meals. Overall, meat consumption for men falls by the

						(CI = [−0.019, 0.013]), with 95% confidence	same magnitude as the decline in poultry/fish, 2.4 percentage points (3.6%). Women, in contrast, significantly reduce beef consumption by 1.5 percentage points (12.5%). Poultry and fish consumption increases, though insignificantly, which explains why overall meat consumption does not fall for women. This finding suggests substitution from red meat (beef) to poultry/fish for women
(Niemyjska et al., 2018) [3]	Online survey;	Polish participants Study 1 N=306, 224 women Study 2	To test whether individual differences in anthropomorp hism are	No info.	To what extent do cows have intentions?<br><br>Animal anthropomorp	Most participants who reported that they had reduced their meat consumption or refrained from	

		N=307, 194 women	related to empathic connection with non-human animals and hence decreased meat consumption		hism;<br><br>General anthropomorphism;<br><br>Participants reported their consumption of meat and animal products:<br><br>(Likert-scale) I often eat meat; I sometimes eat meat; I rarely eat meat; I do not eat meat, but I do eat zoonotic products (like milk or cheese); I eat neither meat nor zoonotic products	eating meat had done so for less than a year (24.5% of the sample). This decision was most strongly related to animal harm: r(114) = .50, p < .001, protecting the environment and its resources: r(114) = .28, p = .003, and the price of meat: r(112) = -.25, p = .009<br>When controlling for empathic concern for animals the direct effect of animal anthropomorphism on eating meat was weaker, but still significant (c' = 0.28, SE = 0.06, t = 4.54, p < .001, 95%CI [0.16, 0.41]). The indirect effect of empathy with animals was	

						significant, 95% boot CI [0.02, 0.09], ab= 0.05, boot SE = 0.02<br><br>Data support the hypothesized model of relationships between anthropomorphism, empathy and importance of harm to animals to dietary choices and decreased meat consumption. More specifically, higher levels of animal anthropomorphism predict greater empathic concern for animals	

References

1. Dijkstra, A.; Rotelli, V. Lowering Red Meat and Processed Meat Consumption With Environmental, Animal Welfare, and Health Arguments in Italy: An Online Experiment. *Front. Psychol.* **2022**, *13*, doi:10.3389/fpsyg.2022.877911.
2. Haile, M.; Jalil, A.; Tasoff, J.; Vargas Bustamante, A. Changing Hearts and Plates: The Effect of Animal-Advocacy Pamphlets on Meat Consumption. *Front. Psychol.* **2021**, *12*, doi:10.3389/fpsyg.2021.668674.
3. Niemyjska, A.; Cantarero, K.; Byrka, K.; Bilewicz, M. Too Humanlike to Increase My Appetite: Disposition to Anthropomorphize Animals Relates to Decreased Meat Consumption through Empathic Concern. *Appetite* **2018**, *127*, 21–27, doi:10.1016/j.appet.2018.04.012.
