# Supplementary material for: Consumers’ Attitudes towards Animal Suffering: A Systematic Review on Awareness, Willingness and Dietary Change
Source: Int J Environ Res Public Health. 2022 Dec 6;19(23):16372. doi: 10.3390/ijerph192316372 (PMC9741386; doi:10.3390/ijerph192316372)
Supplement: Supplementary file 1 [file ijerph-19-16372-s001.zip › Table Willingness (1).pdf]

Supplementary Materials

**Table S2 - Consumers' willingness to change diet because of animal suffering in animal agriculture**							
**Authors, year**	**Design; year data collected**	**Country; sample**	**Main research question**	**(Type of?) Information provided on animal suffering**	**Outcome measure:**		
					**Question or dependent variable**	**Response or finding**	**Effects of covariates**
(Cordts et al., 2014) [1]	Online survey; 2013	German population, N=590, 284 women	Media coverage of certain negative attributes of meat consumption can potentially affect demand for meat	Respondents access to "newspaper article" about animal welfare	If fictitious "newspaper articles" describing negative effects of meat consumption – on animal welfare can motivate participants to reduce meat	Respondents believing to reduce meat consumption in future before and after having read one out of the four "newspaper articles" (n=150, 82 women)	Women perceive possible motivations for reducing meat consumption (positive effects for animal welfare) as more important than men
(Earle et al., 2019) [2]	Experimental, survey; N.D.	U.S. participants N=299, 55,9% women N=280, 57.1% women	Visual reminders of meat's animal origins (vs. images of meat alone) might decrease meat intake willingness via increased	Study 1: (Animal-meat association) Participants complete 4Ns scale (Piazza et al., 2015) prior to manipulation and responded using 7-point	The 4Ns (Piazza et al., 2015); Animal–meat association manipulation; Proanimal emotions; Meat consumption willingness;	Animal–meat reminders led to more proanimal emotions, which in turn predicted less meat consumption willingness. There is a indirect effect of animal–meat reminders	Study 1: animal–meat reminder (vs. meat-alone) condition was associated with greater animal empathy and meat distress and lower meat consumption willingness;

			empathy for animals, distress and disgust about meat	Likert scale; Animal–meat association manipulation: provided three imagens of meat dishes alone; image with lamb paired with prepared lamb chops; image with cow with prepared beef; a image with a pig with prepared ham. Proanimal emotions (Likert scale) Study 2: participants did not complete the 4N scale prior to the manipulation. Was included a pretest measure willingness to exploit animals, antiveg*n prejudice, and	Antiveg*n attitudes	on reducing antiveg*n attitudes via increased empathy for animals. It shows an indirect effect of animal–meat reminders on lower veg*n threat via increased distress about one's meat consumption. There isn´t a total effect of animal–meat reminders on antiveg*n attitudes nor on veg*n threat. There is an effect via animal empathy and meat distress as reminders which predicted less antiveg*n attitudes. Animal–meat reminders fostered greater empathy for	Those higher in right-wing adherence tend to use meat consumption rationalizations; Greater conservatism and 4N endorsement were associated with lower animal empathy, lower meat distress, greater antiveg*n attitudes, and greater meat consumption willingness. Greater RWA was associated with more antiveg*n attitudes. Meat distress and animal empathy were negatively associated with antiveg*n attitudes and meat intake. Willingness; Participants in the animal–meat

				threat reactions to vegetarianism		animals used to produce meat, which in turn predicted less antiveg*n attitudes. There is an indirect effect of animal–meat reminders on veg*n threat via distress, such that animal–meat reminders fostered distress about one's meat consumption. Animal empathy may be particularly for reducing antiveg*n attitudes, whereas distress regarding meat consumption may be important for reducing veg*n threat. Disgust regarding meat may have facilitated beliefs that meat eating is immoral;	reminder condition experienced more animal empathy and meat distress; empathy and distress were associated with less meat consumption willingness, but neither empathy nor distress were related to antiveg*n attitudes. The exposure to animal–meat reminders fostered more animal empathy and meat distress, which in turn predicted lower willingness to eat the meat presented in the Images. The condition had a stronger effect on empathy for the pig relative to

						People may be particularly susceptible to experiencing more proanimal emotions and less meat consumption willingness when faced with pig–pork reminders relative to other meat–animal Pairings;<br><br>Meat–animal associations had a stronger impact on empathy for the pig relative to the cow or lamb in both studies and had a stronger impact on meat distress and meat consumption willingness regarding the pig relative to the lamb in Study 2	lamb, F(1, 297) = 19.57, p < .001, and cow, F(1, 297) = 11.14, p = .001. The condition also had a stronger effect for pig, relative to lamb, on meat distress, F(1, 297) = 4.23, p = .041, and meat consumption willingness, F(1, 297) = 5.88, p = .016.<br>Study 2:<br>SDO was associated with less animal empathy, less meat distress, more antiveg*n attitudes, and greater veg*n threat, but not associated with meat disgust or meat consumption willingness.<br>Veg*n threat was

							positively associated with all three ideology measures and antiveg*n attitudes, and negatively associated with meat distress; Participants in the animal–meat reminder condition experienced more animal empathy, more distress and disgust about meat consumption. Greater empathy was associated with lower meat consumption willingness and less antiveg*n attitudes. Greater distress was associated with lower meat consumption willingness and less veg*n threat. Greater disgust

							was associated with less meat consumption willingness, and unexpectedly, greater veg*n threat there was a significant total effect of animal–meat (vs. meat-alone) condition on meat consumption willingness. There was a indirect effect of animal–meat reminder on Meat consumption willingness, with specific indirect effects via empathy, distress, and disgust, such that exposure to animal–meat reminders fostered more animal empathy, meat distress, and meat

							disgust. Significant effect of condition on threat via meat distress, such that the animal–meat reminders predicted greater distress, which in turn predicted lower perceptions that veg*nism threatens one's cultural practices. There were positive indirect effects of experimental condition on veg*n threat via disgust suggesting animal empathy and distress, animal–meat reminders fostered meat disgust, which in turn, was associated with more veg*n threat

(Haile et al., 2021) [3]	Online survey, 2018-2020	U.S. students, N=338	Can a randomized-controlled trial of pro-vegan animal-welfare pamphlets with students present any significant effects in meat reduction?	A treatment group received a animal-advocacy pamphlet (by Vegan Outreach). The pamphlet discusses the impact of factory farming and the conditions under which farm animals are treated. The pamphlet also contains information on how to eat less meat, i.e., discussions about the health benefits of eating a plant-based diet	"How willing are you to make lifestyle changes to help reduce mistreatment of farm animals?" Options:<br><br>"Not willing…"<br>"Willing to make small changes…"<br>"Willing to make moderate changes…"<br>"Willing to make big changes"	The results show that the animal-advocacy pamphlets had no detectable aggregate effects in the short or long term. The treatment effects of reducing meat in the first semester were rejected by 2.6 percentage points or larger (CI = [−0.026, 0.006]), in the second semester by 2.1 percentage points or larger (CI = [−0.021, 0.023]), and over both semesters by 1.9 percentage points or larger (CI = [−0.019, 0.013]), with 95% confidence	During the semester of the intervention, men significantly decrease their consumption of poultry or fish by 2.4 percentage points (5.2%) and increase their consumption of vegetarian/vegan meals by roughly the same magnitude, 2.3 percentage points (10.6%), suggesting substitution from meat to vegetarian/vegan meals. Overall, meat consumption for men falls by the same magnitude as the decline in poultry/fish, 2.4 percentage points (3.6%). Women, in contrast, significantly

							reduce beef consumption by 1.5 percentage points (12.5%). Poultry and fish consumption increases, though insignificantly, which explains why overall meat consumption does not fall for women. This finding suggests substitution from red meat (beef) to poultry/fish for women
(Hartmann & Siegrist, 2020) [4]	Online survey; 2015	German participants, N=973, 51% women	To determine whether meat-eating justification strategies - unapologetic (pro-meat, denial, hierarchical justification, religious justification, health justification,	No info.	Participants' assessments of how morally justifiable they perceive the following meat production methods to be:<br>Foi gras;<br>Alive boiled lobster;<br>Bull beef from intensive production;	Most of the investigated production methods were perceived as morally not justified.<br>Conventional meat production methods received the most negative evaluations.<br>These negative attitudes towards	The least morally justifiable meat was foie gras, with a mean value of 7.31 on a scale with a maximum value of 8.<br>Only the three meat production methods 'beef from cattle from organic farms', 'free-range

			human destiny and slaughter justification) and apologetic (dichotomization, dissociation And avoidance) - not only exert an effect on meat consumption frequency but also on the moral evaluations of diverse meat production systems		Veal from conventional production; Beef from cattle in tie-stalls; Meat from broilers from intensive production systems; Pork meat from pigs from intensive commercial units; Hunted wild animals (e.g. deer, wild boar, duck); Free-range chicken; Beef from cattle from organic farms; Moral justifiability of various meat production systems; Meat-Eating justification strategies; Food	meat production systems seem to have a very limited effect on meat consumption, and even though people claim to be concerned about welfare issues, the majority does not consider these aspects when consuming or buying meat; These justifications may then be used by consumers to avoid/reduce the negative emotions evoked by gap between ethical convictions and consumer behaviour. Meat-eating justification strategies might also influence the moral justifiability of meat production	chicken' and 'hunted wild animals' were perceived as relatively morally justifiable (with values below the theoretical midpoint of 4.5 on the scale). All the justification strategies except dichotomization were significantly correlated with moral justifiability of the three production systems. People who dissociated animals and food and who avoided negative thoughts about animal husbandry systems and slaughter evaluated the meat production systems more negatively.

					frequency questions; Willingness to substitute (WTS) meat	systems, which explains the observed negative correlation between moral evaluation and unapologetic justification strategies. The endorsement of unapologetic meat-eating justification strategies, such as emphasizing the taste of meat, denial of animal suffering and asserting that animals are lower than humans in the hierarchy, seem to be a driver for resistance. They were linked to more positive moral evaluation of meat production systems, higher meat consumption and	Unapologetic strategies were associated with a more positive moral evaluation of the meat production systems. Males were less morally concerned About conventional meat production compared with females ($\beta=-0.11$, $p < .001$). Both the apologetic ($\beta=0.15$, $p < .001$) and unapologetic ($\beta=-0.43$, $p < .001$) justification strategies were statistically significant predictors. The participants endorsed unapologetic strategies to justify their meat consumption,

						lower<br>WTS meat.<br>The unapologetic justification strategy of health justification was one of the strongest correlates of the frequency of consuming fresh meat and processed meat, and was also associated with lower WTS meat with alternatives.<br>Men are more likely to use unapologetic strategies, while women would rather use indirect, apologetic strategies by dissociating the animal from the meat on their plate and trying to avoid thinking about it.<br>The unapologetic justification	the more likely they were to consider conventional meat production systems as morally justified.<br>The more the participants endorsed the apologetic strategies to justify their meat consumption, the more likely they were to evaluate conventional meat production systems as morally not justified.<br>All unapologetic strategies, dichotomization and avoidance were positively correlated with meat consumption frequency.<br>All strategies except for dissociation and avoidance were

						strategy of health justification was one of the strongest correlates of the frequency of consuming meat and was also associated with lower WTS meat with alternatives	negatively correlated with WTS meat. The highest correlation coefficients were observed for pro-meat (r=−0.51, p < .001), hierarchical justification (r=−0.41, p < .001), health justification (r=−0.39, p < .001) and slaughter justification (r=−0.41, p < .001)
(Herchenroeder et al., 2022) [5]	Online survey; N.D.	U.S. students N=97 N=103; N=97; N=108 (Total N=405)	Video appeal related to animal welfare, environment and health	Video appeal on animal welfare (N=97), environment (N=103), health (N=97) (with documentary film H.O.P.E.), with the intention of reducing meat	If video appeals (on animal welfare, environment, health) can reduce meat intake<br><br>(Likert-scale) Intentions to reduce meat (“I am willing to…”)	The individuals from groups who watched the video expressed greater intentions to reduce meat consumption compared to the control condition (with no video access)	Implicit wanting: there was a significant effect of condition on implicit wanting of meat, $F(3, 401) = 7.41$, $p < .001$, $\eta_p^2 = .053$<br><br>Explicit wanting: there was not a significant effect of condition on

				consumption; The control group (N=108) access a video with no reference to meat consumption impacts			explicit wanting of meat, $F(3,401) = 1.99$, $p = .115$, $\eta p2 = .015$<br><br>Correlations between outcome variables: intentions to reduce meat consumption was negatively correlated with implicit wanting ($r = -.53$, $p < .01$) and explicit wanting ($r = -.48$, $p < .01$)<br><br>The animal welfare condition was associated with higher levels of moral emotions and agreement
(Herrewijn et al., 2021) [6]	Experimental study; 2019	Belgium participants, N=84, 44 women	Does speciesism act as a mediating variable in explaining the impact of a VR	Short 360◦ Documentary - iAnimal: Pig Farms in 360 Degrees - depicting the	(Likert scale) I am willing to prepare less meat during the week', 'I am willing to	The experimental study in which respondents were exposed to Animal Equality's 360◦	VR (vs. video) did have a direct positive effect on presence, a1 = 0.60, β = 0.45,

			experience on empathic concern	life cycle of factory farmed pigs (from their lives on the farm to their death in the slaughterhous e) experienced in a VR format versus in a regular video format Documentary depicts forcefully inseminated and suckling sows; non-sedated castration of male piglets; shows the pigs in cramped and unhygienic crates or spaces, with pathological and aggressive behavior (and sometimes death); they are brought to	prepare smaller portions of meat for each meal'; 'How strongly did you have the feeling to be present in the meat preparation company during the exposure of the images?', 'How strongly did you have the feeling that you were part of the company during the exposure to the images?'	documentary in a VR versus video format showed no significant total effect on participants' intention to reduce meat consumption; Study demonstrate VR (versus video) experience can have on participants' sense of feeling present in the slaughterhouse - that increased the participants' empathic concern for the animals, which subsequently increased their intentions to reduce animal food intake. Speciesism can play a counterproductive role in the persuasive impact of VR	SE = 0.29, t (82) = 2.10, 95% CI [0.03, 1.17], p = .039. Presence had a positive effect on empathic concern, d21 = 0.25, β = 0.37, SE = 0.07, t (81) = 3.54, 95% CI [0.11, 0.39], p < .001, and likewise, VR (vs. video) had an indirect effect on empathic concern via presence, a1d21 = 0.05, BootSE = 0.04, 95% BootCI [<0.01, 0.38]; VR (vs. video) also had a direct, negative effect on empathic concern when the effect of presence was statistically controlled for, a2 = - 0.42, β = - 0.47, SE = 0.19, t

				slaughterhouse they are (not always effectively) stunned by Electrocution; hoisted on a rail hanging upside down and then killed by cutting their throat and letting them exsanguinate At the end of the documentary, a message of Animal Equality reads: 'You don't have to see the world through the eyes of a pig to recognize the cruelty and suffering, but you can see an end to this, please leave meat off your plate.'		(compared to video) messages Involving morally salient topics (the horrible realities of the meat industry) when trying to change people's behavior; Study provides mixed evidence for the effectiveness of Animal Equality's VR (versus video) outreach to promote meat reduction intentions. On the one hand, VR (versus video) might increase consumer intentions to eat less meat because of its positive effect on empathic concern via presence. On the other hand, VR might negatively affect	(81) = - 2.23, 95% CI [-0.80, -0.05], p = .029, suggesting that another mediating variable (speciesism) counteracted the positive mediating effect of presence between medium format and empathic concern; VR (vs. video) positively predicted intentions to reduce meat consumption via presence and empathic concern in serial, a1d21b2 = 0.05, β = 0.04, BootSE = 0.04, 95% BootCI [0.004, 0.31]; VR (vs. video) also had a negative, counteracting

						empathic concern by evoking more speciesist attitudes	effect on meat reduction intentions because of its negative effect on empathic concern, a2b2 =- 0.13, β = -0.12, BootSE = 0.08; 95% BootCI [-0.32, - 0.01], which could explain why we did not find a total effect of VR (vs. video) on participants' meat reduction intentions, R^2 = 3.4%, c = 0.07, SE = 0.23, t (82) = 0.31, 95% CI [-0.38, 0.53], p = .755. Presence only had a positive effect on meat Reduction intentions via empathic concern and did not affect meat reduction intentions

							directly, b1 = 0.14, β = 0.18, SE = 0.09, t (80) = 1.56, 95% CI [-0.04, 0.32], p = .122, so the indirect effect of VR (vs.video) on meat reduction intentions via presence was also not significant, a1b1 = 0.08, β = 0.08, BootSE = 0.08, 95% BootCI [0.07, 0.27]
(Johnson et al., 2021) [7]	Online survey; N.D.	U.S. students, N=58, 34 women; N=146, 70 women	If anthropomorphizing can result in a more favourable treatment towards other animals and result in eating less meat	Study 1: participants were given activities designed to get them to think of a cow or a computer in a more human way. The key measure involved ratings of their intentions to eat various	Study 2: if gender effect found in study 1 would could be generalized to a wider population. Was also included three anthropomorphism conditions for pig, cow, and computer.	Eating intentions following the anthropomorphizing of a cow differed for males and females. Males were more interested in eating beef after humanizing a cow but females showed the opposite pattern of less interest	Study 1: Beef eating intentions- The test comparing intentions to eat beef in the two conditions was not significant (t(56) = 0.46, p = 0.65, d = 0.12); Males in the cow condition (M = 3.46, SD = 0.60) expressed more interest in eating beef than those

				food items in the coming weeks	For study 2 dependent measures were the ratings on willingness to go vegetarian, vegan, beef-free, and pork-free		in the computer condition (M = 2.39, SD = 1.20) (t(22) = 2.82, p = 0.01, d = 1.20), while females tended to show the opposite pattern of less interest in eating beef in the cow condition (M = 2.21, SD = 0.83) than the computer condition (M = 2.80, SD = 1.10) (t(32) = −1.74, p = 0.091, d = 0.62); Females (M = 2.85, SD = 1.12) made more pro-animal decisions than males (M = 3.83, SD = 1.37) (F(1, 54) = 8.39, p = 0.005, h2 p = 0.14); Study 2: Females expressed more willingness to go

							meat-free (M = 2.14, SD = 0.82) than did males (M = 1.78, SD = 0.77) (F(1, 140) = 6.75, p = 0.010, h2 p = 0.05). Females expressed more willingness to try a meat-free diet following anthropomorphism of a cow or pig compared with a computer; Males expressed less willingness to go meat-free in the cow and pig conditions compared with the computer condition
(Kunst & Haugestad, 2018) [8]	Online survey: N.D.	Ecuadorian and U.S. participants; N=183, 58% women; N=178, 42% women	To experimentally Investigate whether culturally variant exposure to unprocessed meat	Participants were randomly assigned to one of two conditions. In both conditions, participants were told that	(Likert-scale) "The first thing I thought about when I saw the picture above was a living being."<br><br>"The meat	Presenting the head of the pork roast led to less dissociation, more disgust and more empathy for participants reporting high (þ1 SD), but	Participants from Ecuador reported a substantially higher exposure to meat products that still show the animal's head, M ¼ 2.63, SD ¼ 1.96, than

			moderates the effects that dissociation has on willingness to eat meat:<br><br>(a) showing the animal head should reduce dissociation to less of a degree in Ecuador than in the US and (b) that this reduced dissociation should lead to more disgust and empathy particularly in the US.	They were going to be presented with a picture of a pork roast. In the head condition, the pork roast's head was visible, while it was removed using photo-editing software in the beheaded condition;<br><br>For both conditions, the picture was identical in all respects except for the difference that the head was shown or not shown.	“displayed in the picture once was part of a living being.”<br><br>“How much does the picture above remind you of a living being?” “When I see the picture above, I feel sorry for the animal that was slaughtered” “Thinking about the animal that was slaughtered to produce the meat displayed above does not disturb me a great deal” (reversed), “Seeing the picture makes me feel pity for the animal that	especially for those reporting low (1 SD) exposure to unprocessed meat. Moreover, presenting the head led to a decreased willingness to eat meat among participants with low exposure to unprocessed meat but not among those with high exposure;<br><br>showing the head of the pork roast reduced dissociation in both countries, leading to heightened disgust and empathy and, consequently, less willingness to eat the meat and more willingness to choose vegetarian;	participants from the US did, M ¼ 0.87, SD ¼ 1.28; t(304.71) ¼ 10.11, p < 0.001, 95% CI of the difference [1.42, 2.11], Cohen's d ¼ 1.06. This supported the expectation that participants from both cultures differed markedly in their exposure to unprocessed meat.<br><br>For both Ecuadorian and US participants, presenting the head decreased dissociation, led to more empathy and disgust and a higher willingness to choose vegetarian. However, these effects were less marked in the

					was slaughtered”, “I feel sad for the animal that died to produce the meat above” “I do not really feel very sorry for the animal that had to die”<br><br>Disgust.<br><br>Willingness to eat meat and to choose vegetarian.<br><br>Exposure to unprocessed meat	While dissociation seems to affect willingness to eat meat across cultures to some extent, it does so especially in societies where consumers are less exposed to unprocessed meat on a daily basis, leaving them more sensitive to cues linking meat to animal origins	Ecuadorian sample than in the US American sample; Results showed that presenting the head led to a lower willingness to eat meat because it reduced dissociation and subsequently increased disgust in Ecuador, B ¼ 1.62, 95% CI [3.58, 0.62], but especially in the US, B ¼ 8.50, 95% CI [14.52, 3.83]
(Kunst & Hohle, 2016) [9]	Online survey; N.D	Norwegian participants N=288, 61.1% women<br>U.S. participants N=168, 43.5% women<br>N=90, 60.4%	How processes of dissociation reduce empathy, disgust, and thereby increase willingness to	Study 1: Chicken is presented at different processing stages (low, medium, high);<br>Study 2a/b: Pork roast is	1. Empathy and state dissociation;<br>2a. Empathy, Perceived mental capacity, Willingness to eat meat,	Using a variation of scenarios with real-world stimuli and simulated consumer-choice situations, this research demonstrated that culturally	Study 1: participants in the high processing condition showed more state dissociation (M ¼ 5.33, SE ¼ 0.13) than in the

		women N=187, 56.7 women	eat meat; Moreover, potency of these processes increase by a reduced attribution of mind to animals	presented with/without head; Study 3: Lamb chops advertisement is presented with/without lamb (Control Condition and lamb present condition; Study 4: Mass slaughter of cows is presented as either (Slaughtered Killed, Harvested); Study 5: Restaurant menu is presented with meat terms or animal terms	State dissociation 2b: Empathy, Disgust, Willingness to eat meat, Willingness to consider vegetarian alternative, State dissociation; 3: Trait dissociation, Empathy, Perceived mental capacity, Willingness to eat meat, State Dissociation; 4: Empathy, State dissociation; 5: Trait dissociation, Empathy, Disgust, Willingness to eat meat, Willingness to consider vegetarian	entrenched processes of dissociation found in the way we produce, prepare and talk about meat and animals sustain people's willingness to eat meat as they make it easy to ignore the meat-animal link. Such dissociation reduces empathy and disgust that would otherwise reduce meat consumption	low processing condition (M ¼ 4.10, SE ¼ 0.14; p < 0.001, 95% CI of difference [0.85, 1.61]). Participants in the high processing condition reported less empathy towards the slaughtered animal (M¼ 2.91, SE ¼ 0.17) than those in the low processing condition (M ¼ 3.41, SE ¼ 0.18; p ¼ 0.045, 95% CI of difference [0.98, 0.01]). Study 2a: The beheaded condition produced a substantial drop in empathy, t(166) ¼ 4.94, p < 0.001, increased state dissociation, t(166) ¼ 7.52, p < 0.001 and

					alternative, State Dissociation		willingness to eat the meat t(166)¼3.83, p < 0.001 (see Fig. 4). Study 2b: Participants showed more state dissociation, t(99) ¼ 5.04, p < 0.001, less empathy, t(99) ¼ 3.51, p ¼ 0.001, and less disgust, t(99) ¼ 4.32, p < 0.001, when the pork roast was beheaded than when the head was part of the roast. Moreover, they showed a higher willingness to eat meat, t(99) ¼ 2.77, p ¼ 0.007 and were marginally significantly less likely to consider a vegetarian alternative—with head: M ¼ 52.00,

							SE ¼ 5.56; beheaded: M ¼ 37.88, SE ¼ 5.11; t(99) ¼ 1.87, p ¼ 0.065. The experimental manipulation led to less empathy (b ¼ 0.29, SE ¼ 0.06, 95% CI [0.43, 0.17], p ¼ 0.001) and disgust (b ¼0.22, SE ¼ 0.07, 95% CI [0.36, 0.12], p < 0.001), mediated by state dissociation. Because empathy and disgust predicted less willingness to eat meat and a higher likelihood to choose the vegetarian alternative, the experimental manipulation had an indirect positive effect on willingness to eat meat (b ¼ 0.31,

							SE ¼ 0.07, 95% CI [0.18, 0.45], p < 0.001) and an indirect negative effect on likelihood to choose vegetarian (b ¼ 0.29, SE ¼ 0.06, 95% CI [0.41, 0.18], p < 0.001). Study 3: participants showed more empathy, t(185) ¼ 3.51, p ¼ 0.001, and less state dissociation, t(185) ¼ 6.67, p < 0.001, when the lamb was presented in the advertisement. they were less willing to eat meat when the lamb was presented than when it was not presented, t(185) ¼ 3.33, p ¼ 0.001. Women showed more

							trait dissociation (M ¼ 4.79, SD ¼ 1.72) but less state dissociation (M ¼ 3.52, SD ¼ 1.64) than men did — trait dissociation: M ¼ 4.10, SD ¼ 1.67, t(185) ¼ 2.74, p ¼ 0.007; state dissociation: M ¼ 4.15, SD ¼ 1.44, t(185) ¼ 2.73, p ¼ 0.007. Portrayals of animals in meat advertisements increase empathy as a consequence of reduced dissociation. When a living lamb was presented alongside lamb chops in an advertisement, participants showed more empathy towards the animal that had to die to

							produce the chops, and this relation was fully due to a decrease in dissociation. Participants were less willing to eat the lamb chops when the animal was presented Study 4: participants differed in the degree to which the words reminded them of the fact that the animals had been living beings, F(2, 290) ¼ 17.39, p < 0.001, hp 2 ¼ 0.11. LSD post-hoc tests showed that those in the harvested condition showed more state dissociation than those in the kill and slaughter conditions. the

							experimental dummy variable (coded as:<br>0 ¼ merged ‘killed’ and ‘slaughtered’ condition, 1 ¼ ‘harvested’ condition) led to more state dissociation, which was related to less empathy (b ¼ 0.53, p < 0.001).<br>Study 5:<br>State dissociation decreased, t(188) ¼ 5.49, p < 0.001, while empathy, t(188) ¼ 2.80, p ¼ 0.005, and disgust, t(188) ¼ 3.59, p < 0.001, increased when “beef/pork” were replaced with “cow/ pig” in the restaurant menu. Willingness to eat the dishes displayed in the menu dropped

							once the animal words were used, t(188) ¼ 3.59, p < 0.001 Participants were significantly more likely to consider a vegetarian alternative when the animal labels were used (M ¼ 43.12, SE ¼ 3.84) than when “beef” and “pork” were used (M ¼ 33.78, SE ¼ 3.49), t(188) ¼ 1.80, p ¼ 0.074. the experimental manipulation had an indirect and inverse effect on willingness to eat the meat dishes (b ¼ 0.19, SE ¼ 0.04, 95% CI [0.28, 0.12], p < 0.001) and an indirect positive effect on likelihood to eat vegetarian (b ¼ 0.17, SE ¼ 0.04, 95% CI [0.11,

							0.25], p < 0.001).
(Palomo-Vélez et al., 2018) [10]	Questionnaire, and recall test, 2017	U.S: participants N=309, 58.5% women	The effect of persuasive messages / moral appeals related to animal welfare, and on attitudes toward meats and vegetables	Essay excerpt: "Up to 10 distressed and suffering birds are shoved into a single small cage, in one huge shed with up to 90,000 other chickens…"	To rate meat or vegetables after reading essay on animals farming conditions	The desire to eat vegetables was higher than the desire to eat meats only in the moral condition, $t(75)=-2.37$, $p = .02$, $d=-0.42$, and disgust condition, $t(78)=-3.76$, $p < .001$, $d=-0.66$	No info.
(Piazza et al., 2018) [11]	Questionnaire; 2016-2018	U.S. omnivores, N=168, 68 women	How willing participants would be to eat the meat depicted in the photograph Associating (through images) baby animals to meat may reduce appetite for meat	Study 1: images of baby animals, versus adult animals, as the source of meat; Study 2: replicated study 1 using a larger sample and two new animal sources Study 3: only a baby animal presented (no meat)	Testing the demotivating influence of baby animals on appetite for meat	Meat sourced from a baby animal was rated overall less appetizing (M = 49.28, SD = 32.91) than the same meat sourced from an adult animal (M = 59.42, SD = 31.83).	Collapsing across gender, the meat was least appetizing when it was presented along with an image of a baby animal (M = 59.38, SD = 35.14) as the source, and most appetizing when it was presented without any image of the animal source (M= 76.89, SD = 25.99), with the

							adult animal source falling in between (M = 71.56, SD = 27.17)
(Tian et al., 2016) [12]	Questionnaire, (online in France); N.D.	French and Chinese participants, N=520, 176 women<br><br>*Only French data included in this extraction table (see inclusion criteria)	Focusing on meat production and meat consumption was examined whether participants used reduction of willingness to eat meat and reduction of mind attribution to food animals as strategies to reduce cognitive dissonance from the meat paradox	Study 1: Abattoir condition: photo of a cow with a short statement saying that the cow will be sent to the abattoir tomorrow. Pasture condition: photo of a cow with a statement that the cow will be sent to another pasture tomorrow. Meat condition: diagram of a cow that displays the names of the different	The meat paradox in the meat production stage: abattoir condition; pasture condition; meat condition; control condition	Cognitive dissonance in response to the meat paradox is observed among French participants. Study 1: focused on the meat production stage, found that the abattoir condition led most participants to think about the slaughter. The results that participants reduced their willingness to eat beef and that reduced their mind attribution to cows when the animal origin of meat was explicitly shown in the pasture;	Study 1: Manipulation check - 86% (98% French) participants in the abattoir condition mentioned the killing, 47% did so in the pasture condition (38%);. Participants who mentioned the slaughter did not report less willingness to eat beef, and did not attribute less mind to cows (Mwillingness ¼ 3.58, SDwillingness ¼ 1.35; Magency ¼ 27.63, SDagency ¼ 7.19; Mexperience ¼ 36.27, SDexperience ¼

				kinds of beef from the various parts of the cow's body; Control condition had no experimental manipulation Study 2: recipe in a short text, the recipe with animal image condition showed a photo of a cow, illustrating the source of beef in the dish, and the recipe with dish image condition showed a photo of the dish; the recipe alone condition described the recipe only in a short text; the		Meat conditions supported the hypothesis that people would reduce their willingness to eat meat and/or mind attribution to animals to resolve cognitive dissonance resulting from the meat paradox. Study 2: focused on the meat consumption stage, revealed that the recipe with animal image condition led participants to like a dish less, compared with the recipe with dish image and recipe alone conditions. The marginally significant findings that participants	5.87) than those who did not mention the slaughter (Mwillingness ¼ 3.67, SDwillingness ¼ 1.39; Magency ¼ 27.78, SDagency ¼ 6.61; Mexperience ¼ 36.61, SDexperience ¼ 5.53), ps > .10. Willingness to eat meat (beef); Participants in the abattoir condition (M ¼ 3.41, SD ¼ 1.39) were less willing to eat beef than those in the meat condition (M ¼ 3.90, SD ¼ 1.44), p ¼ .034. French participants in the condition where willingness to eat meat was measured first (M ¼ 3.56, SD ¼

				control condition did not present the recipe but moved directly on to the dependent measures after showing a sentence of acknowledgment for participation		reported less willingness to eat meat and attributed less mind to cows when the animal origin of meat was made salient compared to the recipe alone condition gave partial support to the hypothesis that people would reduce their willingness to eat meat and mind attribution to cows as strategies to deal with the dissonance resulting from the meat paradox	1.23) were less willing to eat beef than those in the condition where mind perception of cows was measured first (M ¼ 4.04, SD ¼ 1.31), F(1,515) ¼ 7.65, p ¼ .006; Mind perception of cows - French participants did show significant differences between the dissonance manipulation conditions, F(1, 498) ¼ 3.91, p ¼ .009. French participants in the pasture condition (p ¼ .069) and meat condition (p ¼ .088) attributed less agency to cows than those in the control condition: French participants

							showed a marginally significant tendency to attribute less mind to cows on the dimension of agency after the animal origin of meat had been made explicit in the pasture and meat conditions, compared to the control condition. French participants were significantly less willing to eat beef when the willingness to eat meat was measured first than when it was measured second Study2: Participants in the recipe with animal image condition (M ¼ 4.63, SD ¼ 1.97) reported less liking than those

							in the recipe with dish image (M ¼ 5.39, SD ¼ 1.71) (p ¼ .001) and recipe alone conditions (M ¼ 5.60, SD ¼ 1.34), p < .001, leading us to think that greater cognitive dissonance had been induced in the recipe with animal image Condition; French participants reported hunger (M ¼ 2.80, SD ¼ 1.83), F(1,510) ¼ 7.07, p ¼ .008, h2P ¼ .014. Participants in the recipe with animal image condition (M ¼ 3.75, SD ¼ 1.30) were less willing to eat beef than those in the recipe alone condition (M ¼ 4.13, SD ¼ 1.26), p ¼ .085.

							Compared to those in the control condition (M ¼ 3.70, SD ¼ 1.29), Participants were more willing to eat beef in the recipe alone condition (M ¼ 4.13, SD ¼ 1.26), p ¼ .045. Participants in the recipe with animal image condition (M ¼ 26.18, SD ¼ 7.07) and in the recipe with dish image condition (M ¼ 25.97, SD ¼ 7.15) attributed less mind to cows than those in the recipe alone condition (M ¼ 28.13, SD ¼ 7.23), p's ¼ .10 and .054
(Zickfeld et al., 2018) [13]	Questionnaire, exploratory; N.D.	U.S. and Norwegian participants, N=253, 117	If cuter animals would reduce the willingness to	Study 1: (U.S. participants N=253, 117 women)	Cuteness can reduce meat consumption and increase	Evidence support the view that increased cuteness results	Study 1: Willingness to eat meat was higher in the control

		women; N=407, 199 women N=306, 133 women N=108, 84 women N=821, (Total N=1074)	consume meat and increase empathy towards the animals	If presenting a cute-looking lamb in an advertisement results in reduced self-reported intention to consume meat; Study 2: (U.S: participants N= 407, 199 women) By manipulating the cuteness of a presented animal, and testing its direct effect on willingness to eat meat; Study 3a (U.S. participants, N=306, 133 women): replicated the findings in study 2 with Norwegian students; Study 3b	empathy towards the animals;	in less willingness to consume meat in the US, but to a lesser extent in Norway. Results from all studies were consistent in that the effect of cuteness on willingness to eat meat was mediated by empathy towards the animal	condition when the animal was not present; Cuteness responses towards the imagined lamb differed across the two conditions, with participants in the experimental condition giving higher cuteness ratings than those in the control condition did; humanness ratings for the lamb also differed across the two conditions, with participants in the experimental condition giving higher humanness ratings than those in the control condition; Study 2: Participants characterized the

				(Norwegian students N=108, 84 women): replicated study 3a with Norwegian students (Studies 3a and 3b included lamb vs. calf vs. pig)			lamb in the cute condition as cuter than the lamb in the neutral condition;<br>Study 3a and study 3b: main effect both for condition, F(1, 244) ¼ 47.65, p < 0.001, and animal type, F(2,211) ¼ 24.95, p < 0.001, but not for the interaction, F(2, 243) ¼ 1.71, p ¼ 0.184. As in Study 3a, the cute condition evoked higher cuteness ratings than the non-cute condition.<br>In contrast to Study 3a, willingness to eat meat was lowest for the pig, but did not differ between the lamb and calf. In addition, as

							opposed to findings in Study 3a, participants in the cute condition were on average not less likely to consume the meat than people in the control condition

References

1. Cordts, A.; Nitzko, S.; Spiller, A. *Consumer Response to Negative Information on Meat Consumption in Germany*; 2014; Vol. 17;.
2. Earle, M.; Hodson, G.; Dhont, K.; MacInnis, C. Eating with Our Eyes (Closed): Effects of Visually Associating Animals with Meat on Antivegan/Vegetarian Attitudes and Meat Consumption Willingness. *Gr. Process. Intergr. Relations* **2019**, *22*, 818–835, doi:10.1177/1368430219861848.
3. Haile, M.; Jalil, A.; Tasoff, J.; Vargas Bustamante, A. Changing Hearts and Plates: The Effect of Animal-Advocacy Pamphlets on Meat Consumption. *Front. Psychol.* **2021**, *12*, doi:10.3389/fpsyg.2021.668674.
4. Hartmann, C.; Siegrist, M. Our Daily Meat: Justification, Moral Evaluation and Willingness to Substitute. *Food Qual. Prefer.* **2020**, *80*, doi:10.1016/j.foodqual.2019.103799.
5. Herchenroeder, L.; Forestell, C.A.; Bravo, A.J. The Effectiveness of Animal Welfare-, Environmental-, and Health-Focused Video Appeals on Implicit and Explicit Wanting of Meat and Intentions to Reduce Meat Consumption. *J. Soc. Psychol.* **2022**, doi:10.1080/00224545.2022.2081529.
6. Herrewijn, L.; De Groeve, B.; Cauberghe, V.; Hudders, L. VR Outreach and Meat Reduction Advocacy: The Role of Presence, Empathic Concern and Speciesism in Predicting Meat Reduction Intentions. *Appetite* **2021**, *166*, 105455, doi:10.1016/j.appet.2021.105455.
7. Johnson, C.; Schreer, G.; Bao, K.J. Effect of Anthropomorphizing Food Animals on Intentions to Eat Meat. *Anthrozoos* **2021**, *34*, 563–578, doi:10.1080/08927936.2021.1914442.
8. Kunst, J.R.; Palacios Haugestad, C.A. The Effects of Dissociation on Willingness to Eat Meat Are Moderated by Exposure to Unprocessed Meat: A Cross-Cultural Demonstration. *Appetite* **2018**, *120*, 356–366, doi:10.1016/j.appet.2017.09.016.
9. Kunst, J.R.; Hohle, S.M. Meat Eaters by Dissociation: How We Present, Prepare and Talk about Meat Increases Willingness to Eat Meat by Reducing Empathy and Disgust. *Appetite* **2016**, *105*, 758–774, doi:10.1016/j.appet.2016.07.009.

10. Palomo-Vélez, G.; Tybur, J.M.; van Vugt, M. Unsustainable, Unhealthy, or Disgusting? Comparing Different Persuasive Messages against Meat Consumption. *J. Environ. Psychol.* **2018**, *58*, 63–71, doi:10.1016/j.jenvp.2018.08.002.
11. Piazza, J.; McLatchie, N.; Olesen, C. Are Baby Animals Less Appetizing? Tenderness toward Baby Animals and Appetite for Meat. *Anthrozoos* **2018**, *31*, 319–335, doi:10.1080/08927936.2018.1455456.
12. Tian, Q.; Hilton, D.; Becker, M. Confronting the Meat Paradox in Different Cultural Contexts: Reactions among Chinese and French Participants. *Appetite* **2016**, *96*, 187–194, doi:10.1016/j.appet.2015.09.009.
13. Zickfeld, J.H.; Kunst, J.R.; Hohle, S.M. Too Sweet to Eat: Exploring the Effects of Cuteness on Meat Consumption. *Appetite* **2018**, *120*, 181–195, doi:10.1016/j.appet.2017.08.038.
